# Safety, Acceptability and Adherence of Dapivirine Vaginal Ring in a Microbicide Clinical Trial Conducted in Multiple Countries in Sub-Saharan Africa

**DOI:** 10.1371/journal.pone.0147743

**Published:** 2016-03-10

**Authors:** Annalene Nel, Linda-Gail Bekker, Elizabeth Bukusi, Elizabeth Hellstrӧm, Philip Kotze, Cheryl Louw, Francis Martinson, Gileard Masenga, Elizabeth Montgomery, Nelisiwe Ndaba, Ariane van der Straten, Neliëtte van Niekerk, Cynthia Woodsong

**Affiliations:** 1 International Partnership for Microbicides, Silver Spring, Maryland, United States of America; 2 The Desmond Tutu HIV Foundation, University of Cape Town, Cape Town, South Africa; 3 Kenya Medical Research Institute, Kisumu, Kenya; 4 Be Part Yoluntu Centre, Paarl, South Africa; 5 Qhakaza Mbokodo Research Clinic, Ladysmith, South Africa; 6 Madibeng Centre for Research, Brits, South Africa; 7 University of North Carolina Project, Lilongwe, Malawi; 8 Kilimanjaro Christian Medical Centre, Moshi, Tanzania; 9 RTI International, San Francisco, California, United States of America; 10 Maternal, Adolescent and Child Health, Edendale, South Africa; University of Ottawa, CANADA

## Abstract

**Background:**

This was the first microbicide trial conducted in Africa to evaluate an antiretroviral-containing vaginal ring as an HIV prevention technology for women.

**Objectives:**

The trial assessed and compared the safety, acceptability and adherence to product use of a 4-weekly administered vaginal ring containing the antiretroviral microbicide, dapivirine, with a matching placebo ring among women from four countries in sub-Saharan Africa.

**Methods:**

280 Healthy, sexually active, HIV-negative women, aged 18 to 40 years were enrolled with 140 women randomised to a dapivirine vaginal ring (25 mg) and 140 women to a matching placebo ring, inserted 4-weekly and used over a 12-week period. Safety was evaluated by pelvic examination, colposcopy, clinical laboratory assessments, and adverse events. Blood samples for determination of plasma concentrations of dapivirine were collected at Weeks 0, 4 and 12. Residual dapivirine levels in returned rings from dapivirine ring users were determined post-trial. Participant acceptability and adherence to ring use were assessed by self-reports.

**Results:**

No safety concerns or clinically relevant differences were observed between the dapivirine and placebo ring groups. Plasma dapivirine concentrations immediately prior to ring removal were similar after removal of the first and third ring, suggesting consistent ring use over the 12-week period. No clear relationship was observed between the residual amount of dapivirine in used rings and corresponding plasma concentrations. Self-reported adherence to daily use of the vaginal rings over the 12-week trial period was very high. At the end of the trial, 96% of participants reported that the ring was usually comfortable to wear, and 97% reported that they would be willing to use it in the future if proven effective.

**Conclusions:**

The dapivirine vaginal ring has a favourable safety and acceptability profile. If proven safe and effective in large-scale trials, it will be an important component of combination HIV prevention approaches for women.

**Trial Registration:**

ClinicalTrials.gov NCT01071174

## Introduction

The global fight against the human immunodeficiency virus (HIV) epidemic has shown enormous progress over the past decade but despite these efforts, HIV infection rates among women worldwide remains very high and African women are disproportionately affected [1, 2]. According to UNAIDS, sub-Saharan Africa remains one of the most severely affected regions in the world; 80% of the 16 million women aged 15 years and older who were infected with HIV by the end of 2013 are living in sub-Saharan Africa [1]. Developing safe and effective woman-initiated HIV prevention technologies that can be made easily accessible in developing countries is a public health priority.

Antiretroviral containing vaginal rings that can provide sustained release of anti-HIV microbicides over a period of time can offer at-risk women a discreet prevention option that they can initiate. Adherence to product use, however, remains a critical factor, as microbicides need to be used consistently in order to be effective. Participant adherence has proven to be a key challenge in vaginal microbicide trials [3], with efficacy studies of antiretroviral-containing gel formulations reporting significant non-adherence to product use [4, 5]. Difficulties in achieving, as well as measuring participant adherence to vaginal gel use have strengthened interest in the development of vaginal rings, as this delivery form requires minimal user action and is not daily or coitally-dependent. There is also interest in the development of measures that could provide a more objective adherence assessment than participant self-report, which has been shown to over-estimate adherence [6]. Such measures of vaginal ring adherence include drug concentrations in plasma, vaginal fluids and vaginal tissue as well as residual drug levels in used vaginal rings. In addition to these methods, recently published results from *in vitro* and non-clinical studies performed in macaques on the use of a silicone elastomer vaginal ring prototype containing an embedded miniature temperature logger, demonstrated potential for these devices to serve as an accurate and reliable method to continuously monitor environmental temperature and determine episodes of ring insertion and removal [7].

The International Partnership for Microbicides (IPM) has developed a vaginal ring (Ring-004) containing the microbicide, dapivirine, a non-nucleoside reverse transcriptase inhibitor with potent HIV-1 inhibitory activity *in vivo* and *in vitro*. Four Phase I trials of this dapivirine Ring-004, conducted in Europe, demonstrated a safety and pharmacokinetic profile supporting the ring’s use for HIV-1 prevention [8–11]. The dapivirine Ring-004 is currently being tested in two Phase III safety and efficacy vaginal ring clinical trials in Malawi, South Africa, Uganda and Zimbabwe with results expected in 2016.

The clinical trial reported in this paper was the first microbicide vaginal ring trial conducted in Africa to evaluate the safety and acceptability of the dapivirine ring as an HIV prevention technology. The trial enrolled women from Kenya, Malawi, South Africa and Tanzania, randomised in a 1:1 ratio to either an active dapivirine vaginal ring or a placebo ring, inserted 4-weekly and used for a period of 12 weeks. Here we report on the safety of the dapivirine vaginal ring after 12 weeks of continuous ring use, participants’ adherence to product use and acceptability of the vaginal ring.

## Methods

### Trial Design

This Phase I/II double-blind, randomised, placebo-controlled trial was conducted from April 2010 to May 2011 at ten research centres in Kenya, Malawi, South Africa and Tanzania. A total of 280 participants were enrolled in the trial with 140 participants randomised to each treatment group. Each participant received three vaginal rings over the course of the treatment period. The rings were inserted at 28-day intervals over a treatment period of 12 weeks at Weeks 0, 4, and 8. Participants were eligible for enrolment if they were generally healthy, HIV-negative, sexually active, tested negative for pregnancy, willing to use a stable form of contraception during the trial (oral contraceptives, transdermal patches, long-acting injectable progestins, intrauterine devices or had a surgical sterilization), willing to refrain from using vaginal products or objects (including tampons), had a normal appearing cervix and vagina based on pelvic examination and colposcopy, and normal Pap test results. The use of female condoms was not allowed during the trial because mechanical compatibility of the dapivirine vaginal ring device with a female condom was not yet established.

Relevant medical and surgical history, obstetric and gynaecological history including history of menses and demographic data were collected at the screening visit. Information on baseline behavioural data was collected at the enrolment visit. Vaginal ring insertion and removal was done by the participants at the research centre under supervision of the Investigator who, after insertion of the ring, examined that the ring was properly placed. After insertion of the first ring on the day of enrolment, participants remained under observation for 30 minutes at the research centre to assess any immediate adverse reactions. Research staff provided participants with vaginal ring adherence counselling and a ring diary card to document details of ring removals and accidental ring expulsions. Participants were instructed that if an inserted vaginal ring came out, *e*.*g*., during intercourse or exercise, the participant was to wash her hands, rinse the ring thoroughly in lukewarm water and re-insert it. If the ring was contaminated or damaged, she was instructed to return to the research centre for a replacement ring. Women were asked to continue ring use during menses.

### Safety Assessments

The safety of the vaginal rings was assessed through pre-specified clinical safety endpoints that included pelvic/colposcopy examinations, clinical laboratory testing (haematology, liver and renal function, and urinalysis), diagnostic sexually transmitted infection (STI) testing for trichomonas, gonorrhoea and/or chlamydia, HIV testing, pregnancy testing, sample collection of vaginal flora and vaginal pH measurement, and adverse event reporting. A final follow-up safety assessment was conducted 4 weeks after removal of the last ring. HIV/STI risk reduction counselling, including the dispensing of male condoms were provided to participants at each trial visit.

Urinalysis, urine pregnancy tests and wet mount assessments were performed at the research centres. All other laboratory analyses were done at the certified central laboratory.

### Dapivirine Concentrations in Plasma and Residual Dapivirine Levels in Returned Rings

Blood samples for determination of plasma dapivirine concentrations were collected prior to insertion of the first ring, and immediately before ring removal after 4 and 12 weeks of vaginal ring use. Residual dapivirine levels were measured in the rings returned at Weeks 4, 8 and 12.

Plasma samples were processed and analysed for dapivirine using a validated high-pressure liquid chromatography tandem mass spectroscopy (HPLC-MS/MS) method [12]. Residual dapivirine levels in the active rings were measured using a validated HPLC method [13].

### Participant Acceptability and Adherence to Vaginal Ring Use

Participant acceptability of the vaginal rings and adherence to ring use were assessed via interviewer-administered questionnaires. Acceptability questionnaires were completed at the enrolment visit, Week 4 and Week 12 and included questions about the participant’s sexual relationship(s), experiences with the vaginal ring including physical comfort, preferences and concerns, willingness to use the vaginal ring if proven effective against HIV-1 infection, and perceptions of male partners’ experiences and attitudes.

Adherence questionnaires were completed at Weeks 2, 4, 8, and 12 and assessed sexual behaviour, vaginal practices, male condom use, vaginal ring removals or expulsions, the circumstances of each removal/expulsion event, duration of time during which the ring was out of the vagina, if intercourse occurred during this event, and if the ring was reinserted. To lessen the possible impact of adherence counselling on participant self-reported use, adherence questionnaires were administered to the participants prior to providing them with adherence counselling. The diary cards were used by participants as a memory aid for their responses to adherence questions.

### Investigational Products

Ring-004 contained 25 mg of dapivirine dispersed in a platinum-catalysed silicone matrix with an outer diameter of 56 mm and a cross-sectional diameter of 7.7 mm. To maintain the double-blind identity of the trial, a matching placebo ring without dapivirine was used that was similar in composition to Ring-004 except that it contained titanium dioxide as a colorant to match the appearance of Ring-004.

### Ethics

Written informed consent was obtained from each participant prior to any trial-related procedures. Ethics and regulatory approvals were obtained from the appropriate ethics committees and government medicines regulatory bodies for each research centre in the respective countries. The following ethics committees provided regulatory approval in the respective countries: South Africa (Pharma Ethics, University of Cape Town’s Health Sciences Faculty, Health Research and Knowlegde Management KwaZulu-Natal Department of Health, University of the Witwatersrand Human Subjects Research Ethics Committee, North West Knowledge Management and Research Committee), Kenya (Kenya Medical Research Institute), Tanzania (National Institute for Medical Research, Kilimanjaro Christian Medical Centre Research and Ethics Committee, and the London School of Hygiene and Tropical Medicine), Malawi (National Health Sciences Research Committee). The trial was conducted in full compliance with the ICH-GCP guidelines, and was registered in the ClinicalTrials.gov database (NCT01071174).

### Statistical Analyses

Statistical analyses were conducted on the intent-to-treat (ITT) population (defined as all participants randomised to one of the two treatment groups). Adverse events were coded using version 10.0 of the Medical Dictionary for Regulatory Activities (MedDRA). Only treatment emergent adverse events (TEAEs) are presented in the paper. Safety variables, dapivirine concentrations in plasma, as well as residual levels of dapivirine in returned rings were summarized using appropriate descriptive statistics. Risk ratios (RRs) with corresponding 95% confidence intervals (CIs) comparing the proportion of participants diagnosed with events post-baseline between the dapivirine and placebo ring groups were calculated for each of the pre-specified safety endpoints and the composite safety endpoint. For the acceptability and adherence analysis, data were evaluated by treatment group, for both groups combined, and by trial visit.

## Results

### Participant Disposition

**Fig 1:**
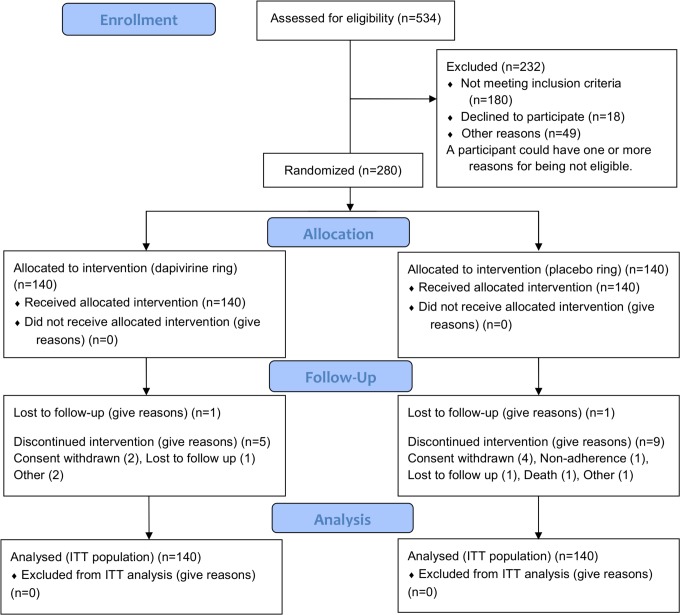
Participant Flowchart.

Two-hundred-and-eighty (280) participants were enrolled across the ten research centres in Kenya, Malawi, South Africa, and Tanzania (Table 1). One-hundred-and-forty (140) participants were randomised to each treatment group (Fig 1). Of these, 266 (95.0%) participants completed the trial and 14 (5.0%) participants discontinued early. Six participants withdrew their consent and two participants were lost to follow up. Other reasons for early trial discontinuation, reported for one participant each, included relocation outside the trial area, non-adherence to trial procedures, missed final trial visit at Week 16, inappropriate enrolment, and investigational product dispensing error. One participant in the placebo ring group died due to haemopneumothorax that occurred secondary to a physical assault.

**Table 1:** Participant Enrolment at Research Centres.

Research Centre	Number of Participants Enrolled
Be Part Yoluntu Centre (Paarl, South Africa)	40
Desmond Tutu HIV Foundation, Masiphumelele Clinic (Cape Town, South Africa)	28
Desmond Tutu HIV Foundation, Emavundleni Centre (Cape Town, South Africa)	30
Kenya Medical Research Institute (Kisumu, Kenya)	20
Kilimanjaro Christian Medical Centre (Moshi, Tanzania)	9
University of North Carolina Project (Lilongwe, Malawi)	16
Madibeng Centre for Research (Brits, South Africa)	40
Prevention for HIV and AIDS Project (Pinetown, South Africa)	17
Maternal, Adolescent and Child Health (Edendale, South Africa)	40
Qhakaza Mbokodo Research Clinic (Ladysmith, South Africa)	40

### Demographics, Relationship Status and Sexual History

The two treatment groups were similar in respect to demography and baseline characteristics (Table 2). The majority of women in the trial were of Black ethnicity comprising 89% of the trial population. Four percent of the women were of mixed ethnicity, defined as “Coloured” in South Africa. Seven percent of the women chose not to indicate their ethnicity. In both treatment groups the mean age was 25 years and ranged from 18 to 40 years.

**Table 2:** Demographic and Other Baseline Behavioural Characteristics. HIV = Human immunodeficiency virus; SD = Standard deviation.

Characteristic		Dapivirine Vaginal Ring(N = 140)	Placebo Vaginal Ring(N = 140)
		n (%)	n (%)
Race*			
	Black	124 (89%)	124 (89%)
	Coloured**	6 (4%)	6 (410 (7%))
	Declined to answer	10 (7%)	
**Age (years)**			
	Mean (SD) / range	25.8 (5.58) / (18–39)	25.4 (5.36) / (18–40)
**Education**			
	Primary education completed	135 (96%)	133 (95%)
	Secondary education completed	68 (49%)	75 (54%)
	Tertiary education completed	7 (5%)	4 (3%)
**Relationship Status**			
	Married	31 (22%)	30 (21%)
	Single	84 (60%)	89 (64%)
	Divorced	1 (1%)	0 (0%)
	Widowed	1 (1%)	1 (1%)
	One partner	137 (98%)	139 (99%)
	Lives with partner	47 (34%)	51 (36%)
**Sex partner characteristics**			
	Has main sex partner	139 (100%)	138 (100%)
	Has other sex partners	8 (6%)	8 (6%)
**Live with a sex partner**			
	All the time	47 (34%)	50 (36%)
	Some of the time	11 (8%)	6 (4%)
	Don’t live with a partner	81 (58%)	83 (59%)
**Main partner had other sex partners during past year**			
	Know or suspect	31 (22%)	45 (32%)
	Don’t suspect	64 (46%)	46 (33%)
	Don’t know	40 (29%)	46 (33%)
	No main partner	4 (3%)	1 (1%)
**Number of male sex partners in lifetime**			
	1–2	58 (42%)	65 (47%)
	3–4	56 (40%)	52 (37%)
	5–6	19 (14%)	18 (13%)
	≥ 7	6 (4%)	4 (3%)
**Any sex partners ever tested positive for HIV**			
	Yes	8 (6%)	6 (4%)
	No	103 (74%)	106 (76%)
	Don’t know	27 (19%)	27 (19%)
**Number of vaginal sex acts per week during past 4 weeks**			
	0	3 (2%)	5 (4%)
	1–2	66 (48%)	66 (48%)
	3–4	37 (27%)	37 (27%)
	5–6	11 (8%)	10 (7%)
	≥ 7	21 (15%)	20 (14%)
**Used male condoms with vaginal sex during past 4 weeks**			
	Never	27 (19%)	30 (22%)
	Sometimes	37 (26%)	37 (27%)
	Always	74 (53%)	71 (51%)
**Sex in exchange for money, food, drugs, or shelter during past year**		1 (1%)	3 (2%)
**Anal sex during past year**		4 (3%)	2 (1%)
**Thinks she might be at risk of getting HIV**		20 (16%)	27 (21%)

* Information on ethnicity was provided by 92.9% of participants.

** Coloured is a national ethnic classification used in South Africa that describes a person with mixed racial/ethnic origins.

Based on participants who provided information on their relationship status and sexual history at the screening visit, the majority were single (62%), and 22% were married (Table 2). All participants reported having a main sex partner at the time of their enrolment. In each treatment group, 6% of participants indicated that they had other sex partners. Approximately half of the participants (48% in each treatment group) reported one to two acts of vaginal intercourse per week over the previous 4 weeks, and just over 50% in both groups reported that they always used male condoms during vaginal intercourse over the same period. A small minority reported using female condoms (less than 2%). One woman in the dapivirine ring group and three women in the placebo ring group reported having intercourse in exchange for money, food, drugs, or shelter during the past year. Participant reports of anal intercourse during the past year were low in both treatment groups (3% in the dapivirine ring group and 1% in the placebo ring group).

When asked if their partner had other female partners in the past year, 40% of participants did not suspect this, 27% knew or suspected that their partners had other female partners, and 31% did not know (Table 2). Five percent of the women reported that they had a partner who had tested positive for HIV, 75% stated that they did not and 19% of the women did not know. Participants’ perception of risk for HIV acquisition was relatively low; 16% in the dapivirine ring group and 21% in the placebo ring thought that they might be at risk of HIV infection.

### Ring Exposure

The majority of enrolled participants reportedly used the vaginal rings between 78 and 84 days (over an expected ring use period of 84 days): 72.1% (101/140) of participants in the dapivirine ring group and 68.6% (96/140) of participants in the placebo ring group. The mean number of days of vaginal ring exposure was 83.3 (± 4.5) days for dapivirine ring users and 79.7 (± 15.9) days for placebo ring users. Only a small proportion of women (4.3% [6/140] of participants in the dapivirine ring group and 10.7% [15/140] of participants in the placebo ring group) reportedly used the rings less than 78 days.

### Safety Outcomes

#### Pelvic and Colposcopic Examinations

Post-baseline pelvic examination abnormalities were observed in 48 (34.3%) participants using dapivirine rings and 45 (32.1%) participants using placebo rings (RR 1.1; 95% CI: 0.8–1.5) (Table 3). The most commonly reported findings were abnormal vaginal discharge reported for 20 (14.3%) dapivirine and 21 (15.0%) placebo ring users, and intermenstrual bleeding reported for 17 (12.1%) dapivirine and 16 (11.4%) placebo ring users.

**Table 3:** Proportion of Participants Experiencing Safety Outcomes during the Trial. CI = Confidence interval; DAIDS = Division of AIDS; RR = Risk ratio; STI = Sexually transmitted infection. Baseline was defined as pre-enrolment (Visit 1, Week 0) for pelvic/speculum and colposcopy assessments, adverse events, vaginal pH and vaginal flora assessments, and as screening visit for STI testing and safety laboratory tests. Point estimates and asymptotic 95% CIs are presented for the risk ratio comparing the proportion of women diagnosed with event post-baseline between the dapivirine ring and placebo ring arms.

Safety Endpoint		Intent-to-treat Dapivirine Vaginal Ring(N = 140)	Intent-to-treat Placebo Vaginal Ring(N = 140)	RR (95% CI)
		n (%)	n (%)	
**Composite**				
	Baseline	123 (87.9%)	118 (84.3%)	
	Post-baseline	128 (91.4%)	131 (93.6%)	1.0 (0.9, 1.0)
**Post-baseline abnormal finding during pelvic/speculum examination**				
	Baseline	1 (0.7%)	3 (2.1%)	
	Post-baseline	48 (34.3%)	45 (32.1%)	1.1 (0.8, 1.5)
**Post-baseline abnormal finding during colposcopy examination**				
	Baseline	1 (0.7%)	2 (1.4%)	
	Post-baseline	23 (16.4%)	23 (16.4%)	1.0 (0.6, 1.7)
**Post-baseline diagnosis of STI**				
	Baseline	22 (15.7%)	27 (19.3%)	
	Post-baseline	29 (20.7%)	34 (24.3%)	0.9 (0.6, 1.3)
**Post-baseline DAIDS Grade 2+ laboratory test**				
	Baseline	0 (0.0%)	2 (1.4%)	
	Post-baseline	3 (2.1%)	7 (5.0%)	0.4 (0.1, 1.6)
**Post-baseline adverse event**				
	Baseline	0 (0.0%)	0 (0.0%)	
	Post-baseline	114 (81.4%)	121 (86.4%)	0.9 (0.9, 1.0)
**Post-baseline abnormal pH**				
	Baseline	67 (47.9%)	64 (45.7%)	
	Post-baseline	100 (71.4%)	99 (70.7%)	1.0 (0.9, 1.2)
**Post-baseline abnormal vaginal flora**				
	Baseline	35 (25.0%)	31 (22.1%)	
	Post-baseline	57 (40.7%)	62 (44.3%)	0.9 (0.7, 1.2)

In each ring group, 23 (16.4%) participants showed abnormalities on post-baseline colposcopic examinations. These findings mostly included petechiae and erythema, occurring in 8.6% and 5.0% of dapivirine and placebo ring users, respectively. Other findings were observed in less than 5% of participants per treatment group with most of the findings located on the cervix. Two events of petechiae and a vulvar laceration were ongoing in two dapivirine ring users; however, neither event was assessed by the Investigator as related to product use.

No clinically significant increase in pelvic and colposcopic findings relative to baseline was observed over time.

#### Adverse Events

The incidence of TEAEs was similar in dapivirine (114/140; 81.4%) and placebo ring users (121/140; 86.4%) (RR 0.9, 95% CI: 0.9; 1.0) (Table 3). The most commonly observed TEAE was metrorrhagia (reported as “intermenstrual bleeding” or “breakthrough bleeding”) and occurred in 26 (18.6%) dapivirine and 27 (19.3%) placebo ring users (Table 4). Most metrorrhagia cases (88.9% in dapivirine group versus 96.1% in placebo group) were assessed by the Investigator as mild in severity (Grade 1) and did not require treatment. Other frequently reported TEAEs (≥ 10% of participants using dapivirine rings) were gynaecological chlamydia infection, urinary tract infection, vaginal candidiasis, and upper respiratory tract infection (Table 4).

**Table 4:** Treatment Emergent Adverse Events with an Incidence of > 5% in either Treatment Group. DAIDS = Division of AIDS; MedDRA = Medical Dictionary for Regulatory Activities.

			Regardless of causality			Product-related events**		
MedDRA System Organ Class / Preferred term		Reported Severity (DAIDS Grade*)	Dapivirine Vaginal Ring(N = 140)	Placebo Vaginal Ring(N = 140)	All Participants(N = 280)	Dapivirine Vaginal Ring(N = 140)	Placebo Vaginal Ring(N = 140)	All Participants(N = 280)
			n (%)	n (%)	n (%)	n (%)	n (%)	n (%)
Number of participants with one or more events			114 (81.4%)	121 (86.4%)	235 (83.9%)	30 (21.4%)	31 (22.1%)	61 (21.8%)
**Infections and infestations**			97 (69.3%)	98 (70.0%)	195 (69.6%)	12 (8.5%)	8 (5.7%)	20 (14.3%)
	Gynaecological chlamydia infection	Grade 1, 2	22 (15.7%)	22 (15.7%)	44 (15.7%)			
	Urinary tract infection	Grade 1, 2	18 (12.9%)	14 (10.0%)	32 (11.4%)	3 (2.1%)	3 (2.1%)	6 (2.1%)
	Vaginal candidiasis	Grade 1, 2	20 (14.3%)	12 (8.6%)	32 (11.4%)	4 (2.9%)	0 (0.0%)	4 (1.4%)
	Upper respiratory tract infection	Grade 1, 2	15 (10.7%)	16 (11.4%)	31 (11.1%)			
	Vaginitis bacterial	Grade 1,2	10 (7.1%)	13 (9.3%)	23 (8.2%)	3 (2.1%)	2 (1.4%)	5 (1.8%)
	Asymptomatic bacteriuria	Grade 1	11 (7.9%)	7 (5.0%)	18 (6.4%)			
	Gonorrhoea	Grade 1, 2	7 (5.0%)	10 (7.1%)	17 (6.1%)			
	Urogenital trichomoniasis	Grade 1, 2	5 (3.6%)	8 (5.7%)	13 (4.6%)			
	Gastroenteritis	Grade 1, 2	1 (0.7%)	8 (5.7%)	9 (3.2%)			
	Vulvovaginal mycotic infection	Grade 1				2 (1.4%)	3 (2.1%)	5 (1.8%)
**Reproductive system and breast disorders**			51 (36.4%)	51 (36.4%)	102 (36.4%)	14(1.0%)	7 (0.5%)	21 (15%)
	Metrorrhagia	Grade 1, 2, 3	26 (18.6%)	27 (19.3%)	53 (18.9%)	9 (6.4%)	4 (2.9%)	13 (4.6%)
	Vaginal discharge	Grade 1, 2	10 (7.1%)	7 (5.0%)	17 (6.1%)			
	Vulvovaginal pruritus	Grade 1, 3	7 (5.0%)	6 (4.3%)	13 (4.6%)	5 (3.6%)	3 (2.1%)	8 (2.9%)
	Oligomenorrhoea	Grade 1	8 (5.7%)	2 (1.4%)	10 (3.6%)			
**Nervous system disorders**			10 (7.1%)	10 (7.1%)	20 (7.1%)			
	Headache	Grade 1, 2	7 (5.0%)	10 (7.1%)	17 (6.1%)			

* Severity DAIDS grade: Grade 1 (mild), Grade 2 (moderate), Grade 3 (severe), Grade 4 (potentially life-threatening).

** Product-related TEAEs included only possibly or probably related events. No TEAEs were considered as definitely related to ring use.

Assessments of causality were made by the Investigator and no TEAEs were considered definitely related to vaginal ring use. Probably or possibly related events were reported for 30 (21.4%) and 31 (22.1%) dapivirine and placebo ring users, respectively (Table 4). The most commonly observed product-related TEAE was metrorrhagia, reported for nine (6.4%) dapivirine and four (2.9%) placebo ring users. Of the 235 participants in the trial who experienced at least one TEAE, 94.5% (222/135) of participants reported TEAEs of mild (113/280; 40.4%) or moderate severity (109/280; 38.9%), with 12 participants experiencing events of severe (Grade 3) intensity. Five serious adverse events (SAEs), which included one death due to Grade 4 (life-threatening) hemopneumothorax as a result of physical assault in a placebo ring user, were reported during the trial. Grade 3 (severe) tonsillitis was experienced by a participant in the dapivirine ring group, and bronchiectasis, peritonsillar abscess, and a suicide attempt (all Grade 3 in severity) was each experienced by three participants in the placebo ring group. None of the SAEs were regarded as product-related.

#### Safety Laboratory Tests

No disconcerting laboratory abnormalities were observed between the two treatment groups (RR 0.4, 95% CI: 0.1; 1.6) (Table 3).

#### Sexually Transmitted Infections

A total of 29 (20.7%) dapivirine ring users and 34 (24.3%) placebo ring users showed post-baseline positive test results for chlamydia, gonorrhoea and trichomonas (RR 0.9, 95% CI: 0.6; 1.3) (Table 3). Chlamydia occurred in 22 (15.7%) participants in both ring groups. Eight (5.7%) participants using dapivirine rings and 10 (7.1%) participants using placebo rings tested positive for gonorrhoea, followed by trichomonas, observed in two (1.4%) dapivirine and five (3.6%) placebo ring users. None of the dapivirine or placebo ring users required temporary or permanent discontinuation of product use due to these infections. A small number of women (six dapivirine and three placebo ring users) who experienced post-baseline STIs were also reported with vaginal candidiasis (see section on adverse events). Five dapivirine ring users and two placebo ring users experienced chlamydia infection as well as vaginal candidiasis. One participant in the placebo ring group was reported with gonorrhoea infection and vaginal candidiasis, and one participant in the dapivirine ring group was reported with trichomonas and vaginal candidiasis.

#### Vaginal Flora and Vaginal pH

The number of participants diagnosed with abnormal vaginal flora post-baseline (Nugent score ≥ 7) was slightly less in the dapivirine ring group compared to the placebo group: 57 (40.7%) participants using dapivirine rings compared to 62 (44.3%) using placebo rings (RR 0.9, 95% CI: 0.7; 1.2) (Table 3). The mean total Nugent score for vaginal flora was 4.50 at pre-enrolment (Week 0) and 4.55 after 12 weeks of ring use for dapivirine ring users, and 4.41 and 4.63, respectively, for placebo ring users.

Participants who presented with abnormal vaginal pH values (< 3.5 or > 4.8) after enrolment showed a similar incidence between the treatment groups at ~70% (compared to ~46% at baseline) (RR 1.0, 95% CI: 0.9; 1.2) (Table 3). The overall mean pH ranged from 4.74 prior to enrolment to 4.77 at the end of the 12-week ring-use period, with minor fluctuations observed in both treatment groups during this period.

#### Other Safety Assessments

Two participants in the dapivirine ring group and four participants in the placebo group became pregnant during the trial. Three participants acquired HIV-1 infection in the placebo ring group. These women discontinued product use immediately after their HIV-positive status was confirmed and received counselling and referral for social services and other clinically indicated medical services according to country-specific guidelines.

#### Dapivirine Concentrations in Plasma and Ring Residual Levels

Dapivirine plasma concentrations among participants in the dapivirine ring group were similar at Week 4 and Week 12, with mean plasma concentrations of 293.4 pg/ml and 238.9 pg/ml, respectively. The individual values at Week 4 and Week 12 did not exceed 708.0 pg/ml and 688.1 pg/ml, respectively. Between-participant variability (%CV) in dapivirine plasma concentrations at ring removal was somewhat higher at Week 12 compared to Week 4 (60.7% versus 45.5%).

A total of 816 vaginal rings were returned for residual dapivirine content analysis. Results from the analysis indicated that approximately 4 mg of dapivirine was released from the rings over the 28-day use period. Mean residual amounts of dapivirine were similar for Weeks 4, 8 and 12, at 21.09 mg, 21.54 mg and 21.84 mg, respectively. The lower and upper quartiles were 20.4 to 21.9 mg, 20.4 to 22.9 mg, and 20.6 to 23.3 mg, respectively. The results should be considered in light of the fact that less than 20 used dapivirine rings were returned per visit per research centre.

**Fig 2:**
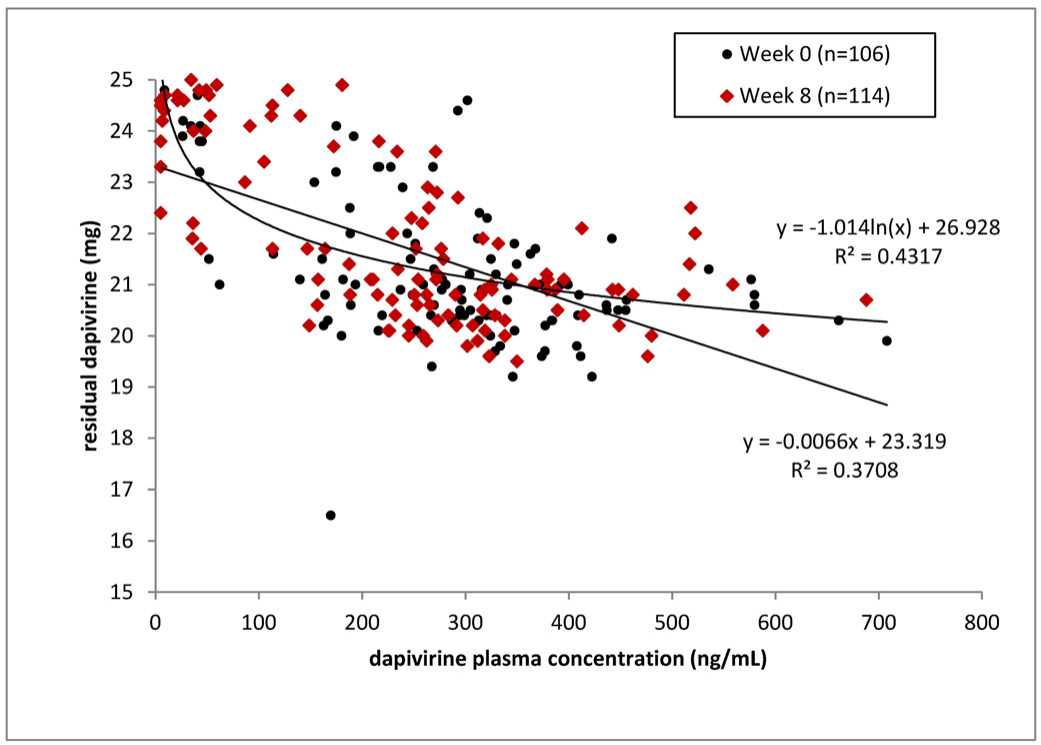
Residual Amount of Dapivirine (mg) versus Plasma Concentration of Dapivirine (pg/ml). The two lines reflect the linear and exponential relationships evaluated with corresponding regression coefficients (R^2^). Dapivirine residual levels were assessed for rings dispensed at Weeks 0, 4 and 8 (i.e. returned at Weeks 4, 8 and 12). Dapivirine plasma levels were assessed at Weeks 0, 4 and 12. Data points at Week 0 refer to ring removal of the first ring during Week 4 at which time blood sampling for plasma dapivirine analysis was conducted. Data points at Week 8 refer to ring removal of the third ring during Week 12 at which time blood sampling for plasma dapivirine analysis was conducted.

Regression analysis was performed to evaluate a potential linear and/or exponential relationship between residual levels of dapivirine and corresponding plasma concentrations. The exponential relationship appeared to fit the data slightly better; however, for both regression analyses this value was low (r^2^ = 0.37 and 0.43 for the linear and exponential relationship, respectively) (Fig 2). As shown in Fig 2, it appears that plasma concentrations below approximately 200 pg/ml were generally associated with above-average ring residual amounts, while the residual amounts appeared relatively constant (at levels between approximately 20 and 22 mg) for plasma concentrations above this value (200 pg/ml). No clear relationship was observed between the residual level of dapivirine and the dapivirine plasma concentration obtained at the corresponding pharmacokinetic visit (i.e., scheduled ring removal). Data should be interpreted with caution due to the limited amount of data. The number of data points at Week 0 and Week 8 was nearly the same (n = 106 at Week 0; n = 114 at Week 8) (Fig 2). Dapivirine residual levels were assessed for rings dispensed at Weeks 0, 4 and 8 (i.e. returned at Weeks 4, 8 and 12). Dapivirine plasma levels were assessed at Weeks 0, 4 and 12. In Fig 2, data points at Week 0 refer to ring removal of the first ring during Week 4 at which time blood sampling for plasma dapivirine analysis was conducted. Data points at Week 8 refer to ring removal of the third ring during Week 12 at which time blood sampling for plasma dapivirine analysis was conducted.

### Social and Behavioural Data

The acceptability data analysis for the ITT population included data from 277 (98.9%) of the 280 enrolled participants, and the adherence analysis included data from 278 (99.3%) participants. Enrolled participants not included in the ITT analyses for these endpoints were discontinued early from the trial and did not have any acceptability and adherence questionnaire data. Adherence data provided for each of the four scheduled follow-up visits were available for 261 (93%) participants, whilst 267 (95%) participants provided acceptability data for the final acceptability assessment at Week 12.

#### Overall Vaginal Ring Acceptability

Outcomes of vaginal ring acceptability data collected after 4 and 12 weeks of ring use are presented in Table 5. At Weeks 4 and 12, trial participants were asked how they felt about wearing the vaginal ring every day. The majority of women in each treatment group, and at both visits, reported that they were “usually comfortable” (96% of participants in both treatment groups at Week 4 and 97% of participants in both groups at Week 12). One percent of the women reported feeling “usually uncomfortable” (three placebo ring users at Week 4 and three dapivirine ring users at Week 12).

**Table 5:** Vaginal Ring Acceptability at Week 4 and Week 12.

		Week 4(n = 275)	Week 12(n = 267)
**Basic acceptability**	If thought she was at risk of HIV, willing to use ring if proven effective	265 (96%)	257 (96%)
**Ring experiences (previous 4 weeks)**	Ring easy to insert *(at last insertion)*	261 (95%)	243 (95%)
	Never aware of ring during daily activities	233 (85%)	237 (89%)
	Wearing ring daily was usually comfortable	265 (95%)	257 (97%)
	Never felt ring during sex	233 (87%)	242 (92%)
**Preferences for use regimen**	She prefers daily use versus non-daily use	264 (95%)	258 (97%)
	Prefers to wear during menses versus not wearing during menses	190 (69%)	182 (69%)
**Concerns about ring**	Concerned that ring may get lost or stuck in the body	76 (28%)	59 (22%)
	Concerned that ring may fall out	60 (20%)	42 (16%)
**Partner perceptions of ring (previous 4 weeks)**	Important that partner does not feel ring during sex	144 (53%)	132 (49%)
	Partner did not feel the ring during sex	165 (61%)	169 (63%)
	Partner felt the ring during sex, but it was not a problem	55 (20%)	60 (22%)
	Does not know if he felt the ring	42 (16%)	34 (13%)

Similarly, the majority of participants at both visits, and in both treatment groups (96% at Week 4; 97% at Week 12), indicated a willingness to wear the ring in the future if it were proven to be effective and they thought they were at risk of HIV infection.

#### Awareness, Preferences and Concerns about Vaginal Ring Use

A more comprehensive acceptability assessment also indicated high participant acceptability for ring use at both Weeks 4 and 12 (Table 5). In both treatment groups combined, 85% of women at Week 4 reported “never” being aware of ring use during their daily activities, 6% were aware of it “sometimes”, and 9% were aware of it “most of the time”. These latter proportions declined slightly by Week 12, such that 5% of participants reported being “sometimes” aware of the ring, 6% being aware of it “most of the time”, and 89% of participants “never” being aware of the ring. A similar pattern was observed for awareness of the ring during intercourse. Overall, there were no substantial differences by treatment group, with 87% of participants in both groups reporting never feeling the ring during intercourse at Week 4, and 92% reporting the same at Week 12.

Only a minority of participants expressed a hypothetical preference for not wearing the vaginal ring every day (4/274 [1%] at Week 4, and 6/267 [2%] at Week 12). The majority of the remaining participants preferred to wear it daily (96% and 97% in both treatment groups at Weeks 4 and 12, respectively), while a small minority had no preference.

Participants were asked about their preferences with regard to ring use during menses. At Week 12, women in both the dapivirine and placebo groups combined were either amenorrheaic and had no preference (18%), had normal menses but no preference (4%), or preferred to continue wearing the ring during menses (69%). Less than 10% of participants at Weeks 4 and 12 in both treatment groups expressed a preference to not wear the ring during menses.

Around one-quarter of trial participants in both treatment groups combined held the concern that the ring might fall out (22%) or get lost inside their body (28%) at Week 4. These concerns dissipated slightly, such that only 16% and 22% reported the respective concerns by Week 12.

#### Perceptions of Vaginal Ring Acceptability to Male Partners

Trial participants were also asked about the importance of their male partner not feeling the vaginal ring during intercourse, and to what extent this was experienced. Female participants were evenly divided with regard to how important they perceived this issue: 53% of women in both treatment groups felt it was important at Week 4 (versus 47% who felt that it was not important) and this proportion declined somewhat to 49% stating its importance at Week 12 (and 50% not; one participant did not have a main partner at Week 12). Just over 60% of the participants in both ring groups reported that their male partners did not feel the ring at Week 12. A further 22% reported that their partner felt the ring during intercourse, but that it was not a problem. Only a small number (8/270 [3%] at Week 4 and 3/267 [1%] at Week 12) reported that her male partner felt the ring and that it might be (n = 7), or definitely was (n = 4) a problem for her to continue ring use.

#### Vaginal Ring Adherence Outcomes

**Fig 3:**
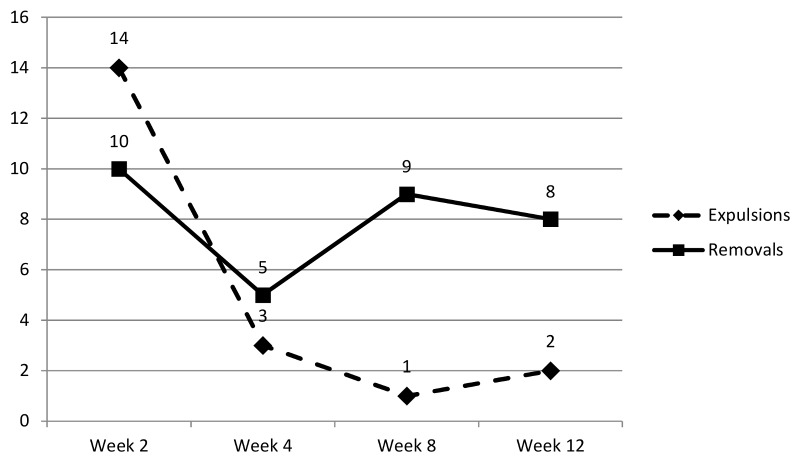
Participant Self-Reported Ring Expulsions and Removals by Trial Visit (Week).

Of the 280 participants enrolled, 261 (93%) had adherence data available for each of the four scheduled follow-up visits, and were included in the assessment of cumulative adherence. Self-reported adherence trends are summarized in Fig 3. Overall, cumulative adherence was high and evenly distributed by treatment group. Ninety-two percent (92%) of participants reported that they never had the ring out for one full day or more for the entire trial period.

#### Visit-specific Adherence and Participant Use Experiences

At each follow-up visit, a high proportion of women in both ring groups reported that they never had the ring out since their last visit. This ranged from 91% of participants at Week 2 to 97% at Week 4 to 96% each at Weeks 8 and 12.

A total of 24 (24/273; 9%), 8 (8/275; 3%), 10 (10/267; 4%) and 11 (11/267; 4%) participants at Weeks 2, 4, 8, and 12, respectively, reported the ring out of their vagina, i.e. rings expelled or removed. The median number of times that the ring was out of the vagina among these women was one time during each visit interval (range: 1–5 times at Week 2, 1–3 times at Weeks 4 and 8, and 1–22 at Week 12). A total of 20 ring expulsions and 32 ring removals were reported. Most ring expulsions were reported during the first two weeks of trial participation, with 14 expulsions being reported at Week 2, three expulsions at Week 4, one expulsion at Week 8 and two expulsions at Week 12. The most frequent event associated with vaginal ring expulsion was defecation and/or urination, which was associated with 70% of expulsions.

Reported ring removals followed a different trend, with participants reporting ten ring removals at Week 2, five removals at Week 4, nine removals at Week 8 and eight removals at Week 12. The most common reason for ring removal was to clean it (n = 11), followed by participant concerns about the ring (n = 5), discomfort/pain being experienced (n = 5), and ring removal on request of the male partner (n = 4). Only a small number of participants (n = 2) across all visits described menses as the reason for ring expulsion (n = 2 at Week 2) or ring removal (n = 1 at Week 2; n = 1 at Week 12).

At Weeks 2, 4 and 8, between 57% and 71% of the participants who reported the ring expelled or removed, rinsed and re-inserted it (data not shown). At Week 12, only three out of 11 participants (27%) who reported ring expulsions or removals in the previous visit interval had re-inserted their rings. Women were asked about the longest period of time that the ring was ever out, and for most women this tended to be for a period of hours, rather than days. As a measure of risk exposure, women were asked whether they had intercourse during the time that the ring was out of the vagina. Although the majority did not engage in intercourse without the ring, two to four women at each visit reported this behaviour, which ranged from 17% (Week 4) to 36% (Week 12) of those reporting the ring out of the vagina (data not shown).

## Discussion

The dapivirine vaginal ring was generally perceived as safe and well-tolerated for up to 12 weeks of continuous use, and no clinical safety concerns were identified for any of the safety variables assessed during the trial. Adverse events occurred overall in a similar frequency between dapivirine and placebo ring users, and the profile and incidence of the most frequently reported events were consistent with those observed during a 3-month observational safety arm of an earlier IPM trial (IPM 011, ClinicalTrials.gov No NCT00469170) [14], as well as those reported in the CAPRISA 004 tenofovir gel trial [15] and the HPTN 035 trial of BufferGel and Pro2000 gel [16]. The most commonly reported product-related event was metrorrhagia, experienced by 13 participants of whom three dapivirine ring users and one placebo ring user had a history of intermenstrual bleeding. Although cases of product-related metrorrhagia were reported during the trial in twice as many participants using dapivirine than placebo rings, the incidence was low and consistent with the incidence observed in the 3-month observational safety arm of the IPM 011 trial when no device or investigational product was administered [14].

Differences in the observed incidences of gynaecological chlamydia infection, urinary tract infection, and vaginal candidiasis between dapivirine and placebo ring users were considered not clinically significant. Five participants in the dapivirine ring group experienced a recurring case of vaginal candidiasis for which ring use in two of the women was temporarily interrupted by the Investigator; the two events lasted 2 and 4 days, respectively. Participants who experienced vaginal candidiasis during the trial showed no difference in their self-reported adherence to ring use.

The trial was exploratory in nature with the primary objective to evaluate the safety of dapivirine vaginal ring. The degree to which the safety outcomes of this trial could be influenced by the research centres was evaluated by examining the interaction of treatment and research center using the Cochran Mantel Haenszel (CMH) test with the null hypothesis that the treatment effect would be the same across all research centers at a significance level of 0.05 [17]. For all primary safety endpoints where a risk ratio was calculated, a statistically non-significant result was obtained, indicating a lack of evidence of a treatment by research centre interaction.

In general, plasma concentrations of dapivirine were low; the individual values at Weeks 4 and 12 did not exceed 708.0 pg/ml and 688.1 pg/ml, respectively, which were well below the plasma dapivirine concentration observed at the maximum tolerated dose for oral treatment (mean C_max_ 2286 ng/ml, Investigator’s Brochure TMC vaginal microbicide, Tibotec 2003). In two earlier dapivirine ring pharmacokinetic trials, dapivirine concentrations measured in vaginal fluids and cervical tissue were well above the *in vitro* IC_99_ for provirus integration into cervical tissue (3.3 ng/ml) following challenge with HIV-1_BaL_ [8, 9]. Only approximately 4 mg of the drug load of 25 mg dapivirine was released over 28 days. The observed cases of non-adherence were not clearly reflected in the obtained dapivirine plasma concentrations and dapivirine ring residual levels; however, in cases where the ring was worn shorter than the planned 28-day period or removed for multiple days, the obtained residual levels tended to be towards the higher end of the observed range of values. Plasma dapivirine concentrations and dapivirine residual levels are currently explored in a pharmacokinetic model to evaluate their possible role as objective adherence indicators in large-scale Phase III clinical trials.

Self-reported adherence to daily use of the dapivirine or placebo vaginal ring over the 12-week trial period was very high and the vaginal ring was acceptable to the majority of participants who also reported that they would be willing to use it in the future if it were proven effective and they thought they were at risk for HIV infection. These results correspond to the high self-reported acceptability and participant adherence that was observed in the 3-month observational placebo ring trial (IPM 011) conducted in South Africa and Tanzania [18, 19]. Results from earlier vaginal gel microbicide trials have shown that adherence to product use are often over reported by users during clinical trials and measuring user adhertableence in microbicide trials remains a difficult aspect which is often limited to user self-report and biological markers [3, 20]. The relationship between participant adherence to vaginal ring use and plasma/residual levels of dapivirine in returned rings will be investigated in IPM’s ongoing Phase III clinical trial.

The vaginal rings appeared easy to use and the observed rate of ring expulsions and removals was low, with ring expulsions occurring more frequently in the initial ring use period. The most frequent activities associated with ring expulsion were urination and/or defecation. While the frequency of ring expulsion decreased over time, ring removals tended to increase over time. The most common reason women reported for removing the ring was to clean it, even though research staff had advised against it. In future trials, vaginal ring use counselling could be strengthened to provide emphasis and participant support for continuous ring use by explaining the lack of necessity to remove for cleaning. Another important finding that should inform future ring-use counselling is that almost a quarter of the women reported that their male partners could feel the vaginal ring during intercourse, although most noted this was not a problem.

## Conclusion

Based on its favourable safety and acceptability profile, the dapivirine vaginal ring is considered a safe, easy and convenient method for use by women in the African setting. The dapivirine ring is further being evaluated in a Phase III program among sub-Saharan African women for extended safety, acceptability and efficacy.

## Supporting Information

S1 Text Consort Checklist. (DOC)

S2 Text Study Protocol. (PDF)
