# Supplementary material for: Safety, Acceptability and Adherence of Dapivirine Vaginal Ring in a Microbicide Clinical Trial Conducted in Multiple Countries in Sub-Saharan Africa
Source: PLoS One. 2016 Mar 10;11(3):e0147743. doi: 10.1371/journal.pone.0147743 (PMC4786336; doi:10.1371/journal.pone.0147743)
Supplement: S2 Text — (PDF) [file pone.0147743.s002.pdf]

IPM 015 PROTOCOL
Version 2.0, Amendment 3.0
South African Sites

A DOUBLE-BLIND, RANDOMIZED, PLACEBO-CONTROLLED PHASE I/II STUDY TO EVALUATE THE SAFETY OF AN INTRAVAGINAL MATRIX RING WITH DAPIVIRINE IN HEALTHY HIV-NEGATIVE WOMEN

SPONSOR:
International Partnership for Microbicides
8401 Colesville Road, Suite 200
Silver Spring, MD 20910 U.S.A.

PROTOCOL VERSION 2.0, Amendment 3.0: South African Sites
21 December 2009

Restricted * Confidential – Limited Access

SPONSOR CONTACT INFORMATION

**Sponsor Contact**	Dr. Annaléne Nel, MBChB, PhD
Office Phone:	+27 21 860 2300
Office Fax:	+27 21 860 2308
Office E-mail:	anel@ipm-microbicides.org
Physical Address:	International Partnership for Microbicides<br>JBMH Building<br>121 Main Street<br>Paarl, 7646<br>South Africa
Mailing Address:	International Partnership for Microbicides<br>P.O. Box 3460<br>Paarl, 7646<br>South Africa

**Sponsor Clinical Safety Physician**	Dr. Mercy Kamupira, MBChB
Office Phone:	+27 21 860 2300
Office Fax:	+27 21 860 2308
Office E-mail:	mkamupira@ipm-microbicides.org.za
Physical Address:	International Partnership for Microbicides<br>JBMH Building<br>121 Main Street<br>Paarl, 7646<br>South Africa
Mailing Address:	International Partnership for Microbicides<br>P.O. Box 3460<br>Paarl, 7646<br>South Africa

SIGNATURE PAGE

IPM 015

A DOUBLE-BLIND, RANDOMIZED, PLACEBO-CONTROLLED PHASE I/II STUDY TO EVALUATE THE SAFETY OF AN INTRAVAGINAL MATRIX RING WITH DAPIVIRINE IN HEALTHY HIV-NEGATIVE WOMEN

I have read this protocol and appendices and agree to conduct the study as stipulated and in compliance with the principles of the World Medical Association Declaration of Helsinki, the International Conference on Harmonisation (ICH) Good Clinical Practice (GCP) guidelines, and all applicable regulations and guidelines.

__________________ ______________________________
Investigator Signature Date

__________________ ______________________________
Investigator Name (Printed) Investigative Research Centre Name

On behalf of the International Partnership for Microbicides, I confirm that the sponsor will comply with all obligations as detailed in all applicable regulations and guidelines. I will ensure that the investigator is informed of all relevant information that becomes available during the conduct of this study.

____________________________________ ______________________________
Chief Medical Officer Signature Date

Annaléne Nel, MBChB, PhD
Chief Medical Officer Name (Printed)

TABLE OF CONTENTS

Page

SPONSOR: 1
PROTOCOL SYNOPSIS 10
1.0 INTRODUCTION 15
1.1 Background 15
1.2 Dapivirine Intravaginal Ring 15
1.2.1 Dapivirine 15
1.2.2 Nonclinical Research 16
1.2.3 Clinical Research 17
1.2.4 Formulation of silicone elastomer vaginal matrix ring containing 25mg of dapivirine 18
1.3 Rationale for Protocol IPM 015 18
3.0 OVERALL STUDY DESIGN 19
3.1 Study Design 19
3.2 Study Duration 20
3.3 Study Population 20
3.3.1 Inclusion Criteria 21
3.3.2 Exclusion Criteria 22
4.0 STUDY VISITS AND PROCEDURES 23
4.1 Screening Visit 23
4.2 Visit 1 = ENROLMENT VISIT 24
4.2.1 Pre-Enrolment Procedures 24
4.2.2 Enrolment Procedures 25
4.3 Visit 2 = Week 2 Post Enrolment 26
4.4 Visit 3 = Week 4 Post Enrolment 27
4.5 Visit 4 = Week 8 Post Enrolment 28
4.6 Visit 5 = Week 12 Post Enrolment 30
4.7 Visit 6 = Week 16 Post Enrolment and LAST STUDY VISIT 31
4.8 Unscheduled Visits 32
4.9 Missed & Late Visits 32
4.10 Early Discontinuation Visit 32
4.11 Premature Discontinuation of the Study 33
5.0 STUDY PROCEDURE DETAILS 33
5.1 INFORMED CONSENT 33
5.2 HIV Counselling 34
5.2.1 HIV Pre- and Post-Test Counselling 34
5.2.2 HIV/STI Risk Reduction Counselling 35
5.3 HIV Testing & Management 35

TABLE OF CONTENTS (Continued)

Page

5.4 Contraceptive Counselling.. 38
5.5 Pregnancy Testing and Management.. 38
5.6 Medical History and Physical Examination.. 38
5.7 Pelvic/Speculum Examination 38
5.8 Intravaginal Ring Placement Check 39
5.9 Colposcopy………………………………………………………………………..39
5.10 Laboratory Testing... 40
5.11 STI Testing….. 40
5.12 Vaginal Flora and Vaginal pH Testing 41
5.13 Pap Smear…… 41
5.14 Intravaginal Ring Insertion & Removal... 41
5.15 Intravaginal Ring Adherence Counselling 42
5.16 Assessments… 42
5.16.1 Adherence Assessments..42
5.16.2 Acceptability Assessments..42
5.16.3 Behavioural Assessments..42
5.16.4 Diary Card 42
5.17 Dapivirine Levels 42
5.18 Method of Treatment Assignment 42
5.19 Reimbursement.. 43
5.20 Study Operations Manual... 43
6.0 INVESTIGATIONAL PRODUCT………………………………………………43
6.1 Investigational Product Composition... 43
6.2 Packaging and Labelling.. 44
6.3 Randomization... 44
6.4 Blinding and Unblinding 44
6.5 Investigational Product Storage... 45
6.6 Investigational Product Administration 45
6.7 Investigational Product Expulsion or Loss... 45
6.8 Investigational Product Accountability... 45
6.9 Concomitant Medications & Products.. 46
7.0 ADVERSE EVENTS 46
7.1 Definition…… 46
7.2 Assessment of Adverse Event Severity 47
7.3 Relationship to Investigational Product.. 47
7.4 Serious Adverse Events... 48
7.4.1 Serious Adverse Event Definition 48

TABLE OF CONTENTS (Continued)

Page

7.4.2 Serious Adverse Event Contact Information....................................49
7.4.3 Sponsor Notification of SAEs to Regulatory Agencies 49
7.4.4 Research centre Notification of SAEs to Local Ethics Committee or Local Health or Regulatory Authorities 50
7.5 Immediately Reportable Events 50
7.6 Safety Monitoring 50
8.0 DATA MANAGEMENT 50
8.1 Data Handling at Study Research centres 50
8.2 Source Data Verification 51
9.0 STATISTICAL METHODS 51
9.1 General Design 51
9.2 Endpoints and Assessments 51
9.2.1 The primary endpoints are: 51
9.2.2 The primary endpoints will be assessed through: 51
9.2.3 The secondary endpoints are: 52
9.2.4 The secondary endpoints will be assessed by: 52
9.2.5 The exploratory objectives will be addressed as follows: 52
9.2.6 The exploratory objectives will be assessed by: 52
9.3 Sample Size…. 52
9.4 Analyses…….. 53
9.4.1 Analysis of Safety 53
9.4.2 Analysis of Acceptability...54
9.4.3 Analysis of Adherence 54
9.5 Interim Analysis 55
9.6 Handling of Missing Data and Dropouts 55
10.0 INVESTIGATOR REQUIREMENTS 55
10.1 Study Initiation 55
10.2 Institutional Review Board or Independent Ethics Committee Approval 56
10.3 Study Monitoring and Audits 56
10.4 Case Report Forms 56
10.5 Disclosure of Data 57
10.6 Record Retention 57
11.0 ETHICAL CONSIDERATIONS 57
11.1 Ethical Review 57
11.2 Social Harms. ....……..58
12.0 PUBLICATION 58
13.0 REFERENCES 59
APPENDIX A: SCHEDULE OF CLINICAL PROCEDURES ....…60

TABLE OF CONTENTS (Continued)

Page

APPENDIX B: INTRAVAGINAL RING INSERTION & REMOVAL INSTRUCTIONS……………………………………………………………………...60

APPENDIX C: SAMPLE SCREENING CONSENT FORM TEMPLATE 64

APPENDIX D SAMPLE STUDY CONSENT FORM TEMPLATE………………………69

ABBREVIATIONS

AE	Adverse Event
AIDS	Acquired Immunodeficiency Syndrome
AIDS-defining Illness	A condition, such as an opportunistic infection, that is included in the 1993 Revised Classification System for HIV Infection and Expanded Surveillance Case Definition for AIDS among Adolescents and Adults
ALP	Alkaline phosphatase
ALT	Alanine aminotransferase
ARV	Antiretroviral
AST	Aspartate aminotransferase
AUC	Area under plasma concentration-time curve
BUN	Blood urea nitrogen
BV	Bacterial vaginosis
CDC	U.S. Centers for Disease Control and Prevention
CONRAD	Contraceptive Research & Development
CRF	Case Report Form
CSP	Clinical Safety Physician
DAIDS	Division of Acquired Immunodeficiency Syndrome
DAPY	Di-aminopyrimidine
EIA	Enzyme Immunoassay
FBC	Full Blood Count
FDA	Food & Drug Administration (U.S.)
GCP	Good Clinical Practice
GGT	Gamma glutamyl transferase
HIV-1	Human Immunodeficiency Virus-1
HSV	Herpes Simplex Virus
IB	Investigator’s Brochure
ICF	Informed Consent Form
ICH	International Conference on Harmonisation
IEC	Independent Ethics Committee
IND	Investigational New Drug
IPM	International Partnership for Microbicides
IRB	Institutional Review Board
IRE	Immediately Reportable Event
IUD	Intrauterine Device
IVRS	Interactive Voice Response System
IVR	Intravaginal Ring
MTD	Maximum Tolerated Dose
NNRTI	Non-nucleoside Reverse Transcriptase Inhibitor
NRTI	Nucleoside Reverse Transcriptase Inhibitor
PCR	Polymerase Chain Reaction
PID	Participant Identification number
PMTCT	Prevention of Mother-to-Child Transmission of HIV
R&D	Research and Development
RNA	Ribonucleic Acid
RPR	Rapid Plasma Reagin
SAE	Serious Adverse Event
SCR-ID	Screening Identification Number
SEC	Safety Evaluation Committee
SGOT	Serum Glutamic Oxaloacetic Transaminase
SGPT	Serum Glutamic Pyruvic Transaminase,
SIV	Site Initiation Visit

STI	Sexually Transmitted Infection
TEAE	Treatment Emergent Adverse Event
US	United States
WHO	World Health Organization

PROTOCOL SYNOPSIS

IPM 015

A DOUBLE-BLIND, RANDOMIZED, PLACEBO-CONTROLLED PHASE I/II STUDY TO EVALUATE THE SAFETY OF AN INTRAVAGINAL MATRIX RING WITH DAPIVIRINE IN HEALTHY HIV-NEGATIVE WOMEN

BACKGROUND:

To date, candidate vaginal microbicides have been formulated predominantly as gels. Multiple safety and efficacy trials with various microbicides have been completed or are currently underway, most of which evaluate microbicides in gel formulation delivered via a single-use vaginal applicator used prior to coitus. In order for a microbicide to be most effective, it is essential that it is used correctly. Therefore, it is important that a microbicide is acceptable to users, and it is likely that products that can be applied less frequently will be more acceptable and will achieve better user-compliance. Intravaginal rings that need only be replaced every 28 days may therefore have benefits over dosage forms that need to be used more frequently.

19 phase I and phase I/II clinical studies have evaluated the safety of dapivirine in intravaginal rings, gels and oral formulation. These clinical studies support the favourable safety profile and tolerability of dapivirine intravaginal ring. IPM 015 has been developed to assess the safety of a silicone elastomer intravaginal matrix ring containing 25mg of dapivirine inserted once every 28 days over a 12-week period among healthy, sexually active, HIV-negative women as compared with a placebo intravaginal ring.

OBJECTIVES:

The primary objective is:

- To assess and compare the safety of a silicone elastomer intravaginal matrix ring containing 25 mg of dapivirine and a placebo intravaginal ring inserted once every 28 days over a 12 week period among healthy, sexually active, HIV-negative women.

The secondary objectives are:

- To assess the acceptability of an intravaginal ring inserted once every 28 days over a 12-week period among healthy, sexually active, HIV-negative women.
- To assess women’s adherence to the use of an intravaginal ring inserted once every 28 days over a 12-week period.

The exploratory objectives are:

- To assess sexual behaviours and possible factors influencing adherence to the use of an intravaginal ring inserted once every 28 days over a 12-week treatment period.

- To explore potential methods that may be used for adherence determination in Phase III studies.

ENDPOINTS and ASSESSMENTS:

The primary endpoints are:
- The proportion of women with cervico-vaginal mucosal abnormalities (as defined in the WHO/CONRAD manual) visible by naked eye examination and/or colposcopy.
- The proportion of women with at least one adverse event during the 16-week study period.
- The proportion of women with any laboratory abnormalities on haematology, liver function, and renal function.
- The proportion of women with positive diagnostic tests for trichomonas, gonorrhoea, and chlamydia.
- The proportion of women with abnormal vaginal flora and / or pH at any point during the study.

The primary endpoints will be assessed through:
- Gynaecological assessments, including pelvic/speculum exam and colposcopy, and laboratory STI testing.
- Safety laboratory blood tests.
- Adverse event/serious adverse event reports.
- Vaginal flora analysis and vaginal pH measurement.

The secondary endpoints are:
- The proportion of women who find the use of the intravaginal ring acceptable.
- The proportion of women who are adherent to the use of the intravaginal ring inserted once every 28 days over the full 12-week period.

The secondary endpoints will be assessed by:
- Questionnaires regarding sexual behaviour, acceptability and adherence to the use of an intravaginal ring inserted once every 28 days.
- Self-reported diary of intravaginal ring use.
- Clinician assessment of the intravaginal ring in situ at each study visit.

The exploratory objectives will be addressed as follows:

Study data, including demographic characteristics, questionnaires on acceptability and adherence, questionnaires on sexual behaviour, and other self reported information including diary cards will be used in exploratory models as possible predictors of adherence and non-adherence to 28 day intravaginal ring use. Other exploratory models will investigate the degree of agreement between levels of dapivirine and/or residual levels of dapivirine in

returned rings and participant self reports of adherence to develop potential methods for monitoring adherence in larger studies.

The following assessments will be used in addressing the exploratory objectives:

- Questionnaire concerning factors influencing adherence and non-adherence to 28 day intravaginal ring use.
- Questionnaires regarding sexual behaviour, acceptability and adherence to the use of an intravaginal ring inserted once every 28 days.
- Self-reported diary of intravaginal ring use, sexual behaviour and condom use.
- Plasma dapivirine levels obtained at predetermined time points during the 12-week treatment period.
- Residual dapivirine levels measured in the returned used or unused rings.

DESIGN: IPM 015 is a double-blind, randomized, placebo-controlled Phase I/II safety study conducted at up to 17 research centres in Kenya, Malawi, Rwanda, South Africa, Tanzania and Zambia among approximately 280 healthy, sexually active, HIV-negative women to assess the safety of a silicone elastomer intravaginal matrix ring containing 25 mg of dapivirine. Upon enrolment at each research centre, participants will be randomly assigned in a 1:1 ratio to one of two study arms:

Arm 1: A silicone elastomer intravaginal matrix ring containing 25 mg of dapivirine to be inserted every 28 days for a 12 week period.
Arm 2: A placebo intravaginal ring containing no dapivirine to be inserted once every 28 days for a 12 week period.

Participants will be allowed a screening period of up to 28 days prior to enrolment. Once enrolled, both groups will participate in the study for a period of 16 weeks, with a 12 week treatment period and a follow-up visit 4 weeks post ring discontinuation.

STUDY POPULATION: Healthy, sexually active, HIV-negative women 18-40 years of age who understand the study and can provide informed consent.

SAMPLE SIZE: Approximately 280 women (~15 – 40 per research centre) will be enrolled with competitive enrolment.

REGIMEN: Potential participants who consent will be invited to screen for the study. All women who consent to participate in the study, meet specified eligibility criteria, have normal findings based upon a physical and pelvic/speculum examination with colposcopy, and have negative pregnancy and HIV tests, will be invited to enrol in

the study. Eligible women will be randomly assigned in a 1:1 ratio to one of two study arms.

Rings containing dapivirine or placebo rings containing no dapivirine will be inserted at 28 day intervals for 3 months (weeks 0, 4, and 8). Not including screening, all participants will have a total of 6 clinic visits: enrolment, week 0 (visit 1 first ring insertion); week 2 (visit 2, follow-up); week 4 (visit 3, first ring removed, second ring inserted); week 8 (visit 4, second ring removed, third ring inserted); week 12 (visit 5, third ring removed); and a follow-up visit at week 16 (visit 6, 4 weeks post-ring removal).

After initial ring insertion, women will remain in the clinic under observation for 30 minutes to assess for immediate reactions. At enrolment and at each study visit, all participants will undergo a pelvic/speculum examination; pre- and post-test HIV counselling; HIV/STI risk reduction counselling including condom dispensing and intravaginal ring adherence counselling will be done at weeks 0 -8 (visits 1 – 4), (please refer to section 5.15); HIV and pregnancy testing; and collection of locator, menses, and concomitant medication information. Contraceptive counselling will be performed at all visits except week 16 (visit 6). HIV PCR testing will be performed at week 0 (visit 1/enrolment) and at week 16 (visit 6), with an additional sample taken at week 12 (visit 5) to be tested only if the participant tests HIV positive at week 16 (visit 6). Colposcopy will be performed at week 0 (visit 1, baseline examination) and at weeks 4, 8, and 12 (visits 3, 4 & 5) following each ring removal. STI testing and safety laboratory testing will be performed at screening and at weeks 4, 8, and 12 (visits 3, 4 & 5). Behavioural assessments will be conducted at every scheduled visit, including sexual behaviour, intravaginal practices and condom use at weeks 0, 4, 8, 12, and 16 (visits 1, 3, 4, 5 & 6). Plasma dapivirine levels will be obtained at weeks 0, 4 and 12 (visits 1, 3 & 5). Residual dapivirine levels will be measured in the rings returned used or unused at weeks 4, 8 &12 (visits 3, 4 & 5). Acceptability assessments will be administered at weeks 0, 4 and 12 (visits 1, 3 & 5) and Adherence assessments will be administered at weeks 2, 4, 8 and 12 (visits 2, 3, 4 & 5). Participants will be provided with a diary card at screening and it will be reviewed at weeks 0 - 12 (visits 1 – 5). To assess the effect of the intravaginal ring on vaginal flora and vaginal pH, specimens will be taken at enrolment prior to initial ring insertion, and at weeks 4, 8 and 12 (visits 3, 4 & 5) after ring removal. Adverse events will be assessed at all visits post randomization.

STUDY DURATION: The maximum allowable time between screening and enrolment per participant is 28 days. Following enrolment into the study, each participant will be followed for a total of 12 weeks during continuous ring use, and 4 weeks post ring removal. It is anticipated that full enrolment per research centre will be completed in 24 weeks for a total of up to 40 weeks study duration.

STATISTICAL ANALYSIS:

The analysis of the primary endpoints will focus on safety assessments, using the intent-to-treat population, and comparing safety parameters in the women who are using the dapivirine-containing intravaginal matrix ring to women who are using the placebo intravaginal ring (IVR). Exploratory analyses using a per-protocol population or subgroups of particular interest may also be performed.

The analysis of the secondary and exploratory endpoints will focus on assessment of acceptability of the intravaginal ring, adherence to the use of the intravaginal ring, as well as investigation of possible factors influencing adherence of women participating in the study. Data for IVR acceptability, sexual behaviour, condom use and IVR use will be collected by self-report through interviewer-administered assessments and a participant diary. Additional adherence data will be collected by clinician assessment of IVR in situ at each study visit and dapivirine concentrations in plasma and in returned intravaginal rings at predetermined time points throughout the study. Appropriate statistical analyses will be performed.

An interim analysis of the safety assessment data will be conducted after approximately 70 participants have completed 12 weeks of ring use.

1.0 INTRODUCTION

1.1 Background

The AIDS epidemic continues to exact a devastating toll on the health, economic and political infrastructure, and social fabric of communities worldwide. During 2007, almost 2.5 million people became newly infected with HIV bringing the total number of people living with HIV to an estimated 33.2 million. In the same year almost 2.1 million died from AIDS, raising the global death toll to over 25 million since the first cases of AIDS were identified in 1981 (1). Over 95 percent of new infections are occurring in developing countries where increasing numbers of new HIV infections threaten the sustainability of expanded access to HIV treatment. Developing safe and effective HIV prevention technologies that can be made easily accessible in developing countries is thus an urgent public health priority.

Epidemiologic data published in the latest UNAIDS report show that women and girls bear a severe and increasingly heavy burden in the HIV epidemic. In Eastern Europe and Central Asia, an estimated 26% of adults living with HIV in 2007 were women aged 15 years and older, compared with 23% in 2001. In sub-Saharan Africa, women comprised 61% of HIV reactive adults, and among young people (aged 15-24 years) the ratio of infection had risen to three women for every infected man (1).

Unprotected heterosexual intercourse is currently the leading mode of HIV infection among females (2). Correct and consistent use of latex condoms is one proven method of preventing HIV transmission (3); however, condoms are widely regarded as inadequate prevention options for women if they are unable to negotiate condom use with their partners for fear of abuse or accusations of infidelity (4-6). Additionally, women who have sex with men in exchange for gifts or money may be reluctant to use condoms if the men are willing to pay more for sex without a condom. The female condom has been marketed as an alternative barrier method, but this device is relatively costly and requires a certain level of skill, as well as acceptance by the male partner (6). Developing HIV prevention options that women can use with or without their partner’s knowledge is a pressing global concern given the rapidly growing HIV infection rate among women and the absence of an effective vaccine. Topical microbicides that can be self-administered to the vagina are one such promising alternative.

1.2 Dapivirine Intravaginal Ring

1.2.1 Dapivirine

Dapivirine is a substituted di-aminopyrimidine (DAPY) derivative and one of a new generation of “tight binding” non-nucleoside reverse transcriptase inhibitors (NNRTIs). Dapivirine is a promising candidate for a topical microbicide because of its highly potent activity against HIV-1 and its favourable safety profile. *In vitro* tests have shown that dapivirine has limited efficacy against HIV-2 and no efficacy against common sexually transmitted infections. Dapivirine is therefore not intended to have activity against HIV-2 or other sexually transmitted diseases.

Dapivirine was originally developed by Tibotec Pharmaceuticals Ltd., a subsidiary of Johnson & Johnson. In March 2004, the International Partnership for Microbicides (IPM) entered into an agreement with Tibotec Pharmaceuticals Ltd. to develop dapivirine for the topical prevention of infection with HIV-1.

Dapivirine is being developed as a microbicide in products intended for use once daily (gels and films) and in an intravaginal ring that provides sustained delivery of the microbicide for 28 days. The safety and tolerability of dapivirine has been evaluated by IPM and Tibotec Pharmaceuticals in both animal and human studies via the oral and vaginal routes. Detailed information on dapivirine is available in the Investigator's Brochure (IB (7)).

1.2.2 Nonclinical Research

The potential of dapivirine as a microbicide for prevention of sexual transmission of HIV has been assessed and confirmed in different in vitro, ex vivo and in vivo models:

- The activity of dapivirine against wild-type HIV-1, African isolates of HIV-1 (including subtype C virus), and a panel of NNRTI-resistant viruses has been established using *in vitro* models with EC_{50} values ranging from 0.9 nM (0.3 ng/mL) against laboratory isolates to less than 100 nM (32.9 ng/mL) for HIV-1 isolates encoding one or more known NNRTI resistance mutations. Dapivirine is equally active against both CCR5 tropic and CXCR4 tropic strains of HIV-1.
- *In vitro* studies in monocyte-derived dendritic cells and autologous CD4+ T-cells, which are important cells in mucosal transmission, indicated that dapivirine is able to prevent viral replication at 10 nM (3.3 ng/mL). Long-term treatment with dapivirine aborted HIV-1 replication in cells infected with cell-free virus at 10 nM (3.3 ng/mL), or those infected by cell-to-cell transmission at 100 nM (32.9 ng/mL).
- In a cervical explant model using tissue from hysterectomised women, dapivirine potently inhibited HIV infection of tissue (IC_{50} = 1.5 nM [0.49 ng/mL]), with > 99% inhibition at 10 nM (3.3 ng/mL). Furthermore, dapivirine inhibited the transfer of HIV from migratory dendritic cells to permissive T-cells with an IC_{50} of 0.1 nM (0.03 ng/mL), and at 100 nM (32.9 ng/mL) transfer was completely inhibited.
- In an *in vivo* hu-SCID mouse model in which mice were treated with dapivirine gel (2.25 to 225 µM [0.7 to 74.1 µg/mL]) intravaginally, and then challenged intravaginally with human peripheral blood lymphocytes infected with either R5 or X4 virus, the gel demonstrated 70-100% protection.

The toxicity of dapivirine has been evaluated in a comprehensive program of preclinical studies. These are described in the IB and included chronic vaginal toxicity studies in rabbits using gel formulations of dapivirine. Studies of up to 26 weeks duration were performed using Dapivirine Gel-001 at concentrations up to 3.3 mg/mL (10 mM; 0.5 mL/day), and studies of up to 39 weeks duration were completed using Dapivirine Gel-002 at concentrations up to 2.0 mg/mL (0.2%; 1.0 mL/day). No local or systemic toxicity was identified in any of these studies. Furthermore, vaginal reproductive toxicity studies in rats and rabbits using

Dapivirine Gel-001 at nominal concentrations up to 3.3 mg/mL (10 mM) or Dapivirine Gel-002 at up to 2.0 mg/mL (0.2%) did not identify any adverse effects on the maternal animals or the developing embryo/foetus.

The no adverse effect level (NOAEL) in rats and dogs following oral administration was 20 mg/kg/day. C_{max} at the NOAEL was 0.39 μg/mL in rats and 1.21 μg/mL in dogs, which more than 300 and 900 times, respectively, the maximum mean plasma concentration (1.212 ng/mL) in women using Ring-003 for 28 days. AUC at the NOAEL was 4.80 μg.h/mL in rats and 12.98 μg.h/mL in dogs, which is over 200 and 500 times, respectively, the mean AUC (22.32 ng.h/mL) in women using Ring-003 for 28 days.

In a safety and pharmacokinetic study in healthy HIV-negative women to assess delivery of dapivirine from both matrix and reservoir intravaginal rings containing 25 mg of dapivirine (IPM 018), the mean maximum dapivirine concentration in cervicovaginal fluids was 2.866 mg/g, and in one subject a concentration of 11 mg/g was detected. These levels were associated with the matrix configuration (much lower levels were observed for the reservoir ring) and occurred at about 24 hours post ring insertion, after which they decreased rapidly. Since the highest gel concentration of dapivirine previously evaluated in intravaginal toxicity studies was approximately 2 mg/mL, an additional 14-day intravaginal study in rabbits was performed at concentrations up to 20 mg/mL. Again, no evidence of local or systemic toxicity was observed.

1.2.3 Clinical Research

To date, 19 phase I and phase I/II clinical studies of dapivirine have been conducted: three studies of dapivirine intravaginal rings in which 49 participants used dapivirine rings, five studies of dapivirine vaginal gel in which 214 participants used dapivirine gel, and 11 studies of oral dapivirine in which 211 participants used oral dapivirine. These clinical studies support the favourable safety profile and tolerability of dapivirine intravaginal ring.

The maximum tolerated dose was established in oral studies as 350 mg for a single dose and 300 mg when administered twice daily for 14 days. The highest daily dose of dapivirine delivered from a vaginal gel to-date (Gel 4750 and Gel 4789, approximately 1250 μg/day for 11 days) is 280 times lower than the MTD for a single dose of oral dapivirine (350 mg) and >600 times lower than the MTD for multiple doses of oral dapivirine (300 mg b.i.d. for 14 days). Similarly, based on the *in vitro* release data, the dose delivered from a dapivirine ring is likely to be far lower than the oral MTD.

No deaths have occurred in any of the studies and no studies were stopped for safety reasons. No related SAEs have been reported to-date.

Adverse events documented in 5 or more participants (>2%) after oral exposure to dapivirine were headache, dizziness, nausea, diarrhoea, fatigue, tremor, somnolence, flatulence, and vomiting. Most of these TEAEs were Grade 1 or Grade 2 and most (≥80%) were considered to be drug-related. Grade 3 TEAEs included headache, dizziness, injury, nausea, tremor, paresthesia, disturbance in attention, abrasion, SGOT increased, SGPT increased, polyuria, fever, diarrhoea, and vomiting. Elevated liver function was documented in laboratory tests. These

increases in SGOT and SGPT were transient and did not result in any liver impairment related to use of the investigational product.

AEs which have been documented in at least 5% of participants in all dapivirine ring and dapivirine gel studies include headache, vaginal haemorrhage, abdominal pain, metrorrhagia, vulvovaginal/genital pruritis, neutropenia, fatigue, abdominal discomfort, vaginal/genital discharge, nasophayngitis, nausea, diarrhoea, urinary incontinence, breast tenderness and blood in urine. Events that were considered related to the dapivirine intravaginal ring include fatigue, vulvovaginal or genital pruritis, abdominal discomfort, abdominal pain, urinary incontinence, nausea, and vaginal or genital discharge.

A complete summary of the safety data from previous preclinical and clinical studies of dapivirine via the oral and vaginal routes and the different dosage forms are contained in the Dapivirine Vaginal Ring IB (7).

1.2.4 Formulation of silicone elastomer vaginal matrix ring containing 25mg of dapivirine

The dapivirine intravaginal matrix ring (Ring-004) is an off-white flexible ring containing 25 mg of drug substance dispersed in a platinum-cured silicone matrix. The dimensions of the ring are 56 mm and 7.6 mm, outer diameter and cross-sectional diameter, respectively. The dapivirine ring is designed to provide sustained release over a 28-day period.

1.3 Rationale for Protocol IPM 015

The mission of IPM is to accelerate the development of and access to safe and effective topical microbicides for the prevention of HIV infection among women in developing countries in accordance with high standards of quality. Based on *in vitro*, *in vivo*, and *ex vivo* studies described in the Dapivirine intravaginal Ring IB, dapivirine shows great promise as a topical microbicide to prevent HIV-1 infection.

Multiple safety and efficacy trials with various microbicides have been completed or are currently underway, most of which evaluate microbicides in gel formulation delivered via a single-use vaginal applicator used prior to coitus. In order for a microbicide to be most effective, it is essential that it is used correctly. Therefore, it is important that a microbicide is acceptable to users, and it is likely that products that can be applied less frequently will be more acceptable and will achieve better user-compliance. Intravaginal rings that need only be replaced every 28 days may therefore have benefits over dosage forms that need to be used more frequently.

Intravaginal rings have already been developed and approved as delivery methods for medications. Femring® (estradiol acetate vaginal ring), a hormone replacement product approved in June 2003 by the United States (U.S.) Food and Drug Administration (FDA) treats menopause-induced vasomotor symptoms (e.g., hot flashes) and symptoms of vulvar and vaginal atrophy (e.g., dryness) (8). *Femring* is a silicone elastomer ring which contains estradiol acetate and is approved for 3 continuous months of use. Although these rings are not exactly the same as the IPM ring, the safety of the silicone elastomer ring has been established through extensive clinical studies and post-marketing experience. Moreover, a recent acceptability study of the silicone elastomer ring used in *Femring* (but containing

no drug) among postmenopausal women in the U.S. demonstrated very high acceptability and ease of use (9).

IPM 015 has been developed to assess the safety of administration of a silicone elastomer intravaginal matrix ring containing 25mg of dapivirine inserted once every 28 days over a 12-week period among healthy, sexually active, HIV-negative women as compared with a placebo intravaginal ring.

2.0 STUDY OBJECTIVES

The primary objective is:

- To assess and compare the safety of a silicone elastomer intravaginal matrix ring containing 25 mg of dapivirine and a placebo intravaginal ring inserted once every 28 days over a 12 week period among healthy, sexually active, HIV-negative women.

The secondary objectives are:

- To assess the acceptability of an intravaginal ring inserted once every 28 days over a 12-week period among healthy, sexually active, HIV-negative women.
- To assess women's adherence to the use of an intravaginal ring inserted once every 28 days over a 12-week period.

The exploratory objectives are:

- To assess sexual behaviours and possible factors influencing adherence to the use of an intravaginal ring inserted once every 28 days over a 12-week treatment period.
- To explore potential methods that may be used for adherence determination in Phase III studies.

3.0 OVERALL STUDY DESIGN

3.1 Study Design

IPM 015 is a double-blind, randomized (1:1 allocation), placebo-controlled study conducted in Kenya, Malawi, Rwanda, South Africa, Tanzania and Zambia among approximately 280 healthy, sexually active, HIV-negative women to assess and compare the safety of 28 day use of a silicone elastomer intravaginal matrix ring containing dapivirine and a placebo intravaginal ring containing no dapivirine.

Not including screening, all enrolled participants will have 6 scheduled clinic visits as follows:

- Visit 1: Week 0 - **ENROLMENT VISIT,** first ring inserted
- Visit 2: 2 weeks post enrolment
- Visit 3: 4 weeks post enrolment - first ring removed, second ring inserted
- Visit 4: 8 weeks post enrolment – second ring removed, third ring inserted
- Visit 5: 12 weeks post enrolment – third ring removed
- Visit 6: 16 weeks post enrolment - **LAST STUDY VISIT**

All visits except for the enrolment visit have a study window of ±4 days. For details regarding specific study visit procedures, refer to Section 4.0 Study Visits and Procedures.

Safety will be assessed through self-reported adverse events, findings at pelvic/speculum examinations (with and without colposcopy), and laboratory test determinations (including STI investigations). All adverse events will be monitored until resolution and/or the cause is identified or until the investigator does not expect any improvement or worsening of condition/symptoms. If an adverse event remains unresolved at the participant's last study visit, a clinical assessment will be made by the investigator and discussed with the IPM Clinical Safety Physician or designee to determine whether continued follow-up of the adverse event is warranted. The incidence of STIs will be determined at screening and 28 day thereafter. HIV and pregnancy testing will be performed at screening and each study visit. Vaginal flora and vaginal pH will be assessed before initial ring insertion and at Visits 3, 4 and 5 at the time of ring removal.

During the course of the study, participants will receive HIV pre- and post-test counselling, HIV/STI risk reduction counselling (and provision of condoms), contraceptive counselling, and ring adherence counselling.

Behavioural assessments will be conducted at every scheduled visit, including sexual behaviour, intravaginal practices and condom use at weeks 0, 4, 8, 12, and 16 (visits 1, 3, 4, 5 & 6). Plasma dapivirine levels will be obtained at weeks 0, 4 and 12 (visits 1, 3 & 5). Residual dapivirine levels will be measured in the rings returned used or unused at weeks 4, 8 &12 (visits 3, 4 & 5). Acceptability assessments will be administered at weeks 0, 4 and 12 (visits 1, 3 & 5) and Adherence assessments will be administered at weeks 2, 4, 8 and 12 (visits 2, 3, 4 & 5). To assess the effect of the intravaginal ring on vaginal flora and vaginal pH, specimens will be taken at enrolment prior to initial ring insertion, and at weeks 4, 8 and 12 (visits 3, 4 & 5) after ring removal. Adverse events will be assessed at all visits post randomization.
This study will be conducted in accordance with the ethical principles of the World Medical Association Declaration of Helsinki, the International Conference on Harmonisation (ICH) Good Clinical Practice (GCP) guidelines and any applicable local regulatory requirements (12-13) e.g., the *Guidelines for Good Practice in the Conduct of Clinical Trials in Human Participants in South Africa* and *Ethics in Health Research*.

3.2 Study Duration

The maximum allowable time between screening and enrolment per participant is 28 days. Following enrolment into the study, each participant will be followed on-study for a total of 16 weeks (12 weeks ring use and a follow-up visit 4 weeks post ring removal). It is anticipated that full enrolment will be completed in 24 weeks per research centre for a total of up to 40 weeks study duration. If research centres are unable to complete enrolment within 7 months of their Site Initiation Visit, further enrolment at the research centre may be terminated.

3.3 Study Population

This study will enrol HIV-negative, sexually active women 18-40 years of age, who in the opinion of the Investigator understand the study and can provide informed

consent. Approximately 280 (16 – 40 per research centre) women will be enrolled in total.

3.3.1 Inclusion Criteria

Women must meet all of the following criteria to be eligible for study enrolment:

1. Women ≥18 and ≤40 years of age who can provide informed consent;

2. Available for all visits and consent to follow all procedures scheduled for the study;

3. Healthy and self-reported sexually active (defined as an average of one penetrative penile vaginal coital act per month for the last 3 months prior to enrolment);

4. HIV-negative as determined by an HIV rapid test at screening and enrolment;

5. On a stable form of contraception defined as:
   - Oral contraceptive regimen for at least 3 months prior to enrolment, OR,
   - Transdermal contraceptive patch for at least 3 months prior to enrolment, OR,
   - Long acting progestins for at least 6 months prior to enrolment, OR,
   - An IUD inserted (with no vaginal or gynaecological complaints associated with its use) at least 3 months prior to enrolment, AND

   Willing to continue on this stable method of contraception, OR,
   - Have undergone surgical sterilisation at least 3 months prior to enrolment;

6. In the absence of the use of exogenous hormone(s), have a self-reported regular menstrual cycle defined as having a minimum of 21 days and a maximum of 35 days between menses;

7. Upon pelvic/speculum examination and colposcopy at the time of enrolment, the cervix and vagina appear normal as determined by the investigator;

8. Asymptomatic for genital infections at the time of enrolment (if a woman is diagnosed with any treatable STI either syndromically or by laboratory test during the screening period, she must have received treatment at least 2 weeks prior to enrolment);

9. Willing to refrain from use of vaginal products or objects including tampons, female condoms, cotton wool, rags, diaphragms, cervical caps (or any other vaginal barrier method), douches and drying agents within 14 days from enrolment and for the duration of the study;

10. Willing to answer to acceptability, adherence and behavioural assessments throughout the study.

11. Willing to refrain from participation in any other research study for the duration of their participation in IPM015;

12. Willing to provide adequate locator information for study retention purposes and be reachable per local standard procedures (e.g., by home visit or telephone; or via family or close neighbour contacts [confidentiality to be maintained]).

3.3.2 Exclusion Criteria

Women who have any of the exclusion criteria below are not eligible:

1. Currently pregnant or last pregnancy within 3 months prior to enrolment;
2. Currently breast-feeding;
3. Participated in any other research study within 60 days prior to screening;
4. Previously participated in any HIV vaccine study.
5. Untreated urogenital infections (either symptomatic or asymptomatic), e.g., urinary tract or other sexually transmitted infections, or other gynaecological symptoms, e.g., vaginal itching, pain, or discharge, within 2 weeks prior to enrolment;
6. Presence of any abnormal physical finding on the vulva, vaginal walls or cervix during pelvic/speculum examination and/or colposcopy;
7. History of significant urogenital or uterine prolapse, undiagnosed vaginal bleeding, or urethral obstruction, incontinence or urge incontinence;
8. Pap smear result at screening that requires cryotherapy, biopsy, treatment (other than for infection), or further evaluation [this includes any findings of atypical squamous cells of undetermined significance (ASCUS)];
9. Any Grade 2, 3 or 4 baseline (screening) haematology, chemistry or urinalysis laboratory abnormality according to the DAIDS Table for Grading Adverse Events (**NOTE:** *This table can be found at: http://rcc.tech-res.com/tox_tables.htm and a standardized version will be provided to research centres in the Study Operations Manual*);
10. Any abnormal bleeding per vagina, bleeding per vagina during or following vaginal intercourse, or gynaecologic surgery within 90 days prior to enrolment;
11. Any history of anaphylaxis or severe allergy resulting in angioedema; or a history of sensitivity/allergy to latex or silicone elastomer;
12. Any serious acute, chronic or progressive disease (e.g. any known history of neoplasm, cancer, insulin-dependent diabetes, cardiac disease, autoimmune disease or blood dyscrasias), or with signs of cardiac disease, renal failure, or severe malnutrition;
13. Any condition(s) that, in the opinion of the investigator, might interfere with adherence to study requirements or evaluation of the study objectives.

NOTE: *Women who fail screening may be re-screened a maximum of once and may be enrolled if they are found to meet ALL inclusion and NO exclusion criteria at the second screening visit.*

4.0 STUDY VISITS AND PROCEDURES

4.1 Screening Visit

a. Explain screening and study procedures to potential participant.

b. If potential participant agrees to participate, obtain written informed consent (illiterate participants may provide a thumbprint or mark witnessed and signed by a person independent from study staff).

c. Assign unique Screening Identification number (SCR-ID) to potential participant in sequential order. SCR-ID numbers should never be reassigned. A master log of screening participants with SCR-ID, demographic and locator information must be maintained and kept in a locked/secure location to track potential participants who have been screened for the study.

d. Obtain and record demographic information, relevant medical history, concomitant medication (taken within the last 30 days), and locator and menses information. (Refer to Section 5.6 for more details about relevant medical history).

   NOTE: *Because pelvic exam & colposcopy should not be done during or within 2 days of completing menstruation, carefully plan the date of randomization so that menses does not occur on the visits when colposcopy evaluations are scheduled at weeks 0, 4, 8 and 12 (visits 1, 3, 4 & 5).*

e. Conduct preliminary review of inclusion/exclusion criteria with potential participant, e.g., whether sexually active, etc.

f. Provide HIV/STI risk-reduction counselling (including dispensing of condoms) and contraceptive counselling. (Refer to Sections 5.2.2 and 5.4, respectively for further details).

g. Provide HIV pre- and post-test counselling. (Refer to Section 5.2.1 for further details).

h. Perform HIV testing as detailed in section 5.3.

   NOTE: *If a woman is confirmed HIV positive, she is not eligible to enrol in the trial and no further screening procedures will be performed.*

i. Obtain urine sample for pregnancy and urinalysis (dipstick and microscopy). Refer pregnant women to local prenatal clinic for support services. (Refer to Sections 5.5 and 5.10 for further details.) **NOTE:** *Participant is excluded from study participation if she is pregnant or has any Grade 2, 3 or 4 laboratory abnormality according to the DAIDS Table for Grading Adverse Events*.

j. Perform general physical examination. Refer to Section 5.6 for a description of the elements required in the general physical examination.

k. Perform pelvic/speculum examination (Refer to Section 5.7). If the pelvic/speculum examination indicates abnormal findings, e.g., any symptoms or evidence of genital infections, abrasions, ulcerations, etc., provide or refer for appropriate treatment. **NOTE:** *If the potential participant is menstruating at the time of screening, the pelvic/speculum examination with sample collection should be rescheduled to two days after completion of menses*.

l. Collect cervico-vaginal swabs for STI testing, including BV (Refer to Section 5.11) and a Pap smear (Refer to Section 5.13). **NOTE:** *If participant tests positive for any STI, provide or refer her for appropriate treatment. If Pap smear results require cryotherapy, biopsy, treatment (other than for infection) or further evaluation including any findings of atypical squamous cells of undetermined significance (ASCUS), the participant is ineligible for the study but should be referred for appropriate treatment.*

m. Collect blood samples (approximately 10-15 ml) by venipuncture for syphilis testing (RPR) and the following safety laboratory tests:

    - haematology (FBC with differential and platelet count),
    - chemistry (sodium, potassium, phosphate, chloride, and calcium, renal function (urea, creatinine), liver function (total bilirubin, ALT (SGPT), AST (SGOT), ALP, and GGT).

   (Refer to Sections 5.10 for further details.)

   NOTE: *Participant is not eligible to enrol if she has any Grade 2, 3 or 4 laboratory abnormality according to the DAIDS Table for Grading Adverse Events (available at: http://rcc.tech-res.com/tox_tables.htm and a standardized version will be provided to research centres in the Study Operations Manual)*

n. Provide and explain diary card to potential participants.

o. Based on continued study eligibility, invite potential participant to return to clinic within 28 days for the enrolment visit.

4.2 Visit 1 = ENROLMENT VISIT

(Within 28 days of Screening)

NOTE: *If potential participant is menstruating at this visit, the entire visit should be rescheduled for two days after completion of menses but must be completed within 28 days of the screening visit.*

4.2.1 Pre-Enrolment Procedures

a. Obtain and record any medical problems and concomitant medication since the last visit. ***NOTE:*** *Record any conditions as part of the Relevant Medical History. See Section 5.6.*

b. Obtain locator and menses information.

c. Provide HIV/STI risk-reduction counselling (including dispensing of condoms) and contraceptive counselling. (Refer to Sections 5.2.2 and 5.4, respectively for further details).

d. Provide HIV pre- and post-test counselling. (Refer to Section 5.2.1 for further details).

e. Perform HIV testing as detailed in section 5.3.

   NOTE: *If a woman is confirmed HIV positive, she is not eligible to enrol in the study and no further screening procedures will be performed.*

f. Collect a urine sample for pregnancy testing. If a woman is pregnant, she is not eligible for the study. Refer pregnant women to the local prenatal clinic for support services (see Section 5.5).

g. If the HIV and urine pregnancy tests are negative, perform pelvic/speculum examination with colposcopy (Refer to Sections 5.7 and 5.8 for further details). ***NOTE:*** *The potential participant must be asymptomatic for genital infections (and have received treatment at least 2 weeks prior to enrolment for any STI diagnosed clinically or by laboratory test) and have a normal pelvic/speculum examination with colposcopy at the time of enrolment.*

h. Collect blood specimen (approximately 5ml) by venipuncture for HIV RNA PCR testing (to be tested only if participant is enrolled).

i. Collect blood specimen (approximately 10ml) by venipuncture for measurement of plasma dapivirine levels. (Refer to Section 5.17).

j. At time of pelvic/speculum exam (Refer to Section 5.7), collect specimens for determination of vaginal flora (to be tested only if participant is enrolled), and vaginal pH (Refer to Section 5.12).

k. If all inclusion criteria and none of the exclusion criteria continue to be met, invite woman to enrol immediately.

4.2.2 Enrolment Procedures

a. If potential participant agrees to enrol in the study, obtain written informed consent again (illiterate participants may provide a thumbprint or mark witnessed and signed by a person independent from study staff).

b. Assign unique Participant Identification Number (PID) to participant in sequential order.

c. Administer a baseline behavioural assessment, including sexual behaviour, vaginal practice condom use and product acceptability.

d. Administer the baseline acceptability assessment.

e. Dispense one intravaginal ring to the participant.

f. Instruct the participant to insert the intravaginal ring (Refer to section 5.14 for further details). Perform brief digital examination to verify the intravaginal ring has been properly placed (Refer to Section 5.8 for further details).

g. After ring insertion, participant will remain in the clinic under observation for 30 minutes to observe for immediate reactions.

h. Provide intravaginal ring adherence counselling. (Refer to Section 5.15 for further details). Participants will be instructed to keep the ring inserted for a period of 28 consecutive days (4 weeks). If the ring falls out during this period, the participant should wash her hands, rinse ring thoroughly in lukewarm water and re-insert it. Participant to make a note on the diary card. (Refer to Section 5.16 for further details.)

i. Review diary card and if the participant is a screening failure, dairy card will be collected.

j. Schedule next visit.

4.3 Visit 2 = Week 2 Post Enrolment

(Window ±4 days)

NOTE: *If participant is menstruating or anticipates menstruation at a visit, the entire visit should be rescheduled for two days after completion of menses. If participant reports unexpected bleeding at any visit, a pelvic examination, with colposcopy as indicated, should be performed to assess the source of bleeding and a follow up examination should be scheduled upon resolution of bleeding.*

a. Obtain and record any adverse events and concomitant medications since the last visit.

b. Update locator and menses information as necessary.

c. Provide HIV/STI risk reduction counselling (including dispensing of condoms) and contraceptive counselling. (Refer to Sections 5.2.2 and 5.4 respectively for further details).

d. Provide pre- and post-test HIV counselling. (Refer to Section 5.2.1 for further details).

e. Perform HIV testing as detailed in section 5.3.

   NOTE: *If a participant is confirmed HIV positive, she will immediately discontinue use of the investigational product. She will be counselled and referred for social services and other clinically indicated medical services. She may continue to attend regular scheduled visits and will undergo procedures as appropriate for safety for the remainder of the study.*

f. Obtain urine sample for pregnancy testing. If the urine pregnancy test is positive, the ring will be removed, and the participant will be referred to the local prenatal clinic for support services (Refer to Section 5.5 for further

details). Study procedures will be performed as appropriate for safety for the remainder of the 16 weeks.

g. Administer adherence assessment.

h. Instruct participant to remove the intravaginal ring (Refer to section 5.14 for further details) and perform pelvic/speculum examination (Refer to Section 5.7 for further details). Following the examination, instruct participant to rinse ring thoroughly in lukewarm water, if desired, and to reinsert the ring.

i. Perform brief digital exam to ensure ring is properly placed (Refer to section 5.8 for further details). Clinician to document presence of the ring in situ and correct removal and reinsertion by participant.

j. Administer intravaginal ring adherence counselling (Refer to Section 5.15 for further details). Participants will be instructed to keep the ring inserted for a period of 28 consecutive days (4 weeks). If the ring falls out during this period, the participant should wash her hands, rinse ring thoroughly in lukewarm water and re-insert it. Participant to make a note on the diary card. (Refer to Section 5.16 for further details.)

k. Review diary card.

l. Schedule next visit.

4.4 Visit 3 = Week 4 Post Enrolment

(Window ±4 days)

NOTE: *If participant is menstruating or anticipates menstruation at a visit, the entire visit should be rescheduled for two days after completion of menses. If participant reports unexpected bleeding at any visit, a pelvic examination, with colposcopy as indicated, should be performed to assess the source of bleeding and a follow up examination should be scheduled upon resolution of bleeding.*

a. Obtain and record any adverse events and concomitant medications since the last visit.

b. Update locator and menses information as necessary.

c. Administer follow-up behavioural assessment, including sexual behaviour, condom use and vaginal practice

d. Administer product adherence assessment

e. Administer product acceptability assessment.

f. Provide HIV/STI risk-reduction counselling (including dispensing of condoms) and contraceptive counselling. (Refer to Sections 5.2.2 and 5.4, respectively for further details).

g. Provide HIV pre- and post-test HIV counselling. (Refer to Section 5.2.1 for further details).

h. Perform HIV testing as detailed in section 5.3.

   NOTE: *If a participant is confirmed HIV positive, she will immediately discontinue use of the investigational product. She will be counselled and referred for social services and other clinically indicated medical services. She may continue to attend regular scheduled visits and will undergo procedures as appropriate for safety for the remainder of the study.*

i. Obtain urine specimen for pregnancy testing and urinalysis (dipstick and microscopy). If the pregnancy test is positive, remove the ring and refer the participant to the local prenatal clinic for support services. (Refer to Section 5.5 for further details). Study procedures will be performed as appropriate for safety for the remainder of the 16 weeks.

j. Collect blood specimen (approximately 10ml) by venipuncture for measurement of plasma dapivirine levels (Refer to Section 5.17).

k. Collect blood specimen (approximately 10ml) by venipuncture for safety laboratory tests (haematology and chemistry).

l. Instruct participant to remove the intravaginal ring. The returned used or unused ring will be shipped at IPM's request to an analytical laboratory for testing of residual dapivirine levels.

m. Perform pelvic/speculum examination (Refer to section 5.7 for further details) *with colposcopy* (Refer to section 5.9 for further details) and collect cervico-vaginal swabs for STI testing. (Refer to section 5.11 for further details). Collect vaginal specimens for determination of vaginal flora and vaginal pH.

n. Dispense new intravaginal ring and instruct participant to self-insert ring (Refer to section 5.14 for further details). Perform brief digital exam to ensure that ring is properly in place. Clinician documents presence of ring in situ and correct removal and reinsertion of the new ring by participant.

o. Administer intravaginal ring adherence counselling (Refer to Section 5.15 for further details). Participants will be instructed to keep the ring inserted for a period of 28 consecutive days (4 weeks). If the ring falls out during this period, the participant should wash her hands, rinse ring thoroughly in lukewarm water and re-insert it. Participant to make a note on the diary card. (Refer to Section 5.16 for further details.)

p. Review diary card.

q. Schedule next visit.

4.5 Visit 4 = Week 8 Post Enrolment

(Window ±4 days)

NOTE: *If participant is menstruating or anticipates menstruation at a visit, the entire visit should be rescheduled for two days after completion of menses. If*

participant reports unexpected bleeding at any visit, a pelvic examination, with colposcopy as indicated, should be performed to assess the source of bleeding and a follow up examination should be scheduled upon resolution of bleeding

a. Obtain and record any adverse events and concomitant medications since the last visit.

b. Update locator and menses information as necessary.

c. Administer follow up behavioural assessments: including sexual behaviour, condom use and vaginal practice

d. Administer adherence assessment

e. Provide HIV/STI risk-reduction counselling (including dispensing of condoms) and contraceptive counselling. (Refer to Sections 5.2.2 and 5.4, respectively for further details).

f. Provide HIV pre- and post-test HIV counselling. (Refer to Section 5.2.1 for further details).

g. Perform HIV testing as detailed in section 5.3.

   NOTE: *If a participant is confirmed HIV positive, she will immediately discontinue use of the investigational product. She will be counselled and referred for social services and other clinically indicated medical services. She may continue to attend regular scheduled visits and will undergo procedures as appropriate for safety for the remainder of the study.*

h. Obtain urine sample for pregnancy testing and urinalysis (dipstick and microscopy). If the pregnancy test is positive, remove the ring and refer the participant to the local prenatal clinic for support services. (Refer to Section 5.5 for further details). Study procedures will be performed as appropriate for safety for the remainder of the 12 weeks.

i. Collect blood specimen (approximately 10ml) by venipuncture for safety laboratory tests (haematology and chemistry).

j. Instruct participant to remove the intravaginal ring. Clinician to document presence of the old ring in situ, correct removal and reinsertion of the new ring by participant. The returned used or unused ring will be shipped at IPM's request to an analytical laboratory for testing of residual dapivirine levels..

k. Perform pelvic/speculum examination (Refer to section 5.7 for further details) *with colposcopy* (Refer to section 5.9 for further details) and collect cervico-vaginal swabs for STI testing (Refer to section 5.11 for further details). Collect vaginal specimens for determination of vaginal flora and vaginal pH.

l. Dispense new intravaginal ring and instruct participant to self-insert ring. Perform brief digital exam to ensure that ring is properly in place.

m. Administer intravaginal ring adherence counselling (Refer to Section 5.15 for further details). Participants will be instructed to keep the ring inserted for a

period of 28 consecutive days (4 weeks). If the ring falls out during this period, the participant should wash her hands, rinse ring thoroughly in lukewarm water and re-insert it. Participant to make a note on the diary card. (Refer to Section 5.16 for further details.)

n. Review diary card.

o. Schedule next visit.

4.6 Visit 5 = Week 12 Post Enrolment

(Window ±4 days)

NOTE: *If participant is menstruating or anticipates menstruation at a visit, the entire visit should be rescheduled for two days after completion of menses. If participant reports unexpected bleeding at any visit, a pelvic examination, with colposcopy as indicated, should be performed to assess the source of bleeding and a follow up examination should be scheduled upon resolution of bleeding*

a. Obtain and record any adverse events and concomitant medications since the last visit.

b. Update locator and menses information as necessary.

c. Administer follow-up behavioural assessment, including sexual behaviour, condom use and vaginal practice.

d. Administer product adherence assessment.

e. Administer product acceptability assessment.

f. Collect blood specimen (approximately 10ml) by venipuncture for plasma dapivirine levels (Refer to section 5.17).

g. Provide HIV/STI risk-reduction counselling (including dispensing of condoms) and contraceptive counselling. (Refer to Sections 5.2.2 and 5.4, respectively for further details).

h. Provide HIV pre- and post-test HIV counselling. (Refer to Section 5.2.1 for further details).

i. Perform HIV testing as detailed in section 5.3.

   NOTE: *If a participant is confirmed HIV positive, she will immediately discontinue use of the investigational product. She will be counselled and referred for social services and other clinically indicated medical services. She may continue to attend regular scheduled visits and will undergo procedures as appropriate for safety for the remainder of the study.*

j. Obtain urine sample for urinalysis (dipstick and microscopy).

k. Collect blood specimen (approximately 10ml) by venipuncture for serum pregnancy test and safety laboratory tests (haematology and chemistry). If the pregnancy test is positive, refer participant to the local prenatal clinic for support services. (Refer to Section 5.5 for further details).

l. Instruct participant to remove the intravaginal ring. Clinician to document presence of the ring in situ and correct removal by participant.

m. The returned used or unused ring will be shipped at IPM's request to an analytical laboratory for testing of residual dapivirine levels. ***NOTE****: This concludes the use of the intravaginal ring*. *DO NOT dispense a new intravaginal ring*.

n. Perform general physical exam.

o. Perform pelvic/speculum examination (Refer to section 5.7 for further details) *with colposcopy* (Refer to section 5.9 for further details) and collect cervico-vaginal swabs for STI testing (Refer to section 5.11 for further details.) Collect vaginal specimens for determination of vaginal flora and vaginal pH.

p. Review and collect diary card.

q. Schedule next visit.

4.7 Visit 6 = Week 16 Post Enrolment and LAST STUDY VISIT

(Window ±4 days)

NOTE: *If participant is menstruating or anticipates menstruation at a visit, the entire visit should be rescheduled for two days after completion of menses. If participant reports unexpected bleeding at any visit, a pelvic examination, with colposcopy as indicated, should be performed to assess the source of bleeding and a follow up examination should be scheduled upon resolution of bleeding*

a. Obtain and record any adverse events and concomitant medications since the last visit.

b. Update locator and menses information if necessary.

c. Provide HIV/STI risk reduction counselling (including dispensing of condoms). (Refer to Sections 5.2.2 for further details).

d. Obtain urine sample for pregnancy testing. If the pregnancy test is positive, refer participant to the local prenatal clinic for support services. (Refer to Section 5.5 for further details).

e. Administer follow -up behavioural assessment, including sexual behaviour, condom use and vaginal practice

f. Perform pelvic/speculum examination as indicated.

g. Provide HIV pre- and post-test HIV counselling. (Refer to Section 5.2.1 for further details.)

h. Collect blood specimen (approximately 5ml) for HIV RNA PCR testing. If the HIV RNA PCR test is reactive, test blood collected at Visit 5 and refer participant to local centre for HIV treatment and support services.

i. Study participation is concluded.

4.8 Unscheduled Visits

Unscheduled visits may be performed at any time during the study for HIV or pregnancy testing, or if the participant is experiencing any problems, e.g., vaginal complaints, difficulties with re-inserting the ring in cases of accidental expulsion, or accidental loss of the ring. Participants will be evaluated and treated appropriately. Referral for treatment at an outside medical facility will be made if necessary.

All unscheduled visits will be documented in the source documents and applicable CRFs.

4.9 Missed & Late Visits

Study staff will make every effort to contact participants to return to clinic for scheduled visits. If a participant does not return to clinic for a scheduled visit during the study window, e.g., within ±4 days of a scheduled visit, continued attempts to contact the participant should be made and documented in the source documents and applicable CRFs.

If the participant does not return to clinic for a scheduled visit prior to the start of the study window of the next visit, the visit will be considered missed. For example, if a participant does not return for Visit 3 by the time the study window has begun for Visit 4, i.e., within 4 days from Visit 4, Visit 3 will be considered missed. Study procedures will be performed as scheduled for the current visit (e.g. Visit 4 in this instance) or as deemed necessary by the Investigator or IPM Clinical Safety Physician. Missed visits will be documented as protocol deviations.

If a participant comes in for a visit outside the window for a scheduled trial visit but prior to the start of the window for the next visit, it should be treated as a late visit and documented as a protocol deviation. All study procedures scheduled for the last scheduled visit should still be performed. The participant should then be put back on her original visit schedule.

4.10 Early Discontinuation Visit

Participants may be discontinued early from the study prior to completion of the last study visit (Visit 6) for any of the following reasons:

- Participant withdraws her consent
- Participant fails to follow protocol requirements which are deemed to be serious enough by the investigator to warrant a discontinuation, e.g., in the absence of an adverse event or discomfort, participant refuses to keep intravaginal ring inserted for duration of the study
- Participant is lost to follow-up, i.e., research centre is unsuccessful in contacting participant or bringing participant back to clinic and participant misses 3 consecutive visits

- At the discretion of the Investigator, Sponsor, IRB/IEC or the government health agency

NOTE: The Safety Evaluation Committee may provide recommendations to the Sponsor or to the investigators regarding participants who should be discontinued, or allowed to continue in the study.

The date, time, and reason for permanent study discontinuation are to be noted in the source documents and applicable CRFs. All participants who prematurely discontinue from the study should be encouraged to return to clinic for a final evaluation, at which time all study procedures scheduled for Visit 5 (Week 12) should be performed.

Participants who miss three (3) consecutive study visits will be considered lost to follow-up and will be permanently discontinued from the study. Contact attempts and final early study termination will be documented in the source documents and applicable CRFs. If a participant already considered lost to follow-up returns to clinic prior to research centre study completion, the clinic chart (including CRFs) may be re-opened to perform study discontinuation procedures.

Participants who discontinue early from the study will not be replaced.

4.11 Premature Discontinuation of the Study

The Sponsor has the right to discontinue this study at any time for any reason. If the clinical study is prematurely discontinued, the Investigator must promptly inform the participants and IRB/IECs, and ensure medical follow-up of participants in consultation with the Sponsor. If the study is prematurely discontinued, all procedures and requirements pertaining to the archiving of documents will be observed. The Sponsor will provide the research centres with instructions on the proper disposition of any clinical supplies remaining at the research centre.

5.0 STUDY PROCEDURE DETAILS

5.1 INFORMED CONSENT

The informed consent form will describe the purpose of the study, the procedures to be followed, and the risks and benefits of participation. Two consent forms will be administered: a screening consent form and a study consent form.

The informed consent process should include adequate time for each potential participant to have any study questions answered by the study staff including a qualified physician who is an IPM 015 investigator, and the entire process will be documented in the source documents.

At screening, potential participants who agree to participate in the study will sign and date the screening consent form which describes the screening procedures. The form will be signed and dated by the person administering the consent form and must also be countersigned and dated by a qualified physician who is an investigator in the IPM 015 study to document his/her involvement in the informed consent process. If a potential participant is functionally illiterate, the consent document(s) and any written study-related materials must be read to her in the language best understood by the potential participant in the presence of an impartial literate observer not affiliated with the study. After the potential participant

has orally consented and provided a thumbprint or mark which is witnessed by the impartial observer, this independent observer will sign and date the consent form as a witness.

During the enrolment visit, eligible participants who agree to participate in the study will sign and date the study consent form which describes all the visits and procedures to be followed on the study. It will be signed and countersigned in the same manner as the screening consent form.

The signed and dated consent forms must be retained at the study research centre. A copy of the signed and dated consent forms will be offered to the participant. If the participant is not willing to receive the forms, the second copy will be retained at the research centre. Likewise during the study, signed and dated consent form updates and any amendments to written study-related materials to be given to participants will be offered to the participant but retained at the research centre if the participant is unwilling to receive the forms.

Documentation of the participant's refusal to accept a copy of the informed consent or other study-related materials should be noted in the source documents.

The consent forms and any study-related materials given to the participant will be translated and back-translated in the local languages by a certified translator, if available in country, or according to local Ethics Committee requirements and regulatory authority guidelines. The information will be verbally communicated in the local language by the study staff. Documentation will be required to verify who performed translation/back-translation of the materials as well as a written statement by the translator indicating that the consent form(s) is an accurate translation of the accompanying English version. This is the Principal Investigator's responsibility.

All research centre specific consent forms must first be reviewed and approved by IPM and then approved by the responsible Institutional Review Board (IRB) and/or local Institutional Ethics Committee (IEC) prior to administration to the participants.

If new information becomes available which may be relevant to the participant's willingness to continue study participation, the information will be provided via an IRB and/or IEC-approved revised consent form or addendum to the original consent form in a timely manner and must be signed and dated by the participant in the same manner described above.

5.2 HIV Counselling

5.2.1 HIV Pre- and Post-Test Counselling

At screening and all study visits where HIV testing is performed, pre- and post-test counselling will be provided according to the CDC Revised Guidelines for HIV Counselling and Testing (12). Adaptations of these guidelines in accordance with locally accepted standards of practice are allowed. Each study research centre will document the counselling policies and procedures prior to study implementation for purposes of staff training, quality assurance, and study monitoring.

A comprehensive package of post-test counselling and psychosocial support will be provided to women who test HIV reactive at any point during study participation. Initial counselling services will be provided at the research centre and

women will be referred for additional counselling, support services and, where available, treatment. These services will be identified by IPM prior to study initiation and referral procedures will be documented in writing by the research centre.

5.2.2 HIV/STI Risk Reduction Counselling

HIV/STI risk reduction guidelines will be developed in conjunction with local voluntary counselling and testing (VCT) guidelines. Counselling will be provided at all study visits including screening. Efforts will be made to ensure the highest quality of risk reduction counselling at the study clinics.

Risk reduction counselling will include recommendation of condom use. Participants will be provided with a regular supply of male non-spermicidal condoms during each study visit.

5.3 HIV Testing & Management

SCREENING
At screening, potential participants will be tested using a highly sensitive HIV Rapid test (Test 1). If the test result is non-reactive, the participant could potentially be enrolled in the study. If Test 1 is reactive, the potential participant will be retested using a highly specific HIV Rapid test (Test 2). If Test 2 is reactive, the woman is considered to be HIV infected and not eligible for enrolment. She will becounselled and referred to local health facilities for social support or other clinically indicated medical services
If Test 2 is non-reactive, (yielding discordant results from Test 1), the result will be considered indeterminate and the potential participant will be counselled and asked to return for repeat testing for the study after two weeks.

If Test 1 is non-reactive at this repeat testing visit, and after HIV counselling ruling out recent exposure that could be masked during the window period, the potential participant is considered to be HIV negative and is eligible for enrolment if all other inclusion/exclusion criteria are met. If Test 1 is reactive at this repeat testing visit, Test 2 will be performed, however, regardless of the outcome, she will be considered ineligible for the study. If Test 2 is reactive, she will be considered HIV infected and will be counselled and referred to local health facilities for social support or other clinically indicated medical services. If the Test 2 is non-reactive the HIV status will be considered indeterminate, she will receive confirmatory testing and be referred to local health facilities for follow up testing or other clinically indicated medical services as per local guideline.

NOTE: If the national regulatory authority of the country in which the research centre is situated requires that a national testing algorithm be used during the screening process, this will be performed as part of the screening process.

The following table describes the possible HIV testing results and outcomes:

HIV Testing during Screening:

Test 1	Test 2	Final Outcome	
Non-reactive	N/A	**Not HIV Infected**	**Eligible to enrol**
Reactive	Reactive	**HIV Infected**–Refer for counselling and confirmatory testing as per local standard of care and research centre specific SOPs	**Do NOT enrol**
Reactive	Non-reactive	**Indeterminate**	**Perform HIV ELISA test if applicable or consider repeat testing in 2 weeks**

ENROLMENT

During pre-enrolment, the participant will be tested for HIV and only enrolled if the result is considered to be HIV negative and she is otherwise eligible.
Test 1 will be performed. If the test is non-reactive, and after HIV counselling ruling out recent exposure that could be masked during the window period, the potential participant will be considered HIV negative and is eligible for enrolment. At this stage, 5ml of blood will be obtained by venipuncture to be sent for quantitative HIV RNA PCR testing to confirm the HIV-negative status of all enrolled participants. Any participant who has a positive enrolment HIV RNA PCR will be considered to have been HIV infected (but HIV seronegative) at enrolment and will be discontinued from intravaginal ring use. She may continue to attend scheduled clinic visits and study procedures will be performed as appropriate for safety for the remainder of the study period.

If HIV Test 1 is reactive, the potential participant will be retested using Test 2. If Test 2 is reactive, the woman will be considered HIV infected and not eligible for enrolment. She will be counselled and referred to local health facilities for social support or other clinically indicated medical services. If Test 2 is non-reactive, a second sample will be taken and the above algorithm repeated on this second sample. If the results remain discordant, the woman is ineligible and should be counselled, receive confirmatory testing, and be referred to local health facilities for social support or other clinically indicated medical services as per local guidelines. *(NOTE: If the potential participant refuses a second sample at this visit, but is willing to return the next day, this is acceptable).*

STUDY VISITS

The participants will be tested using the same testing algorithm. If the HIV Test 1 is non-reactive, the participant will be considered uninfected and continue using the investigational product. If Test 1 is reactive, the participant will be tested using HIV Test 2. If Test 2 is reactive, the participant is considered to have been infected

while on the study, and should be permanently discontinued from the investigational product. Additional testing, including, but not limited to confirmatory Test 3 (a Western Blot), will be performed to confirm the result and obtain additional information on typing of the virus. The participant will be counselled and referred to local health facilities for social support or other medical services as clinically indicated.

If Test 2 is non-reactive, a second sample will be taken and the above algorithm repeated. If there is a repeat discordant result, the participant will continue intravaginal ring use and be asked to return for retesting after 2 weeks.

All participants continuing investigational product use will, in addition, have approximately 5 ml of blood taken at each study visit to be stored for possible HIV RNA PCR testing. If a participant subsequently seroconverts (i.e. is confirmed HIV positive) while on the investigational product, or if a PCR performed on the visit 6 sample is reactive, the stored samples will be tested in reverse sequential order until the PCR test result is negative. This will be done to identify the approximate period of infection.

Any participant who is confirmed HIV positive while on the study and is discontinued from investigational product may continue with all scheduled study visits and will have procedures performed as appropriate for safety. In addition, these women will be offered the option of rolling over into a seroconverter protocol.

EXIT VISIT
At the exit visit, 5ml of blood will be obtained by venipuncture for quantitative HIV RNA PCR testing to confirm the HIV-negative status of all participants who completed the study. If the PCR is positive, the stored samples will be tested in reverse sequential order until the PCR test result is negative.

NOTE: Up to 15% of all HIV rapid test samples will be retested at a central laboratory, for quality control purposes. Details of this testing will be provided in laboratory manual.
The laboratory manual will also provide details of the IPM specified Test 1; Test 2 and Test 3.

Participants who become infected with HIV during the course of an IPM-sponsored study will be referred for appropriate HIV-related care and ARV therapy. The threshold for initiation of ARV treatment will be determined with reference to the WHO treatment guidelines if no country specific guidelines are available. Women who become pregnant and HIV positive during the study will be provided with appropriate Prevention of Mother-to-Child Transmission (PMTCT) services.

The research centres will inform IPM Clinical Safety of any new HIV infections (seroconversions) within 24 hours of diagnosis. The applicable regulatory authorities and ethics committees who require expedited notification of HIV seroconversions will be notified by either IPM or the research centre in accordance with standard operating procedures and policies of the regulatory authorities or ethics committees.

5.4 Contraceptive Counselling

Participants will receive contraceptive counselling at screening and all study visits except Visit 6. Counselling will include reminders not to use intravaginal contraceptives (i.e., vaginal hormonal ring, diaphragm, cervical cap or shield) and reminders to continue on a stable oral contraceptive or long-acting progestin regimen, or IUD (unless participant has undergone surgical sterilization). Research centres will either directly provide contraceptives to the participants for the duration of the study or participants may continue to obtain contraceptives from their usual family planning facility.

Participants will also be counselled that if they do become pregnant during the study, they will immediately discontinue the investigational product and be referred to the local prenatal clinic for support services. See *Pregnancy Testing and Management* below in Section 5.5.

5.5 Pregnancy Testing and Management

A urine pregnancy test will be performed at all visits except Visit 5, at which time a serum pregnancy test will be performed. Urine pregnancy testing will also be provided at unscheduled visits in the event a participant misses her menses.

If a potential participant tests positive for pregnancy during screening, she is not eligible to enrol in the study but will receive referrals to prenatal clinics or other appropriate facilities.

If a participant tests positive for pregnancy while on study, she will remove the ring immediately (and permanently) and will be referred to a local prenatal clinic for support services. She will continue to be followed per the study schedule and will undergo procedures as indicated for safety. The research centres will be asked to report all pregnancies to IPM within 24hours of establishing diagnosis. The research centres will be asked to capture all data documenting the progress of the pregnancy and data from the first year of life of the infant for the sponsor maintained pregnancy registry.

5.6 Medical History and Physical Examination

At screening and Visit 1 (pre-enrolment), relevant medical history will be collected including but not limited to history of STIs, gynaecological conditions, hospitalizations, surgeries, allergies, any conditions requiring prescription or chronic medication, i.e., >2 weeks in duration, and acute conditions occurring prior to enrolment.

A general physical examination will also be conducted at screening and Visit 5 (week 12) which includes height, weight, vital signs, and examination of skin, respiratory, cardiovascular, central nervous and abdominal systems as well as an assessment of cervical and axillary lymph nodes.

5.7 Pelvic/Speculum Examination

A pelvic/speculum examination will be performed at screening and all 6 study visits (weeks 0, 2, 4, 8, 12, and 16 post enrolment). On-study examinations will be performed to assess safety, i.e., any local vaginal reactions.

The examination will include the following and must be performed by a suitably qualified person as determined by IPM and listed on the delegation of duties log:

- Naked eye examination of vulva
- Speculum examination of vagina and cervix
- Digital and bimanual examination for adnexal or fundal masses or tenderness
- Findings will be documented in the source documents and relevant CRFs

Each participant will be instructed to contact study staff in advance if menstruation is anticipated to occur on a visit with the pelvic/speculum examination. If the participant is menstruating, the entire study-visit should be rescheduled at least 2 days after menstruation is completed.

Any unexpected or abnormal vaginal bleeding should be investigated and the source identified. A follow-up pelvic/speculum examination with or without colposcopy (as dictated by the visit number or as clinically indicated), should be performed to ensure resolution of the condition and that no lesions were obscured by bleeding.

5.8 Intravaginal Ring Placement Check

Although a full pelvic/speculum examination will have been performed earlier, immediately following insertion of the intravaginal ring at each visit a brief digital examination will be performed by a qualified physician who is an IPM 015 investigator or a designated qualified nurse to verify that the ring has been properly placed by the participant.

At all post enrolment study visits (except Visit 6) when a pelvic/speculum examination is performed, the participant will remove the ring prior to the examination. At Visit 2, following the examination, the participant will rinse ring thoroughly with lukewarm water, if desired, and then reinsert the intravaginal ring. At Visits 3 and 4, she will insert a new intravaginal ring.

5.9 Colposcopy

Colposcopy will be performed by a suitably qualified colposcopist as determined by IPM and listed on the delegation of duties log on all participants at enrolment (Visit 1, Week 0) and study visits 3, 4, and 5 (Weeks 4, 8, and 12 respectively) in conjunction with the pelvic/speculum examination.

Colposcopy will be performed according to the WHO/CONRAD Manual for the Standardization of Colposcopy for the Evaluation of Vaginal Products (Update 2004), Revised Procedure for Colposcopy in the Development of New Vaginal Products.

Participants will be asked to abstain from the following 72 hours prior to the examination:

- Vaginal intercourse
- Oral contact with her genitalia
- Internal vaginal washing
- Penetration of the vagina by fingers, sex toys, or any other objects

At the time of the examination, any sexual activity reported within the previous 72 hours will be documented.

Both normal and abnormal findings will be documented during each procedure. Additional colposcopies may be performed if abnormalities or lesions are identified or until symptom resolution or at the discretion of the investigator based on signs or symptoms.

5.10 Laboratory Testing

At the screening visit and Visits 3, 4, and 5 (Weeks 4, 8, and 12 respectively), all participants will have safety laboratory testing including urine obtained for urinalysis (dipstick, and microscopy as indicated) and blood drawn (approximately 10ml or 2 teaspoons) by venipuncture for laboratory testing (haematology, chemistry, renal and liver function). Haematology will include full blood counts, differential, and platelet count. Chemistry and renal/liver function tests will include electrolytes (sodium, potassium, chloride, phosphate and calcium), BUN, creatinine, bilirubin, AST (SGOT), ALT (SGPT), ALP, and GGT.

5.11 STI Testing

Cervico-vaginal samples will be collected for STI testing at screening and at visits 3, 4, and 5 (Weeks 4, 8, and 12 respectively). All participants will be evaluated for bacterial vaginosis (BV), Trichomonas vaginalis (TV), Neisseria gonorrhoea (NG), and Chlamydia trachomatis (CT).

The following tests and procedures will be performed for the associated STIs:

**STI**	**Laboratory Test**
Bacterial Vaginosis	• Wet Mount<br>• Vaginal pH<br>• Whiff Test<br>• Homogenous Vaginal Discharge<br>***NOTE:*** *Tests will be done according to Full Amsel criteria*
Trichomonas	• InPouch culture
Gonorrhoea	• *Neisseria gonorrhoeae* Nucleic Acid Test (NG NAT)
Chlamydia	• *Chlamydia trachomatis* Nucleic Acid Test (CT NAT)

At screening, approximately 5ml (1 teaspoon) of blood will be collected by venipuncture for Syphilis (RPR) testing.

Other tests may be performed at the investigator's discretion after discussion with the IPM Clinical Safety Physician (or designee) based on symptomatology and clinical assessment. See separate *Laboratory Manual* for additional descriptive information regarding specimen collection and processing for all tests.

All laboratory results should be reviewed by a qualified physician that is an IPM 015 investigator with the review documented on the original laboratory report itself.

<u>STI Treatment</u>
Participants will be treated at the study research centre or referred to a local health facility for per local STI Treatment Guidelines. Those with non-curable STIs, e.g.,

HSV-2 or HPV, will be referred to a local health facility for treatment per local STI Treatment Guidelines. See *Clinical Management of Genital Diagnoses* (Please see the Study Operations Manual) for guidelines to determine whether intravaginal ring requires temporary or permanent removal and follow-up recommendations.

5.12 Vaginal Flora and Vaginal pH Testing

At enrolment prior to initial ring insertion, and at visits 3, 4 and 5 (weeks 4, 8 and 12) after ring removal, cervicovaginal specimens will be taken for Gram stain to determine vaginal flora. Vaginal pH will be recorded. Details of the specimens to be taken and the laboratory tests to be performed will be provided in the *Study Operations Manual* and the *Laboratory Manual* which will be supplied by the laboratory.

5.13 Pap Smear

A Pap smear will be done at screening. Any potential participant with a Pap smear result that requires cryotherapy, biopsy, treatment (other than for infection), or further evaluation including any findings of atypical squamous cells of undetermined significance (ASCUS) is not eligible to enrol in the study but should be referred for medical services as clinically indicated.

5.14 Intravaginal Ring Insertion & Removal

At the Visits 1, 2, 3, and 4, when the intravaginal ring is inserted under clinic supervision, the participant will be instructed to wash her hands thoroughly, relax, and get into a comfortable position, either standing with one foot on a chair or lying on her back with her knees up (see Appendix B). After opening the folds of skin around the vagina, she will gently squeeze the ring into an oval shape and push it upwards and backwards towards the small of the back as far as it will go. She will then be instructed to wash her hands thoroughly again. A brief digital examination will be performed by a qualified physician who is an IPM 015 investigator or a designated qualified nurse immediately after to verify proper placement of the ring If upon digital examination the ring is not inserted correctly, the investigator or nurse should allow the participant a maximum of 3 attempts to re-insert the ring properly or provide assistance as required to put the ring in place.

At Visits 2, 3, 4, and 5 when the intravaginal ring is removed, under clinic supervision, prior to the pelvic exam, the participant will be instructed to wash her hands thoroughly, relax, and get into a comfortable position, either standing with one foot on a chair or lying on her back with her knees up. She will be instructed to put her finger into her vagina, hook it around the ring, and gently pull downwards and forwards. She will then be instructed to wash her hands thoroughly again. The investigator or nurse should allow the participant a maximum of 3 attempts to remove the ring properly or provide assistance as required to remove the ring.

If, at any time (including scheduled and unscheduled visits), the participant requests help with either removal or re-insertion of the intravaginal ring, or after she has made a maximum of 3 attempts to remove/re-insert the ring without success, trained study staff may give assistance. Re-education of the participant on ring removal/re-insertion should be given. This should be noted in the source documents and applicable CRFs.

At Visits 3, 4 (when a new ring is provided to the participant) and 5 (when the ring is permanently removed), the used intravaginal ring will be sent to an analytical laboratory for residual dapivirine testing.

5.15 Intravaginal Ring Adherence Counselling

At visits 1 through 4 participants will receive intravaginal ring adherence counselling at the time of ring insertion and at every follow-up visit until final ring removal. Research centre staff will counsel participants to refrain from removing the ring (except as directed during clinic visits) and from using concomitant vaginal products or other objects. Research centre staff will also provide instructions for re-insertion in case of accidental ring expulsion, e.g., during sex or exercise.

If, for any reason, the participant is non-compliant in her use of the intravaginal ring (i.e. she removes the ring for any purpose other than as instructed at a study visit), this should be documented in the source documents and applicable CRFs and an attempt made to establish the reason for non-compliance.

5.16 Assessments

5.16.1 Adherence Assessments

During the study, trained staff will administer a confidential adherence assessment at Visits 2, 3, 4 & 5.

5.16.2 Acceptability Assessments

During the study, trained staff will administer a confidential acceptability assessment at Visits 1, 3 & 5.

5.16.3 Behavioural Assessments

Behavioural assessments will be conducted at Visits 1, 3, 4, 5 & 6 including sexual behaviour, intravaginal practices and condom use.

5.16.4 Diary Card

Participants will be provided with a diary card which will incorporate product use, sexual acts, condom use, appointment dates and investigator information at screening and it will be reviewed at Visits 1, 2, 3, 4 and 5.

5.17 Dapivirine Levels

Blood will be drawn (approximately 10mls) by means of venipuncture for measurement of plasma dapivirine levels at visits 1, 3 & 5. Residual dapivirine levels will be measured in the returned used / unused rings.

5.18 Method of Treatment Assignment

Participants who meet all of the study inclusion criteria and no exclusion criterion at baseline will be randomly assigned in a 1:1 ratio to one of two groups receiving either the intravaginal ring containing dapivirine or the placebo intravaginal ring

with no dapivirine. Both groups will use a 28 days administered ring continuously for 12 weeks and have a follow-up visit 4 weeks post ring removal.

A master randomization list for the study will be generated which links each sequential PID number to a study treatment assignment (dapivirine ring or placebo ring). At each study research centre, as each new participant enters the study, a sequential PID number will be assigned to that participant using an Interactive Voice Response System (IVRS), starting with the lowest number available at that time. No PID numbers should be skipped or repeated.

Participants will be provided with an appointment card detailing investigator name, address and phone number and the instructions to keep the card in their possession at all times.

5.19 Reimbursement

Participants will be reimbursed for their time, effort and any travel costs incurred as per local regulations. Reimbursements will be made after the completion of each scheduled study visit. Research centre specific reimbursement amounts will be documented in the study informed consent approved by the applicable IRB/IEC.

5.20 Study Operations Manual

A separate Study Operations Manual will be supplied to all research centres to provide general guidance on the conduct of study procedures.

6.0 INVESTIGATIONAL PRODUCT

6.1 Investigational Product Composition

The dapivirine intravaginal matrix ring is an off-white flexible ring containing 25mg of drug substance dispersed in a platinum-catalyzed cured silicone matrix. The dimensions of the ring are 56mm and 7.7mm, outer diameter and cross sectional diameter, respectively. The dapivirine intravaginal ring is designed to provide sustained release over a 28-day period.

The placebo ring composition is the same as the dapivirine ring with the exception of the absence of dapivirine, and inclusion of titanium dioxide USP colorant. Pharmacopoeial grade titanium dioxide is included as a colorant to maintain blinded conditions during clinical evaluation. Details regarding the composition of the dapivirine and placebo rings are included in the IB.

The safety of silicone intravaginal rings as drug delivery devices has been well established through extensive clinical experience of marketed products. For example, Pfizer (formerly Pharmacia and Upjohn Company) has marketed Estring® (estradiol intravaginal ring), an intravaginal ring that is also made from silicone elastomer and contains estradiol used to treat local symptoms of urogenital atrophy, since 1993. Prior to the launch of Estring®, the biological safety of the silicone elastomer was studied in various *in vitro* and *in vivo* test models. The results show that the silicone elastomer is non-toxic, non-pyrogenic, non-irritating, and non-sensitizing (13).

The Dapivirine Ring-004 is comprised of dapivirine, the polydimethylsiloxane liquid MED-360 and the silicone MED-4870. The safety of dapivirine has been

established in a comprehensive nonclinical and clinical development programme described in the IB. Both the silicone elastomers and the liquid silicone dispersant have been evaluated in *in vitro* cytotoxicity, haemolysis tests, cytogenic damage and genotoxicity assays, and in *in vivo* systemic toxicity studies, intracutaneous toxicity studies, pyrogen studies and delayed contact sensitization studies. The silicone elastomer was also evaluated in muscle implantation studies of up to 12 weeks duration. In addition, biocompatibility tests on the finished Ring-004, including in vitro cytotoxicity and genotoxicity assays, and in vivo vaginal irritation and delayed contact sensitization studies, have also been performed. None of these tests identified any significant safety concerns.

6.2 Packaging and Labelling

The placebo and investigational intravaginal rings will be packed individually per participant. IPM will bear the responsibility for primary and secondary packaging and labelling. The packaged rings will be labelled according to local regulatory requirements. At a minimum, the secondary packaging label will include the following information:

- IPM 015
- Dapivirine intravaginal matrix ring or placebo intravaginal ring
- Medication Number
- Expiry date
- Storage conditions
- For clinical study use only
- For vaginal use only
- Keep out of reach of children

6.3 Randomization

A blocked randomization schedule will be generated and validated according to specifications required for IPM's process of packaging and dispensing. The block sizes will not be disclosed until the conclusion of the study. The schedule will contain sequential participant identification numbers (PID) in consecutive order and treatment assignment. At each research centre, each enrolled participant will be assigned a PID in consecutive order using an Interactive Voice Response System (IVRS) starting with the lowest number available at the time

6.4 Blinding and Unblinding

The Principal Investigator or his/her designee will be able to break the code of each successfully enrolled participant, if necessary. If during the course of the study a medical emergency requires knowledge of the test agent used by a particular participant, the study blind or code may be broken for that specific participant after discussion with the IPM's Clinical Safety Physician whenever possible. Any broken blind must be justified and explained in the source documents and applicable CRF and reporting forms. The unblinding as well as the reason for unblinding must be documented in the source documents. If the code is broken by the Principal Investigator or his/her designee, the participant must be withdrawn from the study and must be followed up if appropriate. . The blinding & unblinding process will be co-ordinated by the Interactive Voice Response System (IVRS).

6.5 Investigational Product Storage

The recommended storage condition for the dapivirine and placebo rings is 15°C to 30°C. The product should not be frozen. In the event that the investigational product has been subjected to different storage conditions than specified above, the affected investigational product must not be used (unless IPM or its designee provides written authorization for use). IPM should be notified immediately.

The Investigator (or pharmacist) will maintain an inventory and acknowledge receipt of all shipments of investigational product.

6.6 Investigational Product Administration

Participants will self-insert a new intravaginal ring at enrolment (Visit 1) and weeks 4 and 8 (Visit 3 and 4). A follow-up visit 4 weeks post ring removal will also be conducted. Participants should continue ring use through menses, however not using study product during menses will not be considered a protocol deviation or violation, but will be documented on the source document and applicable CRFs.

6.7 Investigational Product Expulsion or Loss

If the participant accidentally expels the ring, e.g., during sex or exercise, she should be instructed to rinse the ring thoroughly in lukewarm water and re-insert the intravaginal ring. If an IVR is expelled and can not be successfully reinserted, the ring should be appropriately rinsed, lightly blotted dry, and stored in a clean place, preferably between 15-30°C, until earliest possible opportunity for reinsertion at the clinic. Transport to the clinic can be achieved in a small bag or similar container, but effort should be made to minimize handling.

If the ring is expelled in such a manner that the participant is unwilling to re-insert the ring, e.g., during urination or a bowel movement, or if the ring is lost, the participant should be instructed to return to the clinic.

Management of this situation should be on a case by case basis following discussion by the Investigator with the IPM Clinical Safety Physician or designee.

6.8 Investigational Product Accountability

The Principal Investigator or designee will be responsible for adequate and accurate accounting, handling, storage and dispensing of the investigational product. The investigational product will be stored safely and properly in a secure location with access available only to the Principal Investigator and designated study personnel. Investigational product and clinical supplies are to be dispensed only in accordance with the protocol. Accurate records of the investigational product received from IPM, the amount dispensed to the participants, the amount returned by the participants, the quantity remaining at the conclusion of the study and any wasted or expired investigational product must be maintained.

At the conclusion of the study, the returned used and or unused rings will be sent to an analytical laboratory for measurement of dapivirine levels.

6.9 Concomitant Medications & Products

All prescription and non-prescription medications, including any treatment for STIs and other vaginal infections, will be collected and recorded on the source documents and applicable CRFs.

Concomitant use of vaginal products or other objects including tampons, female condoms, cotton wool, rags, diaphragms, cervical caps, douches, and drying agents are prohibited for the duration of the study. *NOTE: If any of these products are used, this will be considered a protocol deviation and will be documented on the source document and applicable CRFs.*

7.0 ADVERSE EVENTS

7.1 Definition

An adverse event (AE) is any untoward medical occurrence during the course of a study in a participant who received investigational product at any dose and that does not necessarily have a causal relationship with the investigational product. An AE can therefore be any unfavourable and unintended sign (including an abnormal laboratory finding), symptom or disease temporally associated with the use of an investigational product, whether or not considered related to the investigational product. This definition includes intercurrent illnesses or injuries and exacerbation of pre-existing conditions.

An *unexpected adverse event* is an adverse reaction, the nature or severity of which is not consistent with the applicable product information (e.g., IB for an unapproved investigational medicinal product). Final determination of whether an event is considered unexpected will be made by IPM, but the Investigator should be knowledgeable of the contents of the IB.

Whenever possible, the laboratory abnormalities should be considered in the context of the primary clinical diagnosis and reported as such (e.g., acute hepatitis with increased bilirubin). Laboratory abnormalities will be considered AEs and graded for severity based on the *Division of AIDS (DAIDS) Table for Grading Adverse Events*

Any condition occurring prior to enrolment (treatment assignment) at Visit 1 will be reported as a pre-existing condition under Medical History. <u>All AEs occurring during the study will be recorded in the source documents and applicable CRFs and report forms</u>.

If possible, a specific disease or syndrome rather than individual associated signs and symptoms should be recorded by the investigator. However, if an observed or reported sign, symptom, or clinically significant laboratory abnormality is not considered a component of a specific disease or syndrome by the investigator, it should be recorded as a separate AE.

All AEs should be monitored until resolution and/or the cause is identified or until the investigator does not expect any improvement or worsening of condition/symptoms. If an AE remains unresolved at the participant's last study visit, the research centre investigator will make a clinical assessment with the IPM

Clinical Safety Physician to determine whether continued follow up of the AE is warranted.

7.2 Assessment of Adverse Event Severity

The investigator is responsible for assessing the severity of adverse events occurring on study. All AEs except genital complaints will be graded according to the *DAIDS Table for Grading Severity of Adult and Pediatric Adverse Events (*Available at http://rcc.tech-res.com/tox_tables.htm). All genital complaints will be graded according to the *Female Genital Grading Table for Use in Microbicide Studies* (Available at http://rcc.tech-res.com/eae.htm) which will be provided to research centres in the Study Operations Manual.

For AEs not listed on either of these tables, the following criteria will be used to estimate the grade of severity:

- **Mild**

  Transient or mild discomfort (<48 hours); no medical intervention/therapy required

- **Moderate**

  Mild to moderate limitation in activity – some assistance may be needed; no or minimal medical intervention/therapy required

- **Severe**

  Marked limitation in activity, some assistance usually required; medical intervention/therapy required; hospitalizations possible

- **Life-threatening**

  Extreme limitation in activity, significant assistance required; significant medical intervention/therapy required; hospitalization or hospice care probable

7.3 Relationship to Investigational Product

The investigator is responsible for determining the relationship of all AEs occurring on study and will assess AEs based on the following criteria:

- **Not Related**

  There is not a temporal or causal relationship to the investigational product administration. The AE is clearly explained by another cause (concurrent disease, concomitant medication, environmental or toxic factors, etc.).

- **Probably Not Related**

  There is a temporal relationship to investigational product administration, but there is not a reasonable causal relationship between the investigational product and the event The AE is more likely explained by another cause (concurrent disease, concomitant medication, environmental or toxic factors, etc.).

- **Possibly Related**

  There is a reasonable causal relationship between the investigational product and the AE. The AE is equally likely explained by another cause

but the possibility of the investigational product relationship cannot be ruled out, e.g., the administration of the investigational product and AE are considered reasonably related in time.

- **Probably Related**

  There is a reasonable temporal and causal relationship between the investigational product and the AE. The AE is more likely explained by the investigational product, e.g., the administration of the investigational product and AE are considered reasonably related in time and the AE is less likely explained by another cause.

- **Definitely Related**

  There is a reasonable causal relationship between the investigational product and the AE, when the event responds to withdrawal of the investigational product (dechallenge), and recurs with rechallenge by administration of the investigational product. The AE is clearly related and most likely explained by the administration of the investigational product.

7.4 Serious Adverse Events

7.4.1 Serious Adverse Event Definition

A Serious Adverse Event (SAE) is defined as any untoward medical occurrence that at any dose meets any of the following criteria:

- Results in death
- Is life-threatening

  This criterion applies if the participant is at immediate risk of death from the event as it occurred, in the opinion of the investigator; it does not refer to an event which hypothetically might have caused death if it were more severe.

- Requires inpatient hospitalization or prolongation of existing hospitalization

  This criterion applies if the event requires inpatient hospitalization and results in an overnight stay in hospital or, if in the opinion of the investigator, prolongs an existing hospitalization. A hospitalization (including hospitalization for an elective procedure or routinely scheduled treatment) for a pre-existing condition which has not worsened does not constitute an SAE.

- Results in persistent or significant disability/incapacity

  This criterion applies if the event causes a substantial disruption of a person’s ability to conduct normal life functions.

- Is a congenital anomaly/birth defect

  This criterion applies if a participant gives birth to a child with a congenital anomaly or birth defect.

- Is an important and significant medical event that may not be immediately life threatening or result in death or hospitalization but, based upon appropriate medical judgment, may jeopardize the participant or require intervention to prevent one of the other outcomes listed above. e.g., bronchospasm requiring intensive treatment in an emergency room or at home.

NOTE: *An SAE need not be severe in nature to meet any of the above criteria.*

All SAEs that occur from the time the participant is enrolled (receives treatment assignment) through the duration of the study, whether considered to be associated with investigational product or not, must be reported to the IPM Clinical Safety Physician or designee within 24 hours of the research centre becoming aware of the event. All SAEs should be reported using the designated IRE Report Form.

The IRE Report Form should be completed with all available information at the time of reporting. The investigator is required to write a detailed written report and complete SAE follow-up in a timely manner until the SAE returns to baseline, participant returns to normal health or until the investigator does not expect further improvement or worsening of the event. Medical records may be requested by IPM to assist in assessing relatedness and severity of the SAE, and for possible submission to Regulatory or Health authorities. To maintain confidentiality, the participant's name must be blacked out and replaced with the Participant Identification Number and initials on any medical records submitted.

More details on SAE reporting requirements are described in separate Safety Reporting Plan.

7.4.2 Serious Adverse Event Contact Information

Serious Adverse Events will be sent to IPM within 24 hours of the research centre becoming aware of the event.

If the SAE is related, and life-threatening or fatal, immediately telephone the CSP.

CONTACT INFORMATION

Primary Clinical Safety Physician	Mercy Kamupira, MBChB, or designee
Office Phone:	+27 21 860 2300
Mobile Phone:	+27 71 604 8077
Safety Reporting Email	safetyreports@ipm-microbicides.org
Office Fax:	+27 21 860 2308
Office Email:	mkamupira@ipm-microbicides.org.za

IPM will process all safety events. The Clinical Safety Physician will review all SAEs and generate the necessary queries.

7.4.3 Sponsor Notification of SAEs to Regulatory Agencies

All SAEs will be reported according to the guidelines of the local ethics and regulatory agencies in the countries in which IPM studies are being conducted. Any unexpected serious adverse event which is deemed to be "Definitely Related", "Probably Related", or "Possibly Related" to the investigational product will be considered "associated with the use of the investigational product" and thus IPM will notify appropriate regulatory authorities of the event in an expedited manner

unless policies of local regulatory authorities mandate such reporting by the study research centres.

Any unexpected serious adverse event deemed to be “Probably Not Related” or “Not Related” will not be reported to regulatory authorities in an expedited manner unless otherwise requested by the local authorities.

7.4.4 Research centre Notification of SAEs to Local Ethics Committee or Local Health or Regulatory Authorities

The investigator will report all SAEs to the local Ethics Committee (EC) and/or health or regulatory authorities in accordance with standard operating procedures and policies of the EC and/or health or regulatory authorities.

7.5 Immediately Reportable Events

In addition to the SAEs the following events will be considered Immediately Reportable events (IRE) and will be reported to IPM within 24 hours:

- Pregnancy: Although not considered an adverse event, pregnancy must be reported if it occurs at any time during the study;
- HIV infection any time during the study;
- Any non-serious adverse event leading to permanent discontinuation of the investigational product (including laboratory abnormalities).

7.6 Safety Monitoring

Safety data from the study will be evaluated at predetermined regular intervals by an independent Safety Evaluation Committee (SEC). The SEC has the option of stopping the study at any point, if warranted, based on adverse events observed during the study or other concerns regarding participant safety or study conduct.

Close collaboration among SEC members will be necessary to evaluate study progress and respond to occurrences of toxicity in a timely manner. Rates of accrual, study product compliance, follow-up, and AE incidence will be monitored by the SEC on a regular basis. The SEC will meet via conference call approximately every six weeks during the study; ad hoc calls may be convened if requested by the SEC or IPM. A charter detailing the members of the SEC and the order of proceedings will be provided in a separate document.

8.0 DATA MANAGEMENT

8.1 Data Handling at Study Research centres

All study data will first be collected on designated source documents and then recorded on Case Report Forms (CRFs) with the exception of the Behavioural Acceptability and Adherence Questionnaires, for which the CRFs will serve as the source unless otherwise specified by IPM. Research centre staff responsible for completing the CRFs will receive proper training prior to study start and will follow standardized procedures. Data must be legibly entered onto the CRFs. Data corrections will be made in accordance with standard procedures provided by IPM or its designee. Instructions for CRF completion will be provided in the Study Operations Manual.

The investigator will maintain, and store in a secure manner, complete, accurate and current study records throughout the study. Standard GCP practices will be followed to ensure accurate, reliable and consistent data collection.

8.2 Source Data Verification

All study data must be verifiable to the source documentation (which includes original recordings, laboratory requisitions and reports, medical records, etc.). Source documentation will be available to the Sponsor or representative(s) for review to ensure that the collected data is consistent with the CRFs and has been completely and accurately reported as required by the study protocol.

9.0 STATISTICAL METHODS

9.1 General Design

IPM 015 is a double-blind, randomized (1:1 allocation), placebo-controlled phase I/II study to evaluate the safety of an intravaginal ring with dapivirine in healthy HIV-negative women.

All enrolled participants will self-insert an intravaginal ring (either placebo or containing dapivirine) at enrolment (Visit 1) to be worn for 12 continuous weeks with ring replacements at weeks 4 and 8 (Visits 3 and 4). Participants will be asked to return to the clinic for follow-up visits 2, 4, 8, and 12 weeks post enrolment (Visits 2-5) to monitor safety. A follow-up visit 4 weeks post ring removal will also be conducted at week 16 (Visit 6). In addition to evaluating safety, the two study arms will also be compared with regard to the effect on vaginal flora and vaginal pH. Additionally, sexual behaviour, vaginal practices and condom use pre-randomization and during follow-up will be monitored, ring acceptability will be assessed at stud entry and exit and ring use and adherence will be assessed at each follow up visit.

9.2 Endpoints and Assessments

9.2.1 The primary endpoints are:

- The proportion of women with cervico-vaginal mucosal abnormalities (as defined in the WHO/CONRAD manual) visible by naked eye examination and/or colposcopy.
- The proportion of women with at least one adverse event during the 16-week study period.
- The proportion of women with any laboratory abnormalities on haematology, liver function, and renal function.
- The proportion of women with positive diagnostic tests for trichomonas, gonorrhoea, and chlamydia.
- The proportion of women with abnormal vaginal flora and/or pH at any point during the study.

9.2.2 The primary endpoints will be assessed through:

- Gynaecological assessments, including pelvic/speculum exam and colposcopy, and laboratory STI testing.
- Safety laboratory blood tests.

- Adverse event/serious adverse event reports.
- Vaginal flora analysis and vaginal pH measurement.

9.2.3 The secondary endpoints are:

- The proportion of women who find the use of the intravaginal ring acceptable.
- The proportion of women who are adherent to the use of the intravaginal ring inserted once every 28 days over the full 12-week period.

9.2.4 The secondary endpoints will be assessed by:

- Questionnaires regarding sexual behaviour, acceptability and adherence to the use of an intravaginal ring inserted once every 28 days.
- Self-reported diary of intravaginal ring use,.
- Clinician assessment of the intravaginal ring in situ at each study visit.

9.2.5 The exploratory objectives will be addressed as follows:

Study data, including demographic characteristics, questionnaires on acceptability and adherence, questionnaires on sexual behaviour, and other self reported information including diary cards will be used in exploratory models as possible predictors of adherence and non-adherence to 28 day intravaginal ring use. Other exploratory models will investigate the degree of agreement between levels of dapivirine and/or residual levels of dapivirine in returned rings and participant self reports of adherence to develop potential methods for monitoring adherence in larger studies.

9.2.6 The exploratory objectives will be assessed by:

- Questionnaire concerning factors influencing adherence and non-adherence to 28 day intravaginal ring use.
- Questionnaires regarding sexual behaviour, acceptability and adherence to the use of an intravaginal ring inserted once every 28 days.
- Self-reported diary of intravaginal ring use, sexual behaviour and condom use.
- Plasma dapivirine levels obtained at predetermined time points during the 12-week treatment period.
- Residual dapivirine levels measured in the returned used or unused rings.

9.3 Sample Size

Approximately 280 sexually active women (15 - 40 per research centre) will be enrolled with competitive enrolment. If a research centre is unable to complete enrolment within 7 months of the SIV, enrolment at the research centre for this study may be ended.

The Sponsor is aware that it would be difficult to detect a low rate (less than 1 in a thousand) adverse event. Heterogeneity between research centres might affect the outcomes and estimates made for IPM015. This study is designed to be exploratory in nature.

9.4 Analyses

The analysis will focus on safety measures, with additional assessments evaluating acceptability and adherence to 28 day use of the intravaginal ring, changes in vaginal flora and vaginal pH throughout the study as compared between the two treatment arms, possible predictors of adherence (including sexual behaviour and condom use of women using an intravaginal ring), as well as plasma dapivirine levels and residual dapivirine levels in returned used or unused rings. The primary analysis will be conducted on the intent-to-treat population, with some analyses conducted on the per-protocol population or subgroups of particular interest, as appropriate.

Details on the definition of the per-protocol population, models that will be used to examine the exploratory objectives, as well as other technical aspects of these analyses will be written in a Statistical Analysis Plan that will be finalized prior to unblinding of study data.

Simple, appropriate descriptive analyses will be generated for selected variables. For continuous variables, they will include, but will not be limited to overall mean and standard deviation (when appropriate), mean by study group, mean by research centre, median and percentiles, range, and number of missing data points. For categorical variables they will include overall frequencies (when appropriate), frequencies by study group, and frequency listing by research centre. This section discusses the planned analyses beyond the listings and simple descriptive analyses.

9.4.1 Analysis of Safety

The primary objective of this study is the assessment of the safety of the dapivirine ring.

This objective will be evaluated using clinical and laboratory examinations as well as self-reported adverse events. Data will be collected through pelvic/speculum examinations and colposcopic observations performed during specified study visits, (see Appendix A for scheduled clinical procedures and laboratory tests), reported adverse events, and laboratory testing.

The two ring treatment groups will be compared to assess the safety of the dapivirine matrix ring. As this is a randomized study, it is anticipated that the two groups will be comparable at baseline with respect to pre-existing conditions, and furthermore, that women with pre-existing conditions will be treated and evaluated before being enrolled into the study. For this reason, no baseline adjustments are planned prospectively, but the data will be thoroughly reviewed to assess any potentially relevant baseline imbalances.

Adverse events and abnormal observations during the gynaecological examinations will also be compared to other data collected in the study, including STI and HIV test results, since detected genital abnormalities may also be caused by medical conditions. Data on sexual practices, vaginal practices, and adherence to 28 day ring use will also be explored in relation to adverse events and abnormal observations during gynaecological examinations to identify possible safety hypotheses that may be tested in a larger Phase III study.

9.4.2 Analysis of Acceptability

The analysis of acceptability is intended to understand the factors that influence participant's acceptance of the product. Acceptability of the ring will be assessed using the variables selected from the following group of variables of the Acceptability Assessment: Willingness to Use and Product Use Experience. The acceptability assessments will be administered to enrolled participants at enrolment, prior to randomization, and visits 3 & 5. Group differences between women using the dapivirine ring and those women using the placebo ring will be evaluated at study end.

The study's primary measure of acceptability will be defined as the proportion of women who find the use of the intravaginal ring acceptable as determined by self-reports during the study.

Data on sexual practices, vaginal practices, adherence to 28 day use of the ring, and product use experience will also be explored in relation to acceptability to identify possible hypotheses that may be tested in a larger Phase III study.

Data collected in the Product Use Experience sections of the Acceptability Assessment and the Adherence Assessment will be important for understanding both the product's acceptability and study regimen adherence. Proportions of women who respond favourably to each of the questions at each assessment administration time point will be calculated for each study group, research centre, as well as two study groups combined. It is not expected that the random assignment will influence women's experience with the product.

The acceptability data will also be explored in conjunction with other study data to investigate factors that may influence the participants' attitudes and predict participant responses to the study product in future studies.

9.4.3 Analysis of Adherence

Adherence data will be analyzed in multiple ways in this study, both as a secondary endpoint as well as an exploratory objective, through models that aim to understand the factors that influence adherence behaviour. To this end, as a secondary endpoint, the proportion of women who reported using the ring for the full twelve-week study period will be calculated for each study group and research centre, cumulatively at visits 2, 3, 4 & 5. Adherence will be assessed through self-reported diary & data collected by interviewer-administered assessment and by clinician's assessment of ring's presence in situ at each visit.

Additionally, as exploratory objectives, multivariate regression models will be used to identify potential predictors of adherence, using as covariates sexual practice, vaginal practice, and other participant-specific characteristics. Other exploratory analyses of adherence data will use quantitative measures of plasma levels of dapivirine and/or residual levels of dapivirine in returned rings and will examine the degree of agreement between participant and clinician report of adherence versus these quantitative measures. These exploratory analyses will be used to develop potential methods for monitoring and encouraging adherence to use of study product in studies involving a larger number of participants and a longer duration of product use.

9.5 Interim Analysis

An interim analysis will be conducted after approximately 70 participants have completed 12 weeks of ring use. This analysis will concentrate on the safety assessment data.

9.6 Handling of Missing Data and Dropouts

Some degree of missing data, primarily associated with missed visits, is expected. The amount of missing data will be explored and incorporated into the analyses, where appropriate. For the analysis of adherence to 28 day ring use, patterns of missing data may be informative. Depending on the proportion of participants who discontinue early, an analysis of time to discontinuation may be informative. Such an analysis would allow investigation of the covariates associated with early discontinuation and could provide information that would be useful in designing future studies of the microbicide ring.

10.0 INVESTIGATOR REQUIREMENTS

10.1 Study Initiation

The study cannot be initiated at the research centre until the research centre has been fully qualified with the Sponsor. Following Sponsor approval, IPM will notify the research centre in writing via letter correspondence to begin study operations according to the protocol and all other related study materials.

Prior to implementation, the following documents must be on file with IPM or its representative:

- Protocol signature page signed and dated by the Principal Investigator
- Investigator Brochure signature page signed and dated by the Principal Investigator
- Pre-study visit report
- Research centre Counselling Checklist signed & dated by IPM or its representative
- Statement of Investigator signed and dated by the Principal Investigator
- All Co-Investigators must be listed on the Statement of Investigator, or local equivalent. Investigators must also complete all regulatory documentation as required by local and national regulations
- Current signed and dated curricula vitae of the Principal Investigator and all Co-Investigators which includes medical licensure and/or medical qualifications and cites the association that the Principal Investigator and Co-Investigators have with the research centre.
- Current signed and dated curricula vitae of the Laboratory Director or designee
- Signed and dated Financial Disclosure Forms for Principal Investigator and all Co-Investigators listed on the Statement of Investigator
- Institutional Review Board and/or Independent Ethics Committee (IRB/IEC) membership list
- Written documentation of IRB/IEC and/or other national ethics committee (if applicable) approval of protocol and informed consent document (both identified by study protocol number or title and date of approval)
- Copy of the IRB/IEC-approved informed consent document

- Written documentation of IRB/IEC review and approval of any advertising materials to be used for study recruitment as well as participant information
- Current laboratory certification of the laboratory performing the analysis (if available), as well as current normal laboratory ranges for all laboratory tests
- Signed Clinical Study Agreement
- Certified translations and back-translations of approved informed consent document, and pertinent correspondence (when applicable)
- Other country-specific required documents including regulatory authority approval or acknowledgement of receipt of notification

All regulatory and ethics committee(s) submissions need to be reviewed and approved by IPM prior to submission to the applicable agencies.

10.2 Institutional Review Board or Independent Ethics Committee Approval

This protocol, the informed consent document, and relevant supporting information must be submitted to the IRBs/IECs for review and must be approved before the study is initiated.

The Principal Investigator is responsible for communicating with IRBs/IECs regarding the progress of the study and changes made to the protocol as deemed appropriate, but in any case at least once a year. The Principal Investigator must also keep the IRBs/IECs informed of any significant adverse events and SAEs.

10.3 Study Monitoring and Audits

Study monitors will regularly visit participating study research centres to review all study documents including but not limited to individual participant records, consent forms, source documents, CRFs, supporting data, laboratory specimen records and medical records (physicians' progress notes, nurses' notes, individuals' hospital charts) to ensure protection of study participants, compliance with the protocol, and accuracy and completeness of records. The study monitors also will inspect the research centre's regulatory files to ensure that regulatory requirements are being followed; the research centre's pharmacies to review product storage, management, and drug accountability; and the research centre's laboratory and other clinical supplies to ensure proper storage and continued viability of supplies. All applicable study documents should be readily available for review during the visits. The study monitors will also check that clinical study procedures are observed and will discuss any problems with investigator or designee as applicable.

During or after the clinical study, the governmental regulatory authorities, local IRB/IEC and/or representatives of the Sponsor may request access to all study documents for on-research centre audit or inspection.

10.4 Case Report Forms

Case Report Forms (CRFs) will be supplied by IPM or its designee and should be handled in accordance with instructions from IPM.

All CRFs should be filled out completely by the designated study staff. Upon study completion, the CRF is reviewed, signed, and dated by an investigator listed on the Statement of Investigator.

All CRFs should be completed in a neat, legible manner to ensure accurate interpretation of data. Black ink is required to ensure clarity. When making changes or corrections, the original entry should be crossed out with a single line, and the change initialled and dated. Erasures, overwriting, and correction fluid are NOT allowed on the CRFs.

10.5 Disclosure of Data

Participant medical information is confidential and disclosure to third parties other than those described in Section 10.3 is strictly prohibited. All study data will be stored securely at the study research centre. All participant information including laboratory reports, forms, lists, logbooks, appointment books and administrative forms will be stored in locked file cabinets in areas with access limited to study staff.

Participants' study information will not be released without written permission of the participant, except as necessary for monitoring by the Sponsor, Sponsor's designated monitors, or regulatory authorities.

10.6 Record Retention

The Principal Investigator will retain in a secure manner, complete, accurate and current study records for a minimum of two years after marketing approval or termination of product development. Study records include administrative documentation, including research centre registration documents and all reports and correspondence relating to the study, as well as documentation related to each participant screened and/or enrolled in the study, including informed consent forms, CRFs, notations of all contacts with the participant, and all other source documents. All records must be retained on-site throughout the study's period of performance. The Sponsor will provide the study research centre with written instructions for long-term record storage at the completion of the study.

No records should be destroyed without prior written permission from IPM.

11.0 ETHICAL CONSIDERATIONS

11.1 Ethical Review

This protocol, research centre-specific informed consent forms, participant education, outreach, recruitment materials and any other requested documents or subsequent modifications will be reviewed and approved by the ethical review bodies responsible for oversight of research conducted at the study research centre.

Subsequent to initial review and approval, the local Institutional Review Board (IRB) and/or Independent Ethics Committee (IEC) will be notified about study completion within three months following study termination or completion.

This study will be conducted in accordance with the ethical principles of:

- World Medical Association Declaration of Helsinki (10)
- International Conference on Harmonisation (ICH) Good Clinical Practice (GCP) guidelines (11)
- Applicable national regulatory requirements, e.g. *Guidelines for Good Practice in the Conduct of Clinical Trials in Human Participants in South Africa* (14)

11.2 Social Harms

Social harms including discrimination, loss of job opportunities, difficulties with insurance or military service, or disruption of family or personal relationships may result due to participation in this study becoming known to others. In addition, investigational product use could potentially not be acceptable to the participant's sex partner and result in difficulties with her spouse or sex partner. If a participant is or becomes HIV-infected, she may also experience social harms.

During each HIV counselling session, participants will be asked questions to address the occurrence of social harms. Participants who experience social harms will be counselled accordingly and provided with assistance to mitigate the circumstances if possible. This will be recorded in the source documents and applicable CRFs.

12.0 PUBLICATION

Any presentation, abstract, or manuscript shall be reviewed and approved by the Sponsor prior to submission. Publication of the results of this study will be governed by the Sponsor's publication policies. Authorship criteria will be based on contributions to the design, work, and analysis of the study.

13.0 REFERENCES

1. Joint United Nations Programme on HIV/AIDS (UNAIDS) and World Health Organization (WHO) 2007 Aids Epidemic Update; December 2007.
2. Wortley RM, Fleming PL. AIDS in women in the United States. Recent Trends. JAMA 1997; 278(11): 911-6
3. Centers for Disease Control. Update: barrier protection against HIV infection and other Sexually Transmitted Diseases. MMWR 1993;42:589-97 (http://www.cdc.gov/MMWR/preview/mmwrhtml/00021321.htm)
4. Stone AB, Hitchcock PJ. Vaginal microbicides for preventing sexual transmission of HIV. AIDS 1994;8:S285-S293
5. Elias CJ, Heise LL. Challenges for the development of female-controlled vaginal microbicides. AIDS 1994;8(1):1-9
6. Elias CJ, Coggins C. Female-controlled methods to prevent sexual transmission of HIV. AIDS 1996;10:S43-S51
7. Dapivirine Ring Investigators Brochure
8. Warner Chilcott Femring® Full U.S. Prescribing Information (http://www.warnerchilcott.com/pdfs/pi-femring(physician).pdf)
9. Warner Chilcott Femring® Patient Information Materials: Patient Acceptability. (http://www.warnerchilcott.com/pdfs/pi-femring(patient).pdf)
10. World Medical Association Declaration of Helsinki. Ethical Principles for Medical Research Involving Human Subjects; WMA General Assembly, Seoul October 2008 http://www.wma.net/e/policy/b3.htm
11. Guidance for Industry: E6 Good Clinical Practice: Consolidated Guidance (http://www.doh.gov.za/docs/policy/trials/trials_contents.html)
12. MMWR 2001; 50 (No. RR-19: 1-58 (or http://www.cdc.gov/mmwr/preview/mmwrhtml/rr5019a1.htm)
13. Pharmacia & Upjohn Estring® Physician Leaflet (http://www.pfizer.com/pfizer/download/uspi_estring.pdf)
14. Guidelines for Good Practice in the Conduct of Clinical Trials in Human Participants in South Africa (http://www.doh.gov.za/docs/policy/trials/trials_contents.html)

APPENDIX A: SCHEDULE OF CLINICAL PROCEDURES

**Study Visit**	**Screening**	**1**[a]	**2**[b]	**3**[b]	**4**[b]	**5**[b]	**6**[b]
**Study Week**		**0**	**2**	**4**	**8**	**12**	**16**
Informed Consent	X	X					
Demographics & Medical History	X	X[c]					
Concomitant Medication Evaluation	X	X[c]	X	X	X	X	X
Inclusion/Exclusion Criteria	X	X[c]					
Locator Information	X	X[c]	X	X	X	X	X
Menses Information	X	X[c]	X	X	X	X	X
Acceptability Assessments		X		X		X	
Adherence Assessments			X	X	X	X	
Behavioural Assessments		X		X	X	X	X
Provision of Diary Card	X						
Review of Diary Card		X	X	X	X	X	
General Physical Examination	X					X	
Pelvic/Speculum Examination	X	X[c]	X	X	X	X	X
Colposcopy		X[c]		X	X	X	
HIV/STI Risk Reduction Counselling	X	X[c]	X	X	X	X	X
Cervico-vaginal Sample Collection for STI	X			X	X	X	
Vaginal Flora and Vaginal pH Specimen		X		X	X	X	
Cervico-vaginal Sample Collection for Pap	X						
HIV Pre- & Post-Test Counselling	X	X[c]	X	X	X	X	X
Intravaginal Ring Adherence Counselling		X	X	X	X		
Contraceptive Counselling	X	X[c]	X	X	X	X	
HIV-1 Rapid Testing[d]	X	X[c]	X	X	X	X	
HIV RNA PCR		X	X[h]	X[h]	X[h]	X[h]	X
Serum Pregnancy Testing						X	
Urine Pregnancy Testing[e]	X	X[c]	X	X	X		X
Laboratory testing[f]	X			X	X	X	
Plasma Dapivirine Levels[i]		X		X		X	
Syphilis Testing	X						
New Intravaginal Ring Insertion		X		X	X		
Intravaginal Ring Removal/Return			X[g]	X	X	X	
Residual Dapivirine Levels				X	X	X	
Adverse Event		X	X	X	X	X	X
Study Completion							X

[a] Study Week 0 (enrolment) must occur within 28 days of first screening visit.
[b] The study window for all visits except for screening and enrolment is ±4 days.
[c] The specified procedures occur prior to enrolment.
[d] Participants who test HIV-1Rapid Test reactive will have a second blood draw (approximately 3 ml blood drawn, if necessary) for confirmatory testing.
[e] In addition to the time points noted, urine pregnancy testing will be conducted in the event the participant misses a menstrual period.
[f] Urinalysis (dipstick, micro), haematology (FBC with diff and platelets), chemistry (electrolytes, calcium), renal (BUN, creatinine), liver (AST, ALT, ALP, GGT, bilirubin).
[g] Ring removal/reinsertion for completion of pelvic/speculum exam.
[h] Sample will be stored and will only be tested if necessary to identify approximate period of HIV infection.
[i] Sample to be taken only if participant is enrolled.

APPENDIX B: INTRAVAGINAL RING INSERTION & REMOVAL INSTRUCTIONS

HOW DO I USE THE INTRAVAGINAL RING?

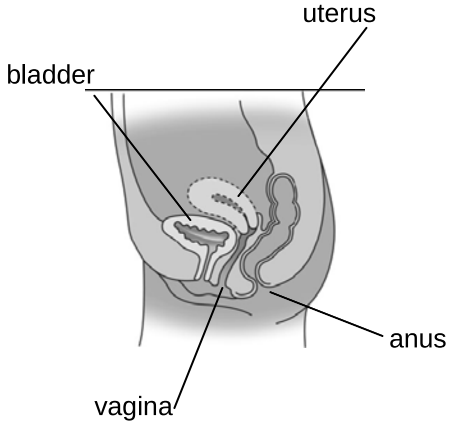

TO INSERT THE RING INTO YOUR VAGINA:

1. Wash and dry your hands.
2. Remove the ring from its package.
3. Choose the position that is most comfortable for you. For example, lying down or standing with one leg up. (See **Diagrams 1a and 1b**, respectively).

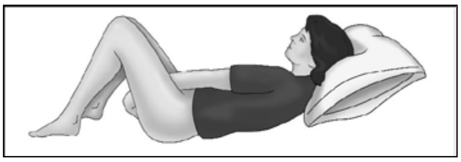

DIAGRAM 1a

OR

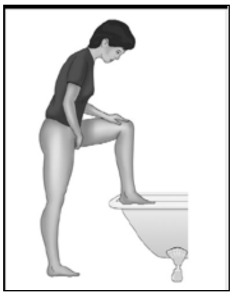

DIAGRAM 1b

4. Use your thumb and index finger (pointer finger) to press the sides of the ring together. You may find it easier to insert the ring if you twist it into a figure-of-eight shape. (See **Diagram 2**)

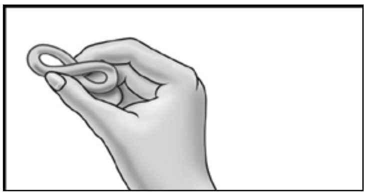

DIAGRAM 2

5. Use your other hand and hold open the folds of skin around your vagina. (See **Diagram 3**)

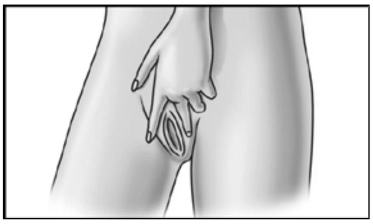

DIAGRAM 3

6. Place the tip of the ring in the vaginal opening and then use your index finger to push the folded ring gently into your vagina. Push it up towards your lower back as far as you can. (See **Diagram 4**)

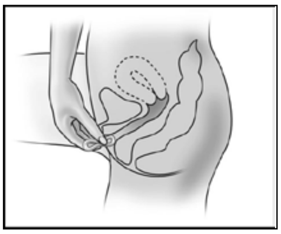

DIAGRAM 4

If the ring feels uncomfortable, you probably did not push it into your vagina far enough. Use your index finger to push the ring as far as you can into your vagina (See **Diagram 5**). There is no danger of the ring being pushed too far up in the vagina or getting lost.

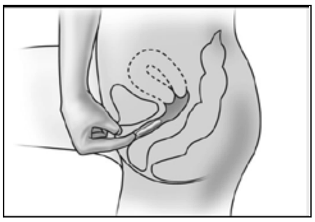

DIAGRAM 5

The ring should now be in your upper vagina (See **Diagram 6**).

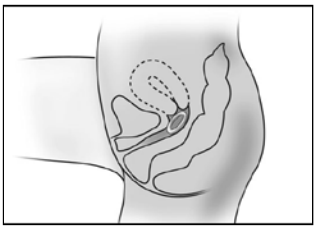

DIAGRAM 6

7. Wash your hands when you are done.

TO REMOVE THE INTRAVAGINAL RING:

1. Wash and dry your hands.
2. Choose the position that is most comfortable for you (See **Diagrams 1a** and **1b**).

3. Put a finger into your vagina and hook it through the ring. (See **Diagram 7**)

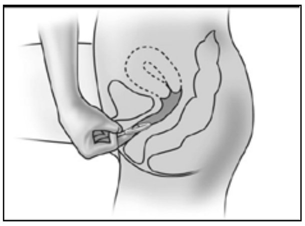

DIAGRAM 7

4. Gently pull downwards and forwards to remove the ring.
5. Wrap the used ring in tissue or toilet paper and give it to the clinic staff for disposal.
6. Wash your hands.

IMPORTANT INFORMATION

- If possible, you should try not to remove the ring except as directed during study visits. If the ring accidentally comes out of your vagina before your next clinic visit, e.g., during sex, clean it with warm water and put it back in your vagina.
- If you have any problems putting the ring back in your vagina, call or come to the clinic.

Screening ID Number **Participant Initials**

APPENDIX C: SAMPLE <u>SCREENING</u> CONSENT FORM TEMPLATE

A DOUBLE-BLIND, RANDOMISED, PLACEBO-CONTROLLED PHASE I/II STUDY TO EVALUATE THE SAFETY OF AN INTRAVAGINAL MATRIX RING WITH DAPIVIRINE IN HEALTHY HIV-NEGATIVEWOMEN

Insert study research centre/host country and the International Partnership for Microbicides (IPM) are conducting a research study. IPM is a non-profit product development partnership that is working towards developing a safe and effective vaginal microbicide for use by women in developing countries to prevent HIV transmission. Vaginal microbicides are medicines being developed to prevent HIV infection.

In this study healthy sexually active women will be asked to use an intravaginal dapivirine ring or a matching placebo intravaginal ring and be asked their opinion about its use. Intravaginal rings have previously been used to deliver medicines to prevent pregnancy and to treat menopause. Research is being conducted to see if the ring can be used to deliver a vaginal microbicide (dapivirine) to prevent HIV infection in women. Vaginal microbicides are medicines being developed to prevent HIV infection. **<u>The ring being used in this particular study may contain dapivirine or it may contain no dapivirine and is not proven to protect you from HIV or any other sexually transmitted infection.</u>** The ring used in this study has not been previously studied in humans. This study (called the "Ring Study") is being conducted to see if the ring containing dapivirine is safe.

You are not being asked to join in the ring study right now. You are being asked to join in a Screening process to see if you meet the requirements to join the ring study. You will be given information about the ring study and told what it means to join the study. If you meet the requirements and are willing to take part in the ring study, you will be given the chance to join later.

Before you decide if you want to join this 28 day Screening process or not, we want to explain the study, its risks, its potential benefits, and what you will be asked to do. You may ask questions as we discuss the study, so that you understand what the study is about. It is important you know the following:

- Joining the Screening process is your decision (voluntary) and not up to anyone else.
- You will not lose any of your routine medical care benefits if you decide not to join the Screening process.
- You may join the Screening process and then change your mind and leave the study at any time.

If you agree to join this Screening process, we will ask you to sign and date this consent form. We will give you a copy of this form to keep.

<u>HOW WILL THE SCREENING PROCESS WORK?</u>

Screening ID Number

Participant Initials

After you sign this consent form, the following will happen:

1. **Interview** – You will be asked questions about yourself including your health, medical history, medicines taken, menses information, and where we can contact you. You will also be asked some questions to see if you are eligible for the ring study.
2. **Counselling** – You will meet in private with a counsellor who will talk with you and provide information about how to prevent or reduce your risk of infection with HIV or other sexually transmitted infections. You will be provided with condoms. The counsellor will talk to you before your HIV test and after your test results are ready. The counsellor will also discuss birth control with you. **You will not be allowed to use a vaginal barrier method, for example, a diaphragm or cervical cap.**
3. **HIV Testing** – You will have blood drawn to test for HIV. If the test shows that you are HIV-non reactive you may be eligible to join the study. If the test shows you have HIV, you cannot join the ring study and no more procedures will be done. However the nurse or doctor may need to draw blood again to confirm the test results. The nurse or doctor may also need to take a small amount of blood from your arm (less than 1 teaspoon) to confirm the test results. You will be referred to local health facilities for social support or other medical services as needed.
   NOTE: *If the national regulatory authority of the country in which the research centre is situated requires that a national testing algorithm be used during the screening process, this will be performed as part of the screening process.*
4. **Pregnancy Testing** – You will have a pregnancy test. You will give a sample of your urine to test if you are pregnant. If you are pregnant, you cannot join the ring study and no more procedures will be done. You will be referred to the local prenatal clinic for support services.
5. **Physical Exam, Pelvic Exam, and Lab Testing** – You will have a physical exam and a pelvic exam to check inside your vagina for any infections, ulcers or sores. The nurse or doctor will use a swab to take samples from your vagina which will be sent to a lab to check for genital infections. You will also have samples taken for a Pap smear to check for cervical cancer. (If you are menstruating at this screening visit, the pelvic exam and collection of vaginal samples will be rescheduled to another visit after completion of your menses). If you have symptoms or findings of a genital infection, you will be provided treatment, if available, or referred to local health facilities for treatment as needed. A sample of urine will be obtained and a blood sample of 15 ml (3 teaspoons) will be drawn from your arm and sent to a lab for analysis. If your exams show you are still eligible for the study, you will be given a date to return to the clinic within 28 days for the enrolment visit. (NOTE: You should NOT be menstruating at the enrolment visit. If you are, tell the study staff and the visit will be rescheduled).

The visit will take about **X amount time** to complete. You will receive **X amount** for your time and travel expenses.

IF I AM STILL ELIGIBLE FOR THE RING STUDY, WHAT HAPPENS?
At the time of the enrolment visit (within 28 days from the screening visit), you will first undergo some pre-enrolment procedures as follows:

Screening ID Number

Participant Initials

- **Interview** – You will be asked about any medical problems and/or medicines you have taken since your last visit, menses information, and where we can contact you
- **Counselling** – You will meet in private with a counsellor who will talk with you and provide information about how to prevent or reduce your risk of infection with HIV or other sexually transmitted infections. You will be given condoms. The counsellor will also discuss birth control and advise you to continue with your current method of birth control throughout the study. **You will not be allowed to use a vaginal barrier method, for example, a diaphragm or cervical cap**.
- **HIV Testing** – You will have blood drawn to test for HIV. If the test shows that you are HIV negative you may be eligible to join the study. You will then be asked to give another blood sample (approximately 5ml) to test for HIV using a different type of test. This sample will only be tested if you enrol into the study. If the first test shows you have HIV, you cannot join the ring study and no more procedures will be done. However the nurse or doctor may need to draw blood again to confirm the test results. The nurse or doctor may also need to take a small amount of blood from your arm (less than 1 teaspoon) to confirm the test results. You will be referred to local health facilities for social support or other medical services as needed.
  NOTE: If the national regulatory authority of the country in which the research centre is situated requires that a national testing algorithm be used during the screening process, this will be performed as part of the screening process.
- **Plasma Dapivirine Levels –** A plasma sample (pre-dose / before the ring is inserted) will be taken by venipuncture approximately 10ml (2 teaspoon) for dapivirine measurement.
- **Pregnancy Testing** – You will give a sample of your urine (screening & Visits 1 - 4 & 6) or a teaspoon = 5ml of blood at Visit 5 to test if you are pregnant. If you are pregnant, you cannot join the ring study and no more procedures will be done. You will be referred to the local prenatal clinic for support services.
- **Pelvic Exam & Colposcopy** – If your pregnancy test is negative, you will have a pelvic exam and colposcopy to check inside your vagina for any infections, ulcers or sores. Eligible participants will be asked to abstain from the following 72 hours prior to the examination:
  - Vaginal intercourse
  - Oral contact with her genitalia
  - Internal vaginal washing
  - Penetration of the vagina by fingers, sex toys, or any other objects

  At the time of the examination, any sexual activity reported within the previous 72 hours will be documented. This is being asked to help us to know if any problems that might be seen could be caused by the ring or whether they could be caused by something else.

  A colposcope is an instrument which uses a magnifying lens to examine your vaginal tissue. A swab of your vagina will also be taken and sent for testing if you are enrolled in the study. If your exam shows that you still have infections, ulcers or sores 28 days after your initial screening visit, you will be provided treatment, if available, or referred to local health facilities for treatment as needed but you will not be eligible for the ring study.

Screening ID Number

Participant Initials

- **Diary Card** – During this visit you will be provided with a diary card and the completion of the diary card will be explained to you. Questions will be asked with regard to ring use, condom use, sexual acts and the answers will have to be completed using the diary card daily. The completed diary card will have to be taken to the research centre at each visit as it will be reviewed by the research centre staff and it will also contain your appointment dates as well as the investigator information.

If your exam shows that you are still eligible, you will be invited to join in the ring study right away, and be requested to sign a separate study consent form. However, you will be under no obligation to join in the ring study and may freely refuse to do so. Regardless of whether you decide to join the ring study or not, the visit will take about **X amount time** to complete and you will receive **X amount** for your time and travel expenses.

By signing this consent form, you have been told that if you are eligible to return for the enrolment visit but do not show up, you have given permission for clinic staff to contact you.

WHAT ARE THE POTENTIAL RISKS OF THIS SCREENING PROCESS?
There are no serious risks associated with joining in this screening eligibility study. You may feel discomfort during the pelvic exam and/or colposcopy, and there is a very small risk of injury to the lining inside your vagina from the speculum. You may become embarrassed, worried, or anxious during the pelvic or physical exams, or when discussing your health, sexual behaviours, or as a result of being tested for HIV infection and counselled. You may become worried or anxious while waiting for results of your tests for HIV or other infections. If you have a genital infection, you might experience problems with your partner(s). You may feel discomfort from the blood draws for your HIV test. If you have a reactive HIV test and need to have blood drawn to confirm your result, you may feel discomfort when blood is drawn and you may feel dizzy or faint and may later have a bruise or swelling (and rarely, an infection) where the needle goes in your finger or arm.

WHAT ARE THE POTENTIAL BENEFITS OF THIS SCREENING PROCESS?
At no cost to you, you will have your health evaluated, receive medical exams and a lab test to check for genital infections and be offered treatment or referred outside of the study for medical care.

WHAT ARE THE COSTS OF THE SCREENING PROCESS?
There is no cost to you for taking part in the study. You will be reimbursed **X amount** for your time and travel.

WHO WILL SEE MY PERSONAL & MEDICAL INFORMATION?
We will do everything we can to protect your privacy. You will be assigned a code number (instead of your name) which will be used on all information collected about you on this study. A master list with your name and code will be kept under lock and key at the clinic where you are enrolled. Your name will never be used in any publication or presentation about this study. However people or groups that may review your records include the U.S. Food and Drug Administration (FDA),

Screening ID Number

Participant Initials

Ethics Committees, regulatory authorities, study monitors, the manufacturing company or the Sponsor of the study (IPM). By signing this written informed consent form, you or your legally acceptable representative authorize such access to your records.

If you give your permission, your personal doctor will be informed that you are taking part in this study.

WHAT HAPPENS IF I AM INJURED?

If you become ill or injured as a result of taking part in the Screening process, you will receive medical treatment free of charge. The study staff also will tell you where you can get additional treatment, if needed. We will pay your reasonable medical costs for treatment of any illness or injury that is associated with your participation in the Screening process.

For South African use only:

Compensation will be according to the Association of the British Pharmaceutical Industry (ABPI) guidelines. The doctor will have a copy of these guidelines should you wish to see it.

WHAT IF I HAVE QUESTIONS OR PROBLEMS?

If you have any questions, you can contact a member of the clinic staff or Dr.___________ <PI>________ Tel___<24 hr contact number>_. This research study has been reviewed and approved by <Ethics Committee>, to ensure that the rights and safety of all participants in the study are upheld and that the study is conducted according to strict guidelines. If at any time you have any questions regarding your rights as a participant in a research study, you may contact <Ethics Committee Chairperson> Tel: <contact number and address>.

Screening ID Number **Participant Initials**

SIGNATURE TO SCREEN FOR THE IPM 015 STUDY:

I have read this consent form (or had it explained to me), and all of my questions have been answered to my satisfaction. I know that I can refuse to join this Screening process, or if I agree to join I can drop out of the Screening process at any time without losing any benefits or services to which I am entitled. After signing below, I will receive a copy of this consent form. My signature (or thumbprint or mark) below confirms that I freely agree to **SCREEN** for this study.

____________________________ ____________________________

Volunteer’s Name (print) Volunteer’s Signature/Thumbprint/Mark & Date

____________________________ ____________________________

Name of Staff Conducting Consent Session (print) Staff Signature & Date

____________________________ ____________________________

Investigator or Co-Investigator (print) Investigator or Co-investigator Signature & Date

____________________________ ____________________________

Witness’ Name (print) (AS APPROPRIATE) Witness’s Signature & Date

Participant ID Number

Participant Initials

APPENDIX D SAMPLE STUDY CONSENT FORM TEMPLATE

A DOUBLE-BLIND, RANDOMISED, PLACEBO-CONTROLLED PHASE I/II STUDY TO EVALUATE THE SAFETY OF AN INTRAVAGINAL MATRIX RING WITH DAPIVIRINE IN HEALTHY HIV-NEGATIVE WOMEN

Insert study research centre/host country and the International Partnership for Microbicides (IPM) are conducting a research study. IPM is a non profit product development partnership that is working towards developing a safe and effective vaginal microbicide for use by women in developing countries to prevent HIV transmission. Vaginal microbicides are medicines being developed to prevent HIV infection.

In this study, healthy sexually active women who will use an intravaginal ring and be asked their opinion about its use. Intravaginal rings have previously been used to deliver medicines to prevent pregnancy and to treat menopause. Research is being conducted to see if the ring can be used to deliver a vaginal microbicide (dapivirine) to prevent HIV infection in women. Vaginal microbicides are medicines being developed to prevent HIV infection. **The ring being used in this particular study may contain dapivirine or it may contain no dapivirine and is not proven to protect you from HIV or any other sexually transmitted infection. The ring used in this study has not been previously studied in humans.** This study (called the "Ring Study") is being conducted to see if the ring containing dapivirine is safe. This consent form will give you information to help you decide if you would like to join the ring study.

Before you decide if you want to join this ring study or not, we want to explain the study, its risks, its potential benefits, and what you will be asked to do. You may ask questions as we discuss the ring study, so that you understand what the study is about. It is important you know the following:

- Joining the ring study is your decision (voluntary) and not up to anyone else.
- You will not lose any of your routine medical care benefits if you decide not to join the ring study.
- You may join the ring study and then change your mind and leave the study at any time.

If you agree to join this ring study, we will ask you to sign and date this consent form. We will give you a copy of this form to keep.

WHAT IS THE STUDY FOR?

The main purpose of the study is to see if the intravaginal ring containing dapivirine is safe to use in women. The other purpose of this study is to observe and document if there are any differences in vaginal problems that you may experience while using the ring between women who use an intravaginal matrix ring that contains dapivirine and women who use an intravaginal ring that contains

Participant ID Number

Participant Initials

no dapivirine. This will help to identify if any problems are due to the use of dapivirine.

At this time, no one knows whether the microbicide intravaginal ring called Dapivirine intravaginal ring will protect you from HIV infection. For this reason, during the study it is very important that you use condoms provided by the research centre, each time you have sex. In addition to condoms, you will also be required to be on some other type of hormonal contraception or IUD if you are able to get pregnant. Because Dapivirine intravaginal ring is a new product that is being tested, its safety for use during pregnancy has not yet been shown. Therefore, you will be tested at every study visit for pregnancy and will be asked to stop use of the intravaginal ring immediately if you are found to be pregnant.

This study is also being done to find out if Dapivirine intravaginal ring is acceptable to women and if you would use it in the future; how well women are able to use the study product for 12 weeks; and whether Dapivirine intravaginal ring has any effect on the normal bacteria in the vagina.

WHO WILL BE IN THE STUDY?
About 280 healthy women between the ages of 18 and 40 years from up to 14 research centres in Kenya, Malawi, Rwanda, South Africa, Tanzania and Zambia will join this study which is expected to last approximately 6 months total, or perhaps longer, depending on how long it takes for 280 women to join the study.

WHAT IS THE INTRAVAGINAL RING?
The intravaginal ring is made of a silicone rubber material that is the same material used in Femring® estradiol acetate intravaginal ring, a U.S. Food and Drug Administration approved intravaginal ring containing a medication for treatment of symptoms associated with menopause. The same ring and hormone replacement drug (Menoring® vaginal ring) is approved in the United Kingdom.

The intravaginal rings are off-white, soft, and flexible. Studies have shown that the ring itself appears safe, non-toxic and non-irritating.

Two types of intravaginal rings will be used in this study:
Dapivirine Ring, which has dapivirine mixed throughout the ring.
Placebo Ring, which contains no dapivirine.

WHAT IS DAPIVIRINE?
The medicine being used in this study is called dapivirine. It is in the process of development to prevent sexual transmission of the Human Immunodeficiency Virus (HIV) to women. This virus may lead to Acquired Immune Deficiency Syndrome (AIDS). Dapivirine is not approved for use by the Regulatory Authorities that evaluate whether medication is suitable for human use. Therefore, it can only be used in research studies.

Dapivirine belongs to a group of medications used in the treatment of HIV called non-nucleoside reverse transcriptase inhibitors (NNRTIs). This type of medication

Participant ID Number

Participant Initials

inhibits the multiplication of the virus. Dapivirine has not yet been proven to protect you against HIV.

WHAT IS THE STUDY REGIMEN?

If you decide to enrol in this study, you will be asked to use an intravaginal ring for 12 weeks and will be followed up at the clinic. You will have to return to the clinic at 2 weeks, 4 weeks, 8 weeks, and 12 weeks for visits and tests. You will be given a new ring to use every 28 days. Your last clinic visit will be 4 weeks after you have stopped using the ring. Total study participation is 16 weeks.

If you join the study, you will be put into 1 of 2 groups by chance (like the toss of a coin). One group of women will use an intravaginal matrix ring that contains dapivirine. The other group of women will use an intravaginal ring that does not contain dapivirine (a placebo ring). All the women in both groups will use an intravaginal ring for 12 weeks and will undergo the same tests. Neither you, nor the study staff, nor the sponsor (IPM) will know which group you are in.

HOW LONG IS THE STUDY?

There are 6 scheduled visits (including the enrolment visit) which take place over a 16 week period. After enrolling into the study, you will return to clinic 2, 4, 8, 12 and 16 weeks after enrolment. Most visits will take about **X** hours to complete but sometimes a study visit may take a little longer. You may need to come back to clinic for additional visits if you have any medical problems or concerns.

ONCE I AM CONSIDERED ELIGIBLE FOR THE RING STUDY, WHAT HAPPENS?

After you have passed all the screening and pre-enrolment procedures described earlier in the Screening Consent form and have signed this study consent form, you will be enrolled into the study and put into either 1 of 2 groups by chance (like the toss of a coin). One group of women will use the intravaginal matrix ring that contains dapivirine; the other group of women will use the intravaginal ring that does not contain dapivirine. Neither you, nor the study staff, nor the sponsor (IPM) will know which group you are in. The following describes what will happen at specific visits. Both groups of women will undergo exactly the same procedures. (NOTE: the procedures below do not necessarily occur in the order listed):

- **Intravaginal Ring Insertion – Enrolment & 4 and 8 Weeks After Enrolment**
  Study staff will give you an intravaginal ring and show you how to insert the ring yourself and you will do this at the clinic with staff help, if needed. You will have a brief examination to confirm that the ring is in place.
- **Intravaginal Ring Adherence Counselling** – **Enrolment & 2, 4, and 8 Weeks After Enrolment**
  You will be counselled to use the ring for the 12 week period and not to take it out at anytime except when directed at study visits. You will be given instructions on how to re-insert the ring if it comes out accidentally. You will also be told not to use other vaginal products or other objects.
- **Assessments**

Participant ID Number **Participant Initials**

You will be asked questions regarding your opinion about using the intravaginal ring and if you would use the ring in the future, ease of use, comfort, cleanliness, and effect on sex as well as your partner's reactions. You will also be asked questions about whether you would use this ring if it contained a vaginal microbicide approved for protection against HIV infection.
Acceptability will be done at **Enrolment, 4 and 12 Weeks After Enrolment.**
Adherence will be done at **2, 4 8 and 12 Weeks After Enrolment**
Behavioural will be done at **Enrolment, 4, 8, 12 and 16 Weeks After Enrolment**
Diary Card will be reviewed at **Enrolment, 2, 4, 8 and 12 Weeks After Enrolment**

- **Intravaginal Ring Removal – 4, 8 and 12 Weeks After Enrolment**
  The study staff will show you how to remove the ring yourself and you will do this at the clinic with staff help if needed. The staff will store the used and unused rings until they are requested to ship them to an analytical laboratory for measurement of dapivirine levels.
- **Pelvic Exam** – **2, 4, 8, 12 and 16 Weeks After Enrolment**
  You will have a pelvic exam to check inside your vagina for any problems. Before you have the exam, at each scheduled visit except at 16 weeks after enrolment, the study staff will show you how to remove the ring yourself and you will do this with staff help if needed. After the exam, you will reinsert the ring yourself with staff help if needed. At weeks 4 and 8 you will be given a new ring to insert. At Week 12 you will have finished using the ring and will not insert a ring after the pelvic exam.
- **Colposcopy** – **4, 8 and 12 Weeks After Enrolment**
  In addition to the pelvic exam at these visits, the doctor will use a colposcope, which is an instrument with a magnifying lens, to check inside your vagina for any problems.
  You will be asked to abstain from the following 72 hours prior to the examination:
    - Vaginal intercourse
    - Oral contact with her genitalia
    - Internal vaginal washing
    - Penetration of the vagina by fingers, sex toys, or any other objects

  At the time of the examination, any sexual activity reported within the previous 72 hours will be documented. This is being asked to help us to know if any problems that might be seen could be caused by the ring or whether they could be caused by something else.

  Laboratory Tests – 4, 8 and 12 Weeks After Enrolment
  At 4, 8 and 12 weeks after enrolment**,** your vagina will be swabbed for samples and sent to a lab to check for genital infections, a sample of urine will be obtained and 15 ml (3 teaspoons) of blood will be drawn from your arm and sent to a lab for analysis.
- **Plasma Dapivirine Levels – 4 & 12 Weeks After Enrolment**
  At 4 & 12 weeks after enrolment a plasma sample will be taken by venipuncture approximately 10 ml (2 teaspoon) for dapivirine measurement.
- **Interview –2, 4, 8, 12, and 16 Weeks After Enrolment**
  You will be asked about any medical problems and/or medicines you have taken since your last visit, menses information, and where we can contact you.

Participant ID Number **Participant Initials**

- **HIV & Contraceptive Counselling – 2, 4, 8 and 12 Weeks After Enrolment**
  You will meet in private with a counsellor who will talk with you and provide information about how to prevent or reduce your risk of infection with HIV or other sexually transmitted infections. You will be given condoms. The counsellor will also discuss birth control and advise you to continue on your current stable form of birth control for the duration of the study. **You will not be allowed to use a vaginal barrier method, for example, a diaphragm or cervical cap.**
  At all scheduled visits except 16 weeks after enrolment, the counsellor will also talk to you before your HIV test and after your test results are ready.
- **HIV Testing – 2, 4, 8, 12 and 16 Weeks After Enrolment**
  At weeks 2, 4, 8, and 12 you will give a sample of blood to test for HIV. If you are still HIV negative, you will continue to use the intravaginal ring. A sample of blood (about 1 teaspoon) will be taken and kept in a laboratory in case you become HIV positive later while on the study. If the first test shows that you might be positive, another test will be done to check the result. The doctor or nurse may also have to send a sample of blood away to confirm the result and obtain additional information on typing of the virus. . If the result is not clear after the first 2 tests, you may be asked to come back earlier than your next visit for another test. At the last visit at week 16, a sample of blood (about 1 teaspoon) will be taken and sent to a laboratory to check for HIV infection. If you become positive, other blood samples may be taken to obtain additional information about the HIV virus and you will be offered the option of rolling over into another trial.
- **Pregnancy Testing – 2, 4, 8, 12 and 16 Weeks After Enrolment**
  You will give a sample of your urine to test if you are pregnant. If you are pregnant you will be referred to the local prenatal clinic for support services. NOTE: You will also have pregnancy testing during the study any time you miss a menstrual period.

NOTE: You should NOT be menstruating at any visit. If you are or you think that you will be menstruating at a scheduled visit, tell the study staff and the visit will be rescheduled.

You will be reminded to contact or return to clinic if you have any medical problems or concerns anytime during the study including if you accidentally expel or lose the intravaginal ring. Each visit will take about **X amount time** to complete. You will receive **X amount** for your time and travel expenses.

We will use the contact information you provide to remind you of scheduled visits. If you miss a visit, the study staff will try to contact you by phone or other methods. They will try to reach you at home or through family, friends or authorities. If they talk to these people, they will not tell them why they are trying to reach you.

ARE THERE ADDITIONAL STUDY REQUIREMENTS DURING THE STUDY?
Do not use products or objects which are inserted into the vagina such as tampons, cotton wool, rags, diaphragms, cervical caps, douches and drying agents while you are participating in the study.

Participant ID Number **Participant Initials**

WHAT IF I BECOME HIV-INFECTED DURING THE STUDY?

After you have enrolled in the study, if your HIV test is reactive, the nurse or doctor will use a needle to draw blood again and, if necessary, the nurse or doctor may also need to take a small amount of blood from your arm (less than 1 teaspoon**)** to confirm the test results. You will be referred to local health facilities for social support or other medical services as needed. If you are confirmed to be infected, you will be offered treatment for HIV infection based on your medical needs according to your country's treatment guidelines. If those guidelines are unavailable, the guidelines of the World Health Organization (WHO) will be followed. **If national or local ARV treatment programs are not in place, IPM will pay for ARV treatment until such resources are available.** You will continue with your regular scheduled visits to assess safety.

WHAT IF I BECOME PREGNANT DURING THE STUDY?

Since we do not know whether the intravaginal ring or dapivirine has any effect on pregnancy, or whether it can harm the foetus, pregnant women cannot join this study. If you are a woman able to become pregnant, you must be on one of the following contraception methods:

- Taking oral contraceptive pills for at least 3 months prior to enrolment or,
- Using a contraceptive patch for at least 3 months prior to enrolment'
- On a long-acting birth control method like injectable Depo-Provera® for 6 months'
- Have had an IUD inserted (with no vaginal or gynaecological complaints associated with its use) for at least 3 months prior to enrolment or,
- Have undergone surgical sterilization at least 3 months prior to joining the study.

You must agree to continue using birth control (which is not a vaginal barrier method like a diaphragm or cervical cap) during this study (unless you have been sterilized) and you will have a pregnancy test during screening, enrolment and all scheduled visits. You will also have pregnancy testing during the study any time you miss a menstrual period.

If you find out you are pregnant or you are confirmed to be pregnant during a clinic visit, you will immediately remove the ring and will no longer use it. If applicable, bring the ring back to the clinic for proper disposal. You will be referred to a local prenatal clinic for support services. You will continue with your regular scheduled visits to assess safety and thereafter, information will be collected regarding your pregnancy and delivery and your child's first year of life.

WHAT HAPPENS IF I WANT TO WITHDRAW FROM THE STUDY EARLY?

If you choose not to continue in the study before it ends, you will be asked to return for one last study visit. You do not have to return for this visit. If you do return for this visit, study procedures performed at the last visit as described above will be performed.

WHAT ARE THE RISKS/DISCOMFORTS OF THIS STUDY?

Participant ID Number **Participant Initials**

Some of the risks and/or discomforts that you should consider before agreeing to take part in this study are described as follows. If you have any problems during the study, you should contact the study staff right away.

It is not known what effects dapivirine will have on the vagina. Possible effects include vaginal dryness, itching, redness, burning, bleeding or pain. It is possible that a small amount of dapivirine could get into the bloodstream from the vagina, but this is not expected to cause any problems. It is possible that using dapivirine could make some anti-HIV medicines less effective. This could affect you if you become infected with HIV and need to take anti-HIV medicines in the future.

It is possible that you are allergic to the material (silicone rubber) used to make the intravaginal ring. It is possible that you may experience local irritation or vaginal complaints as a result of using the ring. You may feel discomfort during the pelvic exam and/or colposcopy, and there is a very small risk of injury to the lining inside your vagina from the speculum. You may become embarrassed, worried, or anxious during the pelvic or physical exams, or when discussing your health, sexual behaviours, or HIV infection; or answering questions about use of the ring before, during or after sex. You may become worried or anxious while waiting for results of your tests for HIV and other infections. You may feel discomfort from the finger prick for your HIV test. If you have a reactive HIV test and need to have blood drawn to confirm your result, you may feel discomfort from the finger prick or when blood is drawn and you may feel dizzy or faint and may later have a bruise or swelling (and rarely, an infection) where the needle goes in your finger or arm. If you have a genital infection and need to tell your partner(s) you might experience problems. You might experience problems with your partner(s) related to your using (or attempting to use) the ring. You have been told your joining in this study may be associated with study-related discrimination. Discrimination may arise if you choose to tell family, friends, co-workers or others about you joining the study. You may suffer discrimination if others think you are at risk for HIV-1 infection or are HIV-1 infected because you joined in the study. Our staff can advise you on dealing with such problems. We will make every effort to protect your privacy and confidentiality during the study and help you deal with any uncomfortable feelings or questions you may have

WHAT ARE THE BENEFITS OF THIS STUDY?
This study may be of no direct benefit to you. However, you or others may benefit in the future from information learned from this study about how intravaginal rings may be used with topical microbicides to prevent HIV infection. At no cost to you, you will have your health evaluated, receive medical exams and a lab test to check for genital infections and be offered treatment or referred outside of the study for medical care.

WHAT IF THERE IS NEW INFORMATION ABOUT THIS STUDY?
During the course of the study, you will be told about any important new findings that may be beneficial or harmful to you and that might influence your willingness to continue joining in the study.

Participant ID Number

Participant Initials

WHY WOULD I BE REMOVED FROM THE STUDY EARLY?
The study doctor may end your participation in the study early (even if you want to continue) if: you are not able to attend the study visits or to complete the required study procedures or comply with required study procedures; or the study is cancelled by your research centre's Institutional Review Board (IRB)/Independent Ethics Committee (IEC), or a government authority; or the Sponsor decides to stop the study for any reason.

DO I HAVE OPTIONS OTHER THAN JOINING THIS STUDY?
You can choose not to join this study at all.

IS THERE ANY COST FOR ME TO BE IN THIS STUDY?
You do not have to pay anything for the intravaginal ring, for the study-related clinic visits, physical and pelvic exams, or laboratory tests. These are all free of charge.

DO I RECEIVE ANYTHING FOR MY TIME & TRAVEL?
You will be given **X amount** for your time and travel expenses.

WHO WILL SEE MY PERSONAL & MEDICAL INFORMATION?
We will do everything we can to protect your privacy. You will be assigned a code number (instead of your name) which will be used on all information collected about you on this study. A master list with your name and code will be kept under lock and key at the clinic where you are enrolled. Your name will never be used in any publication or presentation about this study. However people or groups that may review your records include regulatory authorities like your country's health agency or the U.S. Food and Drug Administration (FDA), Ethics Committees, study monitors, the manufacturing company or the Sponsor of the study (IPM). By signing this written informed consent form, you or your legally acceptable representative authorized such access to your records. If you give your permission, your personal doctor will be informed that you are taking part in this study.
If you give your permission, your personal doctor will be informed that you are taking part in this study.

WHAT HAPPENS IF I AM INJURED?
If you become ill or injured as a result of using the intravaginal ring or for any other procedure that is part of the study, you will receive medical treatment free of charge. The study staff also will tell you where you can get additional treatment, if needed. We will pay your reasonable medical costs for treatment of any illness or injury that is associated with your joining in the study.
If you suffer from an injury that is caused directly by your participation in the study (while inserting the intravaginal ring or having any of the study procedures, or having any other medical procedure because of a side effect of using the intravaginal ring), you may be able to receive some compensation. The compensation will only be given if the injury is serious and the effects of it will last for a long time.

Participant ID Number

Participant Initials

For South African use only:
Compensation will be according to the Association of the British Pharmaceutical Industry (ABPI) guidelines. The doctor will have a copy of these guidelines should you wish to see it.

WHAT IF I HAVE QUESTIONS OR PROBLEMS?
If you have any questions, you can contact a member of the clinic staff or Dr._________<PI>___________ Tel_____<24hr contact number> _____________. This research study has been reviewed and approved by _____<Ethics Committee> ___, to ensure that the rights and safety of all participants I the study are upheld and that the study is conducted according to strict guidelines. If at any time you have any questions regarding your rights as a participant in a research study, you may contact <Ethics Committee Chairperson > Tel: <contact number__________________ and address>.

Participant ID Number

Participant Initials

SIGNATURE TO JOIN THE IPM 015 RING STUDY:

I have read this consent form (or had it explained to me), and all of my questions have been answered to my satisfaction. I know that I can refuse to join this study, or if I agree to join I can drop out of the study at any time without losing any benefits or services to which I am entitled. After signing below, I will receive a copy of this consent form. My signature (or thumbprint or mark) below confirms that I freely agree to **JOIN** this study.

_______________________________ _______________________________
Participant's Name (print) Participant's Signature/Thumbprint/Mark & Date

_______________________________ _______________________________
Name of Staff Conducting Consent Session (print) Staff Signature & Date

_______________________________ _______________________________
Investigator or Co-investigator (print) Investigator or Co-Investigator Signature & Date

_____________________________________ _____________________
Witness' Name (print) (AS APPROPRIATE) Witness's Signature & Date
